# Multi-modal and multi-model interrogation of large-scale functional brain networks

**DOI:** 10.1016/j.neuroimage.2023.120236

**Published:** 2023-08-15

**Authors:** Francesca Castaldo, Francisco Páscoa dos Santos, Ryan C Timms, Joana Cabral, Jakub Vohryzek, Gustavo Deco, Mark Woolrich, Karl Friston, Paul Verschure, Vladimir Litvak

**Affiliations:** a Wellcome Centre for Human Neuroimaging, UCL Queen Square Institute of Neurology, London, United Kingdom; b Eodyne Systems SL, Barcelona, Spain; c Department of Information and Communication Technologies, Universitat Pompeu Fabra, Barcelona, Spain; d Life and Health Sciences Research Institute (ICVS), School of Medicine, University of Minho, Braga, Portugal; e ICVS/3B's - Portuguese Government Associate Laboratory, Braga/Guimarães, Portugal; f Centre for Eudaimonia and Human Flourishing, Linacre College, University of Oxford, United United Kingdom; g Centre for Brain and Cognition, Computational Neuroscience Group, Universitat Pompeu Fabra, Barcelona, Spain; h Institució Catalana de la Recerca i Estudis Avançats (ICREA), Barcelona, Spain; i Department of Neuropsychology, Max Planck Institute for Human Cognitive and Brain Sciences, Leipzig, Germany; j School of Psychological Sciences, Monash University, Melbourne, Australia; k Wellcome Centre for Integrative Neuroimaging, University of Oxford, Oxford, United Kingdom; l Donders Institute for Brain, Cognition and Behaviour, Radboud University, Nijmegen, The Netherlands

## Abstract

Existing whole-brain models are generally tailored to the modelling of a particular data modality (e.g., fMRI or MEG/EEG). We propose that despite the differing aspects of neural activity each modality captures, they originate from shared network dynamics. Building on the universal principles of self-organising delay-coupled nonlinear systems, we aim to link distinct features of brain activity - captured across modalities - to the dynamics unfolding on a macroscopic structural connectome.

To jointly predict connectivity, spatiotemporal and transient features of distinct signal modalities, we consider two large-scale models - the Stuart Landau and Wilson and Cowan models - which generate short-lived 40 Hz oscillations with varying levels of realism. To this end, we measure features of functional connectivity and metastable oscillatory modes (MOMs) in fMRI and MEG signals - and compare them against simulated data.

We show that both models can represent MEG functional connectivity (FC), functional connectivity dynamics (FCD) and generate MOMs to a comparable degree. This is achieved by adjusting the global coupling and mean conduction time delay and, in the WC model, through the inclusion of balance between excitation and inhibition. For both models, the omission of delays dramatically decreased the performance. For fMRI, the SL model performed worse for FCD and MOMs, highlighting the importance of balanced dynamics for the emergence of spatiotemporal and transient patterns of ultra-slow dynamics. Notably, optimal working points varied across modalities and no model was able to achieve a correlation with empirical FC higher than 0.4 across modalities for the same set of parameters. Nonetheless, both displayed the emergence of FC patterns that extended beyond the constraints of the anatomical structure. Finally, we show that both models can generate MOMs with empirical-like properties such as size (number of brain regions engaging in a mode) and duration (continuous time interval during which a mode appears).

Our results demonstrate the emergence of static and dynamic properties of neural activity at different timescales from networks of delay-coupled oscillators at 40 Hz. Given the higher dependence of simulated FC on the underlying structural connectivity, we suggest that mesoscale heterogeneities in neural circuitry may be critical for the emergence of parallel cross-modal functional networks and should be accounted for in future modelling endeavours.

## Highlights

• We explore multimodal activity generated by two different large-scale generative models.
• Relevant dynamics across modalities emerge from delay-coupled gamma-band oscillators.
• Local E-I balance supports the emergence of spatiotemporal network dynamics.
• The omission of conduction delays dramatically decreases model performance.
• The connectome imposes model-specific constraints on functional connectivity.

## 1 Introduction

The art of large-scale modelling has been in practice since the 1940s (McCulloch and Pitts, 1943; Shimbel and Rapoport, 1948; Uttley and Matthews, 1955). However, with the progression of cutting-edge technology and advanced neuroimaging techniques, an unprecedented computational power and spatiotemporal resolution of data has been achieved, leading to the establishment of a methodological framework (Coombes, 2005; Deco et al., 2008; Honey et al., 2007). The purpose of large-scale modelling is to provide a parsimonious and precise explanation of data features, striking a balance between complexity and realism. It takes a biophysical approach to investigate how the interaction between structural connectivity and local dynamics gives rise to distinctive spatiotemporal oscillatory patterns.

Thus far, the most precise approximation of the structural organisation of the brain – the “connectome” – is represented by inter-regional connectivity patterns between anatomically defined brain regions. Long axonal projections mediate long-range interactions between distant neuronal ensembles, which can be non-invasively and in-vivo registered using diffusion MRI (Conturo et al., 1999; Hagmann et al., 2008; Sporns et al., 2005). The need for such comprehensive mapping has motivated researchers to make the structural description more and more detailed, aiming to depict brain structure at multiple levels and across different species (Alexander et al., 2007; Glasser et al., 2016; Hagmann et al., 2008; Ranzenberger and Snyder, 2022; Zalesky et al., 2010). This detailed structural mapping will serve to delineate the space of possibilities in which nodes and their interactions can be modelled as a network.

Models of brain oscillations aim to capture the relationship between synchronisation mechanisms and collective behaviour, as well as their reliance on coupling strength. Computational models of dynamical systems - such as the models of coupled phase (Kuramoto, 1975), limit cycle (Stuart Landau (Sreenivasan et al., 1987)), and chaotic (Rössler, 1976) oscillators, have been increasingly employed to study the evolving network dynamics emerging from the structural framework (Cabral et al., 2022, 2011; Cofré et al., 2020; Deco et al., 2008). In 1975, Kuramoto presented a reduced-order model that characterises the within-limit-cycle behaviour of nodes, representing the activity of each oscillator in terms of its circular phase (Bick et al., 2020; Kuramoto, 1975; Park and Lefebvre, 2020). Moving beyond the limit cycle, Andronov and colleagues proposed models that include both phase and amplitude modulation (Andronov et al., 1966). amongst those, the Stuart-Landau equation has been used to describe the appearance of an oscillatory mean field from a noisy dynamical unit (Pikovsky et al., 2003; Pikovsky and Rosenblum, 2015).

Governed by the same underlying principles, neural mass models (NMMs) have facilitated valuable insights into meso‑ and macroscopic dynamics of excitatory and inhibitory neuronal populations, with the aim of achieving neurobiological realism at a different scale (Beurle and Matthews, 1956; Wilson and Cowan, 1972). Contrasting with single oscillator models, NMMs depict each brain region as a group of interacting neurons whose oscillatory dynamics can be explained by their mutual interaction. In both cases, by finely tuning the model parameters, the system dynamics undergo a phase transition from a noisy to an ordered state. By coupling an ensemble of oscillators/NMMs, the simulated local activity depends not only on the intrinsic node dynamics in the presence of stochastic perturbations, but also on the interactions with other elements in the network (Breakspear, 2017).

A wealth of research shows that the neocortex maintains a so-called E-I balance - a delicate equilibrium between excitatory and inhibitory activity (Dehghani et al., 2016; Froemke et al., 2007; Sprekeler, 2017; Tao and Poo, 2005; Xue et al., 2014). This balance is crucial for optimal cortical function (Amil and Verschure, 2021; Litwin-Kumar and Doiron, 2014; Mariño et al., 2005; Páscoa Dos Santos and Verschure, 2021; Puigbò et al., 2018; Rubin et al., 2017; van Vreeswijk and Sompolinsky, 1996; Vogels et al., 2011; Wehr and Zador, 2003). Importantly, it is sustained through homoeostatic plasticity mechanisms that adjust the strength of synapses onto pyramidal neurons to stabilise firing rates (Ma et al., 2019; Turrigiano, 2011; Turrigiano et al., 1998; Vogels et al., 2011). One hypothesis is that inhibitory synaptic plasticity (ISP) plays a key role in balancing E-I inputs and stabilising firing rates whenever the E-I balance is disrupted by perturbations at the level of incoming excitation. By dynamically adapting synaptic weights (Landau et al., 2016; Litwin-Kumar and Doiron, 2012), ISP regulates local activity and generates more realistic functional patterns, as demonstrated in MEG (Abeysuriya et al., 2018) and fMRI models (Hellyer et al., 2016; Rocha et al., 2018; Vattikonda et al., 2016). Such modulation is crucial for the re-emergence of such patterns following substantial damage to underlying structural networks (Páscoa dos Santos et al., 2022).

Large-scale brain network models can not only accurately represent empirical data but also reveal structural-functional, and subsequent static-dynamic, relationships. Structural and functional neuroimaging techniques allow us to model anatomical connections and statistical interactions between brain regions (Friston, 1994). To explore the complex interplay between structural and functional connectivity, different modelling approaches have been used, ranging from biophysical (Breakspear, 2017; Deco et al., 2009, 2014; Honey et al., 2007; Pinotsis et al., 2012; Sanz Leon et al., 2013) to statistical methods (i.e., (Raj et al., 2020), see (Raj et al., 2022) for a comprehensive review). They share the notion of high-order neural phenomena going beyond the local geometrical clustering but also illustrate how the interplay between local dynamics and the large-scale anatomical framework gives rise to resting-state brain activity (Cabral et al., 2011; Deco et al., 2013, 2014).

Although functional connections between brain regions can exist even in the absence of structural connections (Hermundstad et al., 2014; Honey et al., 2010), they are still constrained by the brain's anatomical framework (Friston, 1997; Hutchison et al., 2013). Indeed, on slow time scales FC indirectly reflects the underlying SC (Honey et al., 2010). In the context of modelling, it has been observed that when the brain is close to a phase transition – a state where the system is highly sensitive to minor changes, and small perturbation can lead to significant effects – the statistical dependence between different brain regions is strongly influenced by the underlying anatomy of the network, the system is highly sensitive to small changes. This demonstrates a dynamic balance between integration and segregation, as well as an enhanced ability for spontaneous reconfiguration (Schirner et al., 2022). The concepts of bifurcation and phase transitions extend to the study of criticality, which refers to the dynamical regime of networks near the bifurcation point. Criticality has been measured extensively in brain dynamics (Cocchi et al., 2017) and has been shown to optimize functions such as information storage and transmission (Beggs and Timme, 2012). Recent findings also suggest that the brain homeostatically regulates local dynamics to maintain criticality (Ma et al., 2019).

This suggests that the optimal working point, linking function to structure, is at the edge of criticality (Cocchi et al., 2017). While this investigation has been well-established in fMRI, it has only recently been applied to electromagnetic data such as MEG/EEG (Cabral et al., 2022; Deco et al., 2017; Rabuffo et al., 2021; Roberts et al., 2019).

Moreover, the dynamic nature of functional connectivity has gained increasing attention due to its rich and transient reconfiguration over time (Cabral et al., 2017a; Deco et al., 2017; Hutchison et al., 2013), with changes in this dynamic connectivity reflecting cognitive or neurological dysfunction (Bonkhoff et al., 2021; Cabral et al., 2017b; Filippi et al., 2019; Lombardo et al., 2020; Polverino et al., 2022). While large-scale models exist for explaining the possible mechanisms behind the transient motifs of metabolic signals, such as synchronisation, spontaneous oscillatory activity, E-I neurons interaction, myelination and network topology (Deco et al., 2017, 2021; Vohryzek et al., 2020), few attempts have been made in the realm of electrophysiological data (Cabral et al., 2022, 2014; Deco et al., 2017). Various models have attempted to elucidate the mechanisms underlying each modality, such as (Glomb et al., 2022) for EEG, (Abeysuriya et al., 2018; Cabral et al., 2022; Hadida et al., 2018; Raj et al., 2020; Tewarie et al., 2019) for MEG, (Atasoy et al., 2016; Cabral et al., 2011; Deco et al., 2009, 2021; Honey et al., 2007; Roberts et al., 2019) for fMRI. However, the characterization of features detected across modalities by large-scale modelling approaches has only recently been initiated (Rabuffo et al., 2021). In particular, Rabuffo et al. (2021) demonstrated how neuronal cascades can be a major determinant of spontaneous fluctuations in brain dynamics captured with simultaneous EEG and fMRI. Nonetheless, the relationship between large-scale oscillations and their organisation across scales is yet to be thoroughly explored.

In this work, we apply a multi-modal and multi-model approach to recover the underlying neurodynamical genesis of neuroimaging signals. We aim to contribute to the broad repertoire of generative models by proposing a comparative analysis between two large-scale models, identifying advantages and limitations, and testing their applicability in disclosing the network properties of haemodynamic and electrophysiological brain activity. This paper starts with a concise exposition of the theoretical underpinnings of generative modelling and proceeds to a description of complementary modelling techniques applied to empirical data. These analyses provide the basis for a comparative evaluation of different modelling strategies, thereby facilitating the identification of their key functional forms.

## 2 Methods

### 2.1 Phase-amplitude model: stuart-landau

The Stuart-Landau (SL) equation (Eq. (1)) is the canonical form for describing the behaviour of a nonlinear oscillating system near an Andronov-Hopf bifurcation (Andronov et al., 1966; Cocchi et al., 2017). It describes systems that have a static fixed point but respond to perturbation (i.e., noise, impulse, specific waveform) with an oscillation, which may be damped or self-sustained depending on the operating point of the system with respect to the bifurcation (Supplementary Material (SM), Section I, Figure S1).

Our analysis is based on a system of *N* = 78 SL oscillators coupled in the connectome, considering both the connectivity strength, $C_{np}$, and the conduction delays, $\tau_{np}$, between each pair of brain areas n and p. The conduction delays are defined in proportion to the fibre lengths between brain areas, assuming a homogenous conduction speed v, such that $\tau_{np}$ $=D_{np}/v$, where $D_{np}$ is the real fibre length detected between brain areas n and p. To simulate how the activity in node n is affected by the behaviour of all other nodes (p∈N∧p≠n), we describe the interaction between nodes in the form:

(1) $$\frac{dZ_{n}}{dt}=Z_{n}\left[a+i\omega -\left|Z_{n}^{2}\right|\right]+K\sum_{p\neq n}^{N}C_{np}\left[Z_{p}\left(t-\tau_{np}\right)-Z_{n}\left(t\right)\right]+\beta \eta_{1}+i\beta \eta_{2}$$

where the complex variable $Z_{n}\left(t\right)\;$describes the state of the $n^{th}$ oscillator at time t.

The first term in Eq. (1) describes the intrinsic dynamics of each unit that is the natural excitability of neuronal assemblies, where $\omega =2\pi *f_{f}$ is the angular frequency, with $f_{f}$ as the fundamental frequency. As in (Cabral et al., 2022), we set all nodes with identical natural frequency $\omega_{0}=2\pi *40Hz$, representing the ability of a neural mass to engage in gamma-frequency oscillations.

The parameter a determines the position of each unit with respect to the limit cycle. For a>0, a stable limit cycle appears via a superciritical Hopf bifurcation, while when a<0there is only a stable fixed point at the origin $Z_{n}=0$, so the bifurcation point is at a = 0. Importantly, if a is negative but sufficiently close to the bifurcation, the system is still weakly attracted to the limit cycle and damped oscillations emerge in response to external input, with a decay time scaled by a.

The second term represents the total input received from other brain areas, scaled by parameter K, which sets the strength of all network interactions with respect to the intrinsic node dynamics. Because we focus on the nonlinear phenomena introduced by time delays, we model the node-to-node interactions using a particular *linear diffusive coupling*, as the simplest approximation of the general coupling function, considering *delayed* interactions. The last term of Eq. (1) represents the real and imaginary part of uncorrelated white noise, where $\eta_{1}$ and $\eta_{2}$ are independently drawn from a Gaussian distribution with mean zero and standard deviation β=0.001. The parameters chosen in this study are presented in Table 1. For a detailed exploration and dynamical analysis of SL model see (Cabral et al., 2022; Choe et al., 2010; Powanwe and Longtin, 2021).

### 2.2 Neural mass model

Neural mass-models are mean-field approaches that function under the assumption that the activity of a discrete population of neurons, or neural mass, can be abstracted to its mean, or any other statistic of interest, at a given time (Breakspear, 2017). In our work, to simulate activity of parcellated cortical regions, we make use of one of such approaches: the Wilson-Cowan model of coupled excitatory and inhibitory populations (Wilson and Cowan, 1972). The Wilson-Cowan model describes the firing-rate dynamics of two recurrently connected populations of excitatory ($r^{E}$) and inhibitory ($r^{I}$) neurons, being, for this reason, ideal to represent local excitatory-inhibitory balance (Abeysuriya et al., 2018). The dynamics of these two variables can then be described as:

(2) $$\begin{array}{ccc} \tau_{E}\frac{dr_{n}^{E}\left(t\right)}{\mathrm{dt}} & = & -r_{n}^{E}\left(t\right)+F\\ & & \times \left[c_{\mathrm{EE}}r_{n}^{E}\left(t\right)-c_{\mathrm{EI},\;n}\left(t\right)r_{n}^{I}\left(t\right)+K\sum_{p=1}^{N}C_{\mathrm{np}}r_{p}^{E}\left(t-\tau_{\mathrm{np}}\right)+\xi \left(t\right)+P\right] \end{array}$$

(3) $$\tau_{I}\frac{dr_{n}^{I}\left(t\right)}{dt}=-r_{n}^{I}\left(t\right)+F\;\left[c_{IE}r_{n}^{E}\left(t\right)+\xi \left(t\right)\right]$$

where $\tau_{E}$ and $\tau_{I}$ represent the characteristic time constants of the excitatory and inhibitory populations, respectively, $c_{XY}$ describes the coupling from population y to x (e.g., $c_{EI}$ represents the inhibitory to excitatory coupling) andK is a scaling factor for structural connectivity, hereby referred to as global coupling. $C_{np}$ represents the structural connection (through white-matter tracts) between nodes n and p and is based on human structural connectivity data derived from diffusion tensor imaging (see *Structural Connectivity*, Methods). In turn, $\tau_{np}$, describes the conduction delay between nodes n and p and is calculated by dividing empirically derived tract lengths by a given conduction speed. Notably, these long-range connections are only implemented between local excitatory populations, in accordance with the evidence that long-range connections in the human cortex are predominantly excitatory (Tremblay et al., 2016) and in line with the state-of-the-art in large-scale modelling (Abeysuriya et al., 2018). As in Abeysuriya et al. (2018), we add a parameter P to the description of $r^{E}$, regulating the excitability of excitatory populations (SM, Section I, Figure S1).

To describe the response of neural masses to external input, we use the function F(x). Shortly, F(x) can be roughly equated to the F-I curve of a given population of neurons, and is described as:

(4) $$F\left(x\right)=\;\frac{1}{1+e^{-\frac{x-\mu }{\sigma }}},$$

where μ represents the input level at which the neural mass reaches half of its maximum response and can be understood as regulating its excitability, and σ is the approximate slope of the function at that point, equating to the sensitivity of the neural mass to external input. In addition, both excitatory and inhibitory populations receive uncorrelated additive noise, drawn at each time point from a Gaussian distribution with mean 0 and standard deviation 0.01. For the chosen parameters describing local interactions ($c_{XY}$) ((Abeysuriya et al., 2018), Table 1), the uncoupled Wilson-Cowan node behaves as a Hopf-Bifurcation between a low-activity steady-state and a limit-cycle (Wilson and Cowan, 1972). Therefore, if the system is close to the bifurcation point, it will transiently exhibit noise-driven oscillations. While the bifurcation point is determined by $\frac{\tau_{E}}{\tau_{I}}$, the intrinsic frequency of oscillation depends, instead, on $\tau_{E}\tau_{I}$. Since cortical networks are thought to generate intrinsic gamma oscillations through the recurrent interaction between pyramidal cells and fast-spiking inhibitory interneurons (Buzsáki, 2006), we chose $\tau_{E}$ and $\tau_{I}$ so that the characteristic frequency of isolated neural masses is within the gamma range (∼40 Hz) (see SM, Section I, Figure S2). In addition, to control the level of input necessary for the phase transition between stable activity and the limit cycle to occur, we regulate the excitability of the neural masses through the parameters μ and P. Here, we chose parameters so that an isolated neural mass, with no external input, is in the subcritical regime but sufficiently close to the critical bifurcation point, so that damped oscillations emerge when receiving input from other nodes.

### 2.3 Homoeostatic plasticity

To study the effect of balancing excitation and inhibition at the level of single Wilson-Cowan nodes, we implemented a homoeostatic mechanism known as synaptic scaling of inhibitory synapses (Maffei and Turrigiano, 2008; Vogels et al., 2011). This type of approach has been previously implemented in large-scale models of the human cortex (Abeysuriya et al., 2018) and inhibitory synaptic scaling has been shown to play an essential role in cortical function and homoeostasis (Ma et al., 2019). Therefore, we implemented homoeostatic plasticity to adjust local inhibitory weights so that excitatory activity ($r^{E}$) is corrected towards a given target firing rate (ρ). Therefore, the dynamics of local inhibitory couplings $c_{EI,i}$ can be described by the following equation, following (Vogels et al., 2011):

(5) $$\tau_{homeo}\frac{dc_{EI,i}}{dt}=r_{i}^{I}\left(r_{i}^{E}-\rho \right),\;$$

where $\tau_{homeo}$ is the time constant of plasticity. In the cortex, the homoeostatic mechanisms that are responsible for the maintenance of E-I balance are known to operate in slow timescales, often hours to days (Turrigiano, 2011). However, to ensure the computational tractability of our simulations, we chose $\tau_{homeo}=2.5s$. This choice is unlikely to affect our results significantly, since the influence of $\tau_{homeo}$ in our system is in determining how quickly local inhibitory weights evolve towards their steady state. In fact, if homoeostatic plasticity is sufficiently slow to be decoupled from fast dynamics of intrinsic oscillations, $c_{EI}$ will reach nearly the same steady state, independently of the time constant (SM, Section I, Figure S3). We also ran simulations not considering homoeostatic plasticity to pursue a comparative analysis (SM, Section I, Figure S4).

### 2.4 Models parameters

Table 1

**Table 1:** Table of parameters, values and descriptions for both large-scale modes. a. Wilson-Cowan model's parameters. b. Stuart Landau model's parameters.

a. Wilson-Cowan		
Parameter	Value	Description
*K*	[0.1, 14]	Global coupling, scaling factor of structural connectivity
Mean Delay	[0, 15]	(ms) Mean conduction delay across non-zero connections
$\tau_{E}$	2.5	(ms) Time constant of excitatory population
$\tau_{I}$	5	(ms) Time constant of inhibitory population
$c_{EE}$	3.5	Recurrent coupling of excitatory populations
$c_{IE}$	3.75	Coupling from excitatory to inhibitory populations
*P*	0.31	Adjusts excitability of excitatory population
μ	1	Firing threshold of activation function F(x)
σ	0.25	Sensitivity of activation function F(x)
$\tau_{homeo}$	2500	(ms) Time constant of homoeostatic plasticity
ρ	0.22	Target firing rate of homoeostatic plasticity
ξ	N (0,0.01)	Additive gaussian noise

b. Stuart-Landau		
Parameter	Value	Description

*K*	[4.0, 2000]	Global coupling, scaling factor of structural connectivity
Mean Delay	[0, 15]	(ms) Mean conduction delay across non-zero connections
*a*	−5	Bifurcation parameter
ω	2π*40	(radians) Intrinsic frequency of oscillation
β	0.001	Standard deviation of additive gaussian noise

### 2.5 Hemodynamic model

To extract a blood-oxygenation-level-dependant (BOLD) signal equivalent from our simulations, we make use of a forward hemodynamic model (Friston et al., 2000), that incorporates the Balloon-Windkessel model (Friston et al., 2003). In short, hemodynamic models describe how population firing rates (a proxy for neuronal activity) influence the vasculature, which in turn affects blood flow, inducing changes in blood vessel volume and deoxyhemoglobin content, which underlie BOLD signals. In our work, we chose to use the activity from the excitatory populations ($r^{E}$) only as the input of the Balloon-Windkessel model. This choice is unlikely to influence the final results, given the similarity between $r^{E}$ and $r^{I}$ in the Wilson-Cowan model (SM, Section I, Figure S1). All of the parameters were taken from (Friston et al., 2003). In addition, we down-sample the simulated BOLD signals to a period of 0.72 s to equate the sampling frequency of the empirical data used in this work (see *fMRI*, Methods).

### 2.6 Model optimisation

We performed model optimisation by treating the global coupling (K) and mean delay (mean tract length divided by conduction velocity) as free parameters for both models. For the Wilson-Cowan model, we fixed the target firing rate (ρ) of homoeostatic plasticity at 0.22 (a.u.). We also ran simulations for different ρ values (see SM, Section I, Figure S7). We performed a grid search for both models over the mentioned free parameters, with 25 logarithmically spaced values of K and 16 values of mean delays in steps of 1 ms. Parameter ranges can be consulted in Table 1. For the SL model, simulations with the two highest values of K explored led to instability and results are, therefore, not presented.

For the WC simulations, due to the dynamics of homoeostatic plasticity, there was a need to ensure that local inhibitory weights reached a stable or quasi-stable steady state before activity was recorded. Therefore, during simulations, we record $c_{EI\;}$weights every 10 s, enough to capture their slow dynamics. We then monitor the evolution of $c_{EI\;}$ and allow simulations to run for either 500 min of simulation time or until local weights converged to a steady state for all network nodes, evaluated via the condition described in supplementary material (SM, Section I, Figure S4). After ensuring that $c_{EI\;}$reached a steady state, we disable plasticity and record 20 min of model activity. Although the slow dynamics of E-I homoeostasis prevent it from interacting with the fast dynamics of neural activity, we follow this procedure similarly to previous approaches (Abeysuriya et al., 2018; Hellyer et al., 2016). Regarding the SL model, we run and record 20 min of simulation. We ran simulations with an integration time step of 0.2 milliseconds.

For both models, after obtaining 20 min of simulations, we passed the simulated activity through a haemodynamic model to obtain a synthetic BOLD signal and remove the first and last 2.5 s to avoid boundary effects, thus obtaining 15 min of BOLD signal timeseries (Friston et al., 2000). To represent MEG signals, we considered node activity, for the SL model, and activity from excitatory populations, for the WC model), downsampled to 250 Hz. We compared simulated and empirical FC matrices (see *Data and Model Analysis*, Methods) through the correlation coefficient between their upper triangular parts, and FCD and MOM size (see *Data and Model Analysis*, Methods) through the Kolmogorov-Smirnov (KS) distance between simulated and empirical distributions (Lopes et al., 2007). To identify an optimal working point for each model and each measured modality (BOLD, MEG theta, MEG alpha and MEG beta – see below for details), we iterate over a range of thresholds for FC correlation ($cc\geq th_{FC\;}$), FCD KS distance ($KS\leq th_{FCD\;}$) and MOM size KS distance ($KS\leq th_{MOM_{size}}$) and identify the maximum value of $th_{FC}-th_{FCD}-th_{MOM_{size}}$ for which the three conditions can be satisfied by at least one point in the parameter space (see SM, Section III, Figure S9). We then define our model's working point, for each modality, as the combination of parameters (global coupling and mean delay) that satisfies those specific thresholds. Since we primarily focus on the representation of relevant FC patterns, we impose 0.4 as the minimum $th_{FC\;}$.

### 2.7 Data collection and processing

#### 2.7.1 Ethics statement

All human data used in this study is from the public repository of the Human connectome Project (HCP) (https://www.humanconnectome.org), which is distributed in compliance with international ethical guidelines.

#### 2.7.2 Structural connectivity

The *NxN* matrices of structural connectivity, C, and distances, D, used in the brain network model were computed from diffusion spectrum and T2-weighted Magnetic Resonance Imaging (MRI) data obtained from 32 healthy participants scanned at the Massachusetts General Hospital centre for the Human connectome Project (http://www.humanconnectome.org/).

Briefly, the data were processed using a generalised q-sampling imaging algorithm implemented in DSI Studio (http://dsi-studio.labsolver.org). A white-matter masque, derived from the segmentation of the T2-weighted anatomical images, was used to co-register the images to the b0 image of the diffusion data using the SPM12 toolbox (https://www.fil.ion.ucl.ac.uk/spm/software/spm12/). In each participant, 200,000 fibres were sampled within the white-matter masque. Fibres were transformed into Montreal Neurological Institute (MNI) space using Lead-DBS (Horn and Blankenburg, 2016) .

The connectivity matrix C was obtained by counting the number of fibres detected between each pair of *N* *=* *78* brain areas defined in the Automated Anatomical Labelling (AAL) parcellation scheme. Similarly, the distance matrix D was obtained by computing the mean length of all fibres detected between each pair of *N* *=* *78* cortical brain areas.

#### 2.7.3 fMRI

Empirical fMRI data from healthy subjects was obtained from the public database of the Human Connectome Project (HCP), WU-Minn Consortium (Principal Investigators: David Van Essen and Kamil Ugurbil; 1U54MH091657) funded by the 16 NIH Institutes and Centers that support the NIH Blueprint for Neuroscience Research, and by the McDonnell centre for Systems Neuroscience at Washington University (Van Essen et al., 2013). More specifically, this data was obtained from 99 unrelated subjects (mean age 29.5, 55% females). Each subject underwent four resting-state fMRI sessions of around 14.5 min on a 3-T connectome Skyra scanner (Siemens) with the following parameters: TR = 0.72 s, echo time = 33.1 ms, field of view = 208×180 mm, flip angle = 52º, multiband factor = 8, echo time = 33.1 with 2 × 2 × 2 isotropic voxels with 72 slices and alternated LR/RL phase encoding. For further details, on the standard processing pipeline for HCP data, please consult (Glasser et al., 2016) and https://www.humanconnectome.org/study/hcp-young-adult/data-releases. In this work, we use the data from the first session of the first day of scanning only.

We further parcellate voxel-based data into 90 anatomically segregated cortical and subcortical regions, excluding the cerebellum, using the anatomic Automatic labeling (AAL) atlas. Given that we focus on cortical dynamics, we exclude the 12 subcortical regions, and perform a voxel-wise average of BOLD signals associated with each of the remaining 78 cortical regions, reducing the size of our data to 78 areas x 1200 TR timeseries.

#### 2.7.4 MEG

Pre-processed sensor level MEG data, along with a defaced structural MRI and the appropriate affine transformation matrix mapping between the MRI and MEG spaces were downloaded from the HCP data repository (Wu-Minn HCP 1200 Subjects Data Release). Each of the 89 subjects underwent 6-minute resting state scans (where they were instructed to lie still and keep their eyes open), giving a total of 267 datasets. Full details of the pre-processing steps performed by the HCP team can be found in the HCP manual (https://www.humanconnectome.org/storage/app/media/documentation/s1200/HCP_S1200_Release_Reference_Manual.pdf).

All processing steps were carried out in FieldTrip (Oostenveld et al., 2011) in MATLAB 2021b. The anatomical MRI was linearly transformed from the native MRI space to the MEG scanner space, before being segmented into grey matter, white matter, and cerebral spinal fluid. This segmentation informed the construction of a Nolte single shell head model (Nolte, 2003). A common template array of voxels (isotopically distributed on a grid with 8 mm separation, confined to lie within the brain) was non-linearly aligned from MNI space to each of the individual subject's anatomical images using SPM8’s “old normalise” function (Ashburner and Friston, 2005). This meant that there was a “standard” source model used in the pipeline, with one-to-one correspondence between sources across subjects.

Nearest-neighbour interpolations between this template grid and the atlases that we used in this study were applied, facilitating the parcellation of voxels into anatomically defined brain regions. A volumetric lead field matrix was calculated for each of the voxel locations. We collapsed the rank of the lead field for each voxel from three to two by a singular value decomposition (SVD), thus eliminating any sensitivity to the weakly contributing radial component of the lead field (Ahlfors et al., 2010; Hämäläinen et al., 1993).

The pre-processed sensor level MEG recordings were further band-pass filtered between 1 and 45 Hz and downsampled to 250 Hz. These data were used to construct a covariance matrix for the construction of linearly constrained minimum variance (LCMV) beamformer weights (Van Veen et al., 1997). This matrix was regularised by adding 1% of the average eigenvalue to the diagonal to improve numerical stability and boost the reconstruction accuracy of the estimated time series (Van Veen et al., 1997). At each voxel location, a separate SVD was run on the 3-dimensional vector time series to extract the optimal lead field orientation in order to maximise the SNR of beamformer weights (Sekihara et al., 2004), thus collapsing the 3 element timeseries to a single time series for each voxel.

### 2.8 Data and model analysis

#### 2.8.1 fMRI FC

To compute functional connectivity (FC) from BOLD signals, both empirical and simulated, we calculate pair-wise correlations between all individual timeseries from each of the 78 cortical areas of the AAL atlas, using the Pearson's correlation coefficient. We then averaged FC over the 99 subject-specific correlation matrices to obtain a 78×78 empirical FC matrix, against which simulated FC matrices can be compared.

#### 2.8.2 MEG FC

Upon obtaining estimates for the neural source currents, data were parcellated into nodes pertaining to each atlas. The first principal component was extracted from all voxels within each ROI. Data were then corrected for spurious correlations arising from source leakage between brain regions by means of symmetric orthogonalization (Colclough et al., 2015). After bandpass filtering the data, we took the analytical signal of the Hilbert envelope for all brain regions. Hilbert envelopes were then low-pass filtered above 0.5 Hz and downsampled to 5 Hz, as in (Portoles et al., 2022). Whole brain functional connectivity networks were derived by calculating the pair-wise Pearson correlation between filtered and downsampled envelopes of each network node. Finally, we calculated the average amplitude envelope FC matrix over all subjects and sessions. See SM, section V, for further details on amplitude envelope correlation, MEG source leakage correction and beamforming methods. For both simulated and empirical data, we used a total of 300 s of signal to compute FC. Simulated signals were not leakage corrected, since simulated data does not have source leakage (Portoles et al., 2022).

#### 2.8.3 fMRI functional connectivity dynamics

While research has mostly focused of the static properties of FC, recent results show that functional connectivity exhibits complex spatiotemporal dynamics, with the transient reinstatement of connectivity states (Deco et al., 2017). Here, to evaluate functional connectivity dynamics (FCD), we make use of the method presented in (Abeysuriya et al., 2018; Deco et al., 2021, 2017). We first split data in N_T_ windows of 80 samples (∼1 min) with 80% overlap and compute FC within each window following the method described in the previous section. Then, for all pairs of windows, we compute the Pearson's correlation between the upper triangle of their respective FC matrices. We thereby obtain an N_T_ x N_T_ matrix containing all pairwise correlations between the windowed FC matrices. We then concatenate the values in FCD matrices across subjects to obtain an empirical distribution, against which we compare FCD distributions from each simulation.

#### 2.8.4 MEG functional connectivity dynamics

To calculate FCD from MEG signals, we follow a similar method to the one described in the previous section, with slight modifications, given the nature of MEG signals. Firstly, we filter the MEG data at three frequencies (theta: 4–8 Hz, alpha: 8–13 Hz, beta: 13–30 Hz) and compute the frequency-specific amplitude envelopes, as described in the analysis section of the methods. Next, we apply a low-pass filter above 0.5 Hz to the amplitude envelopes and down-sample the filtered envelopes to 5 Hz, using the same methods as (Portoles et al., 2022). The resulting filtered and downsampled timeseries are divided into windows of 30 s with 80% overlap. It is not yet clear what the appropriate window size is for the calculation of FCD in MEG signals, due to the diverse timescales of the emergence of spatiotemporal MEG patterns (Liuzzi et al., 2019). For this reason, we opted for a more conservative window size of 30 s, similar to (Portoles et al., 2022). Finally, we use the same procedure as for BOLD signals, for each frequency band, to calculate FCD. We then obtain frequency-specific distributions of FCD from the empirical MEG data, using a total of 300 s of signal to compute the FCD distributions, against which we compare FCD distributions from each simulation. As there is no source leakage in simulated data, simulated data was not orthogonalized.

#### 2.8.5 Metastable Oscillatory Modes

Previous results suggest that coupled oscillators with delayed interactions give rise to the emergence of metastable oscillatory modes (MOMs) (Cabral et al., 2022). These MOMs consist in transient moments of synchronisation between clusters of nodes in a network at frequencies that are lower than the intrinsic frequency of oscillation of uncoupled nodes.

To detect MOMs in both empirical and simulated fMRI and MEG data we first filter timeseries at the bands of interest (fMRI: 0.008–0.08 Hz, MEG theta: 4–8 Hz, MEG alpha: 8–13 Hz, MEG beta: 13–30 Hz). Then, we calculate the respective Hilbert envelopes by computing the absolute value of the Hilbert transform of timeseries from each individual area. Hilbert envelopes are then Z-scored (Z=(x−μ)/σ, where x is the Hilbert envelope, μ its mean and σ its standard deviation) and a threshold of 2 is applied for the detection of MOMs (Fig. 2). While the threshold is arbitrary, assuming that data is normally distributed, a value of 2 represents the threshold above which an incursion of the signal is distinct from noise with a significance level of *p*<0.05 (Hellyer et al., 2016). Different thresholds were tested, leading to the same qualitative results when comparing simulated and empirical data (SM, Section III, Figure S12).

While in the original approach MOMs were detected using a threshold derived from activity of models without delayed interactions (Cabral et al., 2022), we chose instead to threshold timeseries against their own standard-deviation. We followed this approach to compare the properties of MOMs from simulated and empirical results, since the original method does not allow for the detection of MOMs in empirical data. Similar methods have been applied to the detection of neural avalanches in MEG and MRI data (Hellyer et al., 2016; Sorrentino et al., 2021).

To quantify the properties of MOMs, similarly to (Cabral et al., 2022), we use of the following metrics:

1. Size: number of areas with amplitude higher than threshold at a given point in time
2. Duration: continuous time interval during which an amplitude timeseries is higher than threshold

Furthermore, MOM sizes and durations from empirical data were concatenated across subjects to compute empirical distributions against which simulated data can be compared.

#### 2.8.6 Modality-specific functional networks

To assess the ability of models to represent FC within relevant functional modules, or sub-networks, across modalities, we first detected such modules in empirical data. To do that, we detected modules from empirical FC averaged across subjects by using a clustering algorithm. In short, for a pre-determined number of clusters, we applied a k-means clustering 200 times on empirical FC and built an association matrix where each entry $A_{ij}$ represents the proportion runs in which nodes i and j were clustered together. We then applied k-means clustering again on the association matrix to detect modules or functional sub-networks. To choose the appropriate number of clusters for each signal modality we detected local minima in the cluster inertia (sum of square distances to cluster centroid) as a function of the number of clusters. Therefore, we obtained 6 networks for fMRI, 4 for MEG- θ, 5 for MEG-α and 5 for MEG-β (SM, Section III, Figure S14). To evaluate model performance in the representation of the previously obtained functional networks, we took FC within each sub-network for simulated and empirical data and computed the correlation coefficient between the upper triangular parts of both matrices. In addition, we performed the same analysis for between-network connectivity, by computing the correlation coefficient between simulated and empirical FC between nodes belonging to each pair of networks detected through the clustering algorithm defined previously.

## 3 Results

**Fig. 1:**
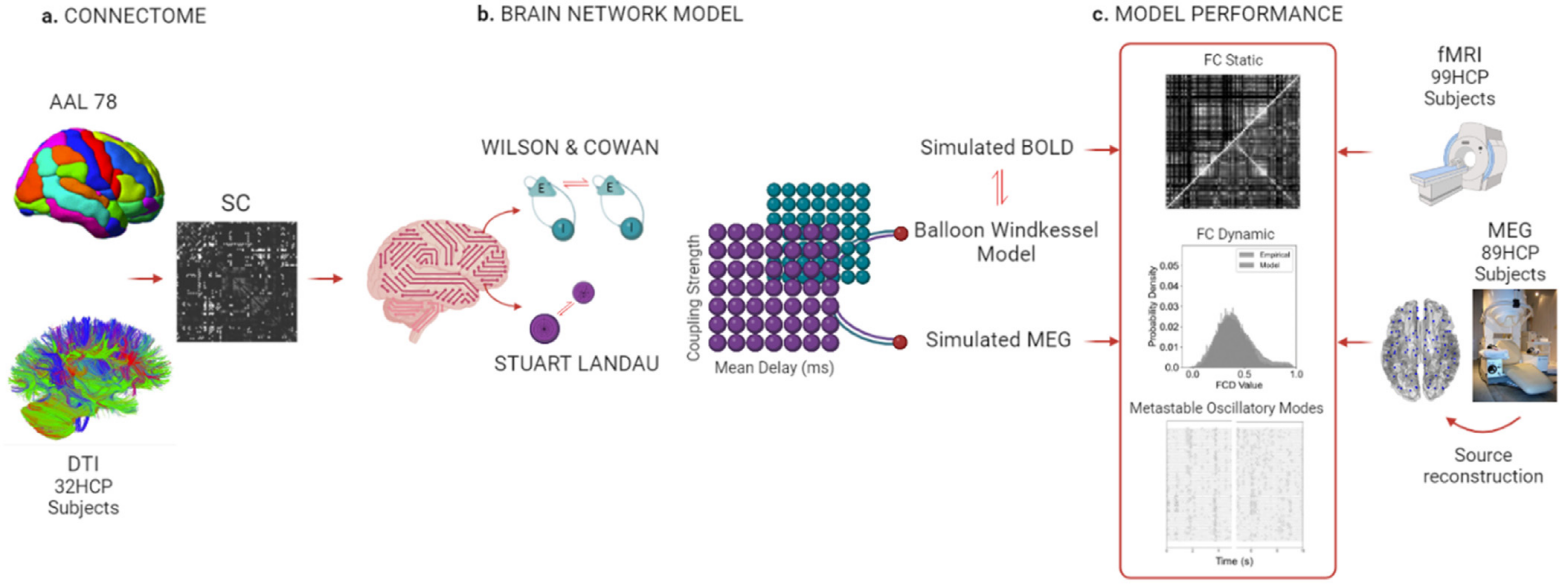
Pipeline overview. a. To build our structural connectivity (SC) we use averaged diffusion tensor imaging (DTI), generated by delineating the white matter fibres orientation of 32 healthy subjects and a cortical parcellation (AAL) for partitioning the cortex into 78 Region of Interests (ROIs). In the final SC graph, each ROI becomes a node and fibres become edges. b. We use this connectome to inform both phenomenological models. Both models are characterised by non-linear differential equations, whose parameters are tuned according to physiological plausibility to generate the oscillatory patterns observed empirically. Building on previous findings, the intrinsic frequency of all units is set at ω = 40 Hz and each unit is perturbed with uncorrelated white noise for both models (Cabral et al., 2022; Deco et al., 2009). In this study, we optimised two global parameters; namely, the coupling strength and the mean conduction delay, which were varied over specific ranges to best explain the empirical data features. For each combination of these two parameters, the models generate an oscillatory pattern. To create a BOLD signal from our simulations, a forward hemodynamic model is implemented, while we do not apply any additional steps to represent the simulated MEG signals. c. Both simulated and empirical signals follow the same pre-processing and analysis steps before being compared: for each combination of global parameters, we compute and compare the models’ and empirical functional connectivity, functional connectivity dynamics and properties of metastable oscillatory modes (see Methods for details).

To explore the spontaneous dynamics observed in resting- state fMRI and MEG data from healthy individuals, we used two generative brain network models of varying realism: the Stuart Landau (SL) model – based on a system of delayed coupled oscillators, and the extended Wilson and Cowan (WC) model – based on a system of coupled excitatory and inhibitory neural populations including delays and homoeostatic inhibitory plasticity. We investigate three key features of the brain: functional connectivity (FC), functional connectivity dynamics (FCD), and metastable oscillatory modes (MOM) size. Fig. 1 shows the overall pipeline.

**Fig. 2:**
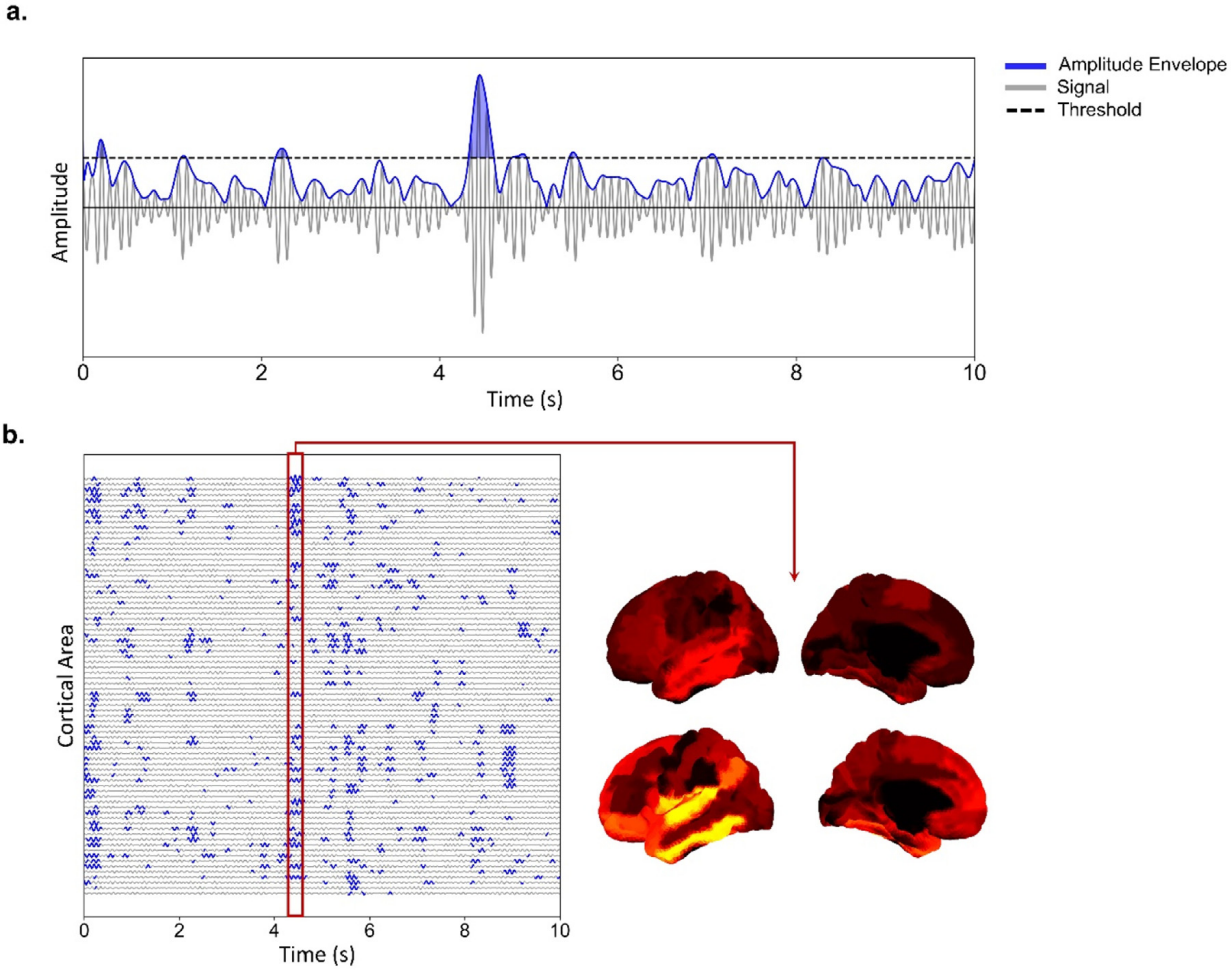
Capturing metastable oscillatory brain activity: a graphical representation. a. Identifying a metastable oscillatory mode within a brain signal: we first filter the timeseries for specific frequency bands, followed by calculating the amplitude envelope (Hilbert envelopes) by determining the absolute value of the Hilbert transform of timeseries from each individual area. Subsequently, the Hilbert envelopes are Z-scored, and a threshold of 2 is applied for detecting Metastable Oscillatory Modes (MOMs) – illustrated by the blue shaded area (refer to *Methods* for further details). b. An example of MOM across 78 cortical areas, organised in time and space. Each mode showcases a distinct topology, as depicted by the colour-coded representation in the bottom right corner.

FC refers to the synchronised activity between different regions of the brain. FCD, on the other hand, refers to changes in this connectivity over time, and can help to elucidate how these networks interact and evolve over different timescales. Finally, we also examine the size of MOMs, which are patterns of activity that persist for some time before transitioning into a different pattern (Cabral et al., 2022). To better understand MOMs, we provide a detailed figure that illustrates their definition and significance (Fig. 2).

### 3.1 Impact of parametrisation on models’ performance

**Fig. 3:**
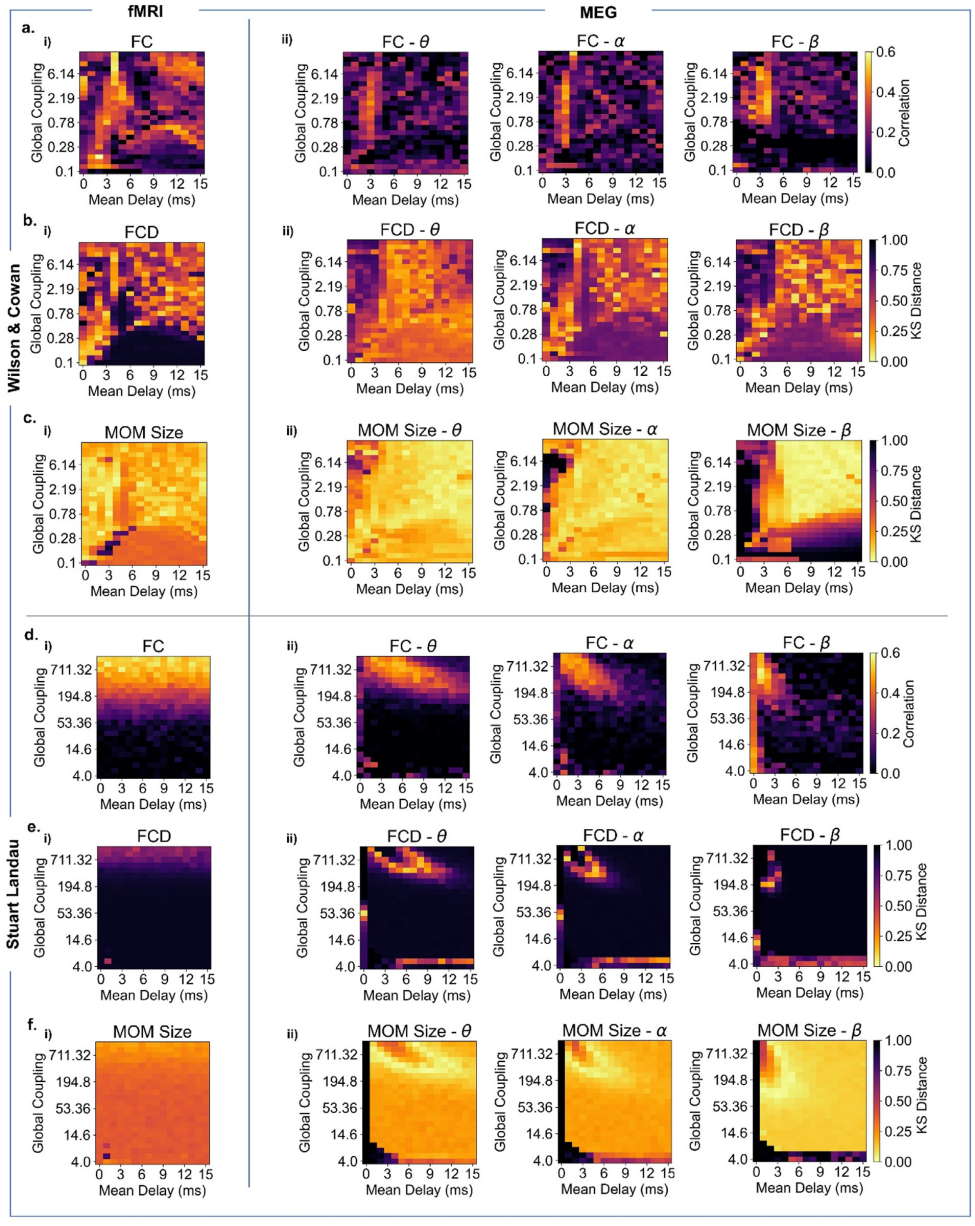
Unveiling frequency-specific connectivity and dynamic spatiotemporal patterns in model network parameter space. (a,d). Model performance in explaining empirical BOLD fMRI and MEG static connectivity measures for the WC model (a) and SL model (d). i) Pearson correlation between BOLD fMRI FC (averaged across 99 HCP participants) and simulated FC for each pair of parameters (Mean Delay and Global Coupling). ii) Pearson correlation between MEG Hilbert envelope FC (averaged across 89 HCP participants) and simulated Hilbert envelope FC for each pair of parameters, in theta [4–8 Hz] (left), alpha [8–13 Hz] (middle), beta [13–30 Hz] (right). (b,e) Model performance in representing empirical BOLD fMRI and MEG dynamical connectivity measures for the WC model (b) and SL model (e). i) Kolmogorov-Smirnov (KS) distance between empirical BOLD fMRI FCD histograms and simulated FCD histograms for each pair of parameters. ii) KS distance between empirical Hilbert envelope MEG FCD histograms and simulated Hilbert envelope FCD histograms for each pair of parameters, for theta (*left*), alpha (*middle*), beta (*right*). (c,f). Model performance in representing empirical BOLD fMRI and MEG MOM size – that is how many areas engage in a metastable mode for the WC model (c) and SL model (f). i)KS distance between empirical BOLD fMRI MOM size distribution and simulated MOM size distribution for each pair of parameters for WC and SL model. *ii)* Kolmogorov-Smirnov (KS) distance between empirical MEG MOM size distribution and simulated MOM size distribution for each pair of parameters, for theta (*left*), alpha (*middle*), beta (*right*) frequency bands.

We assessed the models’ performance in explaining FC, FCD, and MOM size (Fig. 3). Both models demonstrate comparable performance in representing FC for individual modalities, with the key difference being that, while the WC performance is most dependant on conduction delays, the SL parameter space is instead shaped by the presence of a delay-coupling interaction. In terms of FCD, the WC model performs well across all modalities, particularly BOLD, while the SL model exhibits limited capability. Regarding MOM size, both models perform reasonably well, although the SL model is not able to accurately mimic BOLD MOMs. Notably, delays played a significant role in both models, particularly for the SL model in representing MOMs accurately (Fig. 3f). A similar effect is also observed in the WC model, especially for higher frequency MOMs (Fig. 3c). Besides MOM size distributions, we also analysed model performance in approximating MOM durations, that is how long the network is engaged in a given MOM (SM, Section III, Figure S10). Both models can approximate the distribution of MOM durations across modalities. Importantly, empirical MOMs have a characteristic duration specific to each modality and progressively shortened for higher frequency bands.

Specifically, our study shows that delays impact signal features differently. In the WC model, delays have a limited fitting range, and there is a higher tolerance for global coupling due to homoeostatic plasticity. Conversely, the SL model exhibits an interaction between coupling and delays, with a wider delay range that narrows as frequency increases.

Interestingly, a region of parameter space in the WC model allows simulated haemodynamic patterns to approximate empirical patterns without delays (MD_WC_= 0 ms, 3 < K_WC_ < 5) (Figs. 3a-c, i), aligning with fMRI literature (Deco et al., 2017). However, it should be noted that when accounting for local dynamics with a higher intrinsic frequency (i.e., MEG), delays have been shown to induce much richer and more realistic dynamics (Cabral et al., 2014; Deco et al., 2017). This observation held for both models (Fig. 3a-c, ii).

While the WC model has a mechanism that can regulate local dynamics — and ensure brain areas are poised at a point where they can optimally respond to perturbations — the SL model relies solely on the interaction between coupling and delays to reproduce these dynamics. This suggests that the WC model's inclusion of E-I homoeostasis facilitated more efficient recruitment of local dynamics, allowing for better propagation of relevant spatiotemporal patterns of network activity, which most likely contributed to the model's improved performance in reproducing BOLD FCD. In addition, the contribution of local E-I balance, especially for the emergence of global dynamics, is highlighted by the fact that the WC model without plasticity does not achieve satisfactory performance in approximating empirical features. While FCD distributions can be matched in certain regions of the parameter space, especially for MEG, they do not co-occur with an accurate representation of FC. The most substantial effect of not accounting for E-I balance is observed for MOM size distributions, suggesting a particular relevance of local E-I balance for the occurrence of empirical-like oscillatory dynamics (SM, Section III, Figure S6).

Having demonstrated the presence of E-I balancing mechanism on network dynamics, we explore the effect of varying the target firing rate. This identifies the “target” (i.e., setpoint) homoeostatic mechanisms adapt towards. Generally, the target firing rate is a local parameter that plays a crucial role in allowing for a multiscale exploration of large-scale networks. By altering the WC model's target firing rate, we can reproduce network responses and the emergence of complex global dynamics from local interactions. In this work, we explore different target firing rates (ϱ= 0.07, 0.14, 0.28) (SM, Section III, Figure S7). Our results suggest that the lowest target firing rate (ϱ=0.07) is insufficient for fitting our features effectively. Increasing the rate to 0.14 approaches optimal performance (ϱ=0.22) without reaching it, and for ϱ=0.28, the goodness of fit decreases, particularly for MOM size distributions, but the model still fits FC and FCD.

### 3.2 Comparing models’ performance in simultaneously representing empirical features

**Fig. 4:**
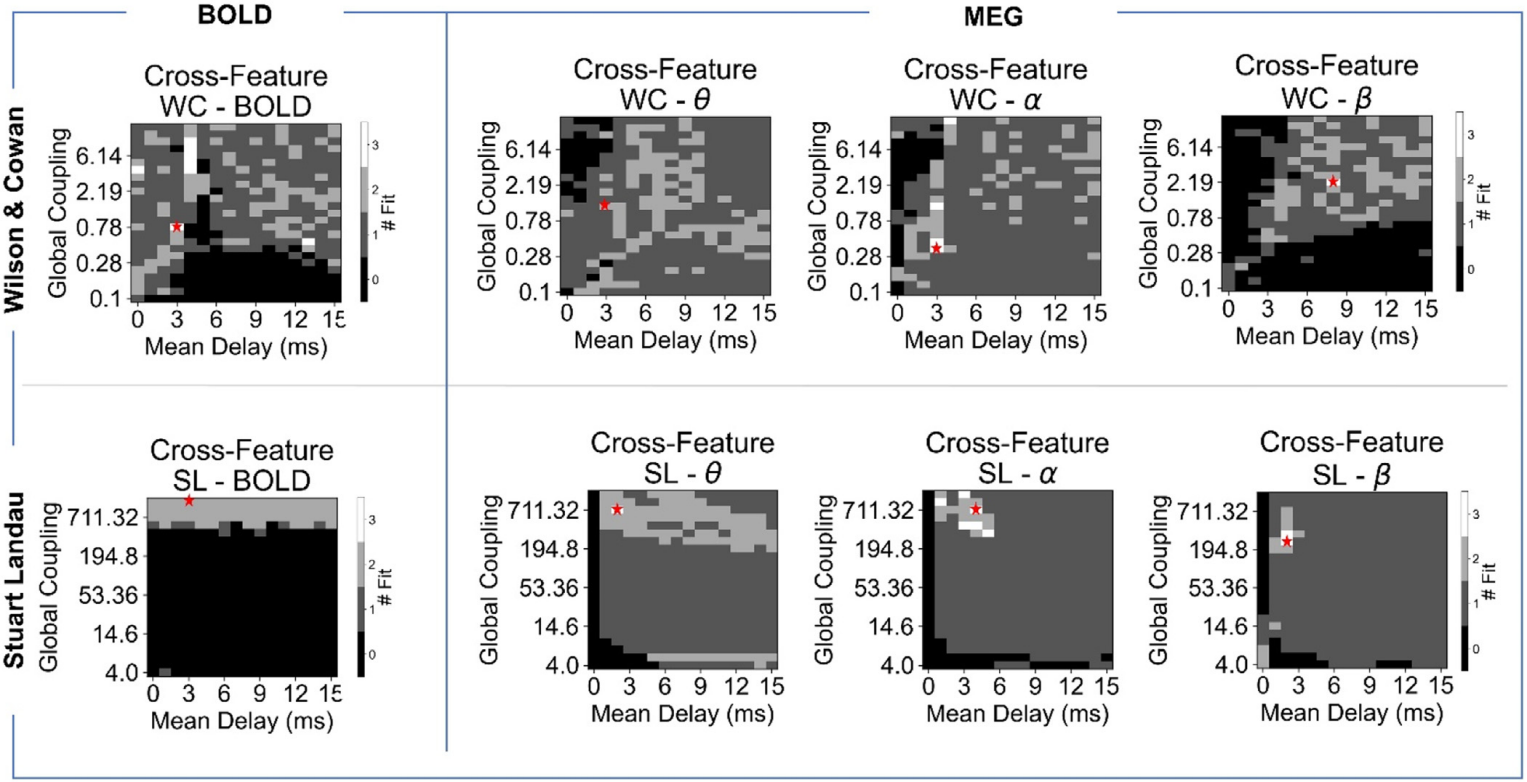
Ability of the models in simultaneously explaining connectivity and dynamic spatiotemporal features. The selected model parameter combinations for fMRI and MEG indicate the model working points (red stars), chosen through simultaneous optimisation for the representation of empirical FC, FCD and MOMs’ size, as described in the Methods section.

In this section, we investigate the ability of the two models to concurrently approximate all three features (FC, FCD and MOM sizes) for each of the four signal modalities via cross-feature fitting. The analysis evaluates the model's performance in representing the features by comparing empirical and simulated data under specific conditions: FC correlation >0.4, KS distance between FCD distributions <0.3 and KS distance between MOM size distributions <0.3 (Fig. 4).

For fMRI signals, the WC model approximates all three features within the performance criteria in a region of short delays (3–4 ms) and moderate to high coupling. This coupling range is linked to the role of E-I homoeostasis in regulating local dynamics to avoid saturation of local populations from high levels of incoming excitation. The SL model, on the other hand, fails to achieve satisfactory performance across all features: there is no region in the parameter space where all three can be represented to the required level of performance. It is true that increasing global coupling generally improves the model performance, especially for FC, but FCD and MOMs fitting remains suboptimal. The lack of empirical-like spatiotemporal dynamics of BOLD signals in the SL model indicates the importance of regulatory mechanisms for local dynamics, such as E-I homoeostasis, for slow dynamics. Additionally, for such slow fluctuations, conduction delays do not play a significant role in model performance, likely due to the diffuse nature of coupling in the SL model, which is determined by phase relations between local oscillations.

Both models struggle to achieve optimal performance in the MEG theta frequency band, possibly due to the inherent noisiness of the empirical signals. The results show that the WC model performs worse than in the other frequency bands, with no overlap between optimal regions for theta-band MEG signals. Although there is still a wide region where dynamics can be reasonably fitted in terms of both FCD and MOM size, the same is not valid for FC. In contrast, the SL model shows relevant patterns in a wide region of the parameter space in at least two of the features of interest, albeit with an overlap only in a narrow region of high coupling and short delays.

The optimal region for fitting dynamics within the MEG alpha frequency band varies by model. For the WC model, the optimal region encompasses a broad range of couplings, with a concentration around short delays (3–4 ms). This is due to the overlap between regions of optimal fit for FC (which is narrower) and dynamics (which spans a larger range of couplings and delays). Conversely, the SL model's optimal region is smaller and concentrated around short delays and high couplings, reflecting the increased role of conduction delays for phase interactions.

Lastly, in the MEG beta frequency band, no overlap exists between the region of optimal fit for FC and dynamics. The dynamics are particularly poor in the optimal FC region, leading to a single optimal point that is essentially random, located within the overall region where FCD and MOM sizes are well approximated. In contrast, the SL model adheres to the principles observed in lower frequency bands, with a small region of overlap between the optimal regions of each feature found for high coupling and fast delays.

Interestingly, the parameters corresponding to optimal fitting across modalities and models tend to fall within a region associated with moderate synchrony and high metastability (SM, Section II, Figure S5). These results follow previous works (Cabral et al., 2022; Deco et al., 2017) suggesting that metastable brain dynamics are relevant to support the spatiotemporal patterns of activity observed in empirical data. Additionally, when measuring the global peak frequency, the models show a different behaviour across parameters. However, both achieve the highest performance in a region of frequency suppression (SM, Section II, Figure S5).

Overall, our analysis unveils crucial insights into the interplay between E-I homoeostasis and delays in modelling network dynamics. Specifically, we find that E-I homoeostasis is essential for approximating spatiotemporal dynamics, especially of slow fluctuations, but also benefits fast oscillations. Moreover, delays are relevant for both models, but in different ways. In the SL model, no relevant feature of MEG signals can emerge without delays, and delays become more crucial as we move towards faster frequencies. On the other hand, in the WC model, the overlap between the optimal regions for FC and dynamics occurs in a narrow range of delays (3–4 ms). Furthermore, coupling is less important in the WC model due to the role of E-I homoeostasis in maintaining a balance between excitation and inhibition. These findings suggest that understanding the interplay between E-I homoeostasis and delays is critical for modelling large-scale network dynamics across different frequencies and modalities.

**Fig. 5:**
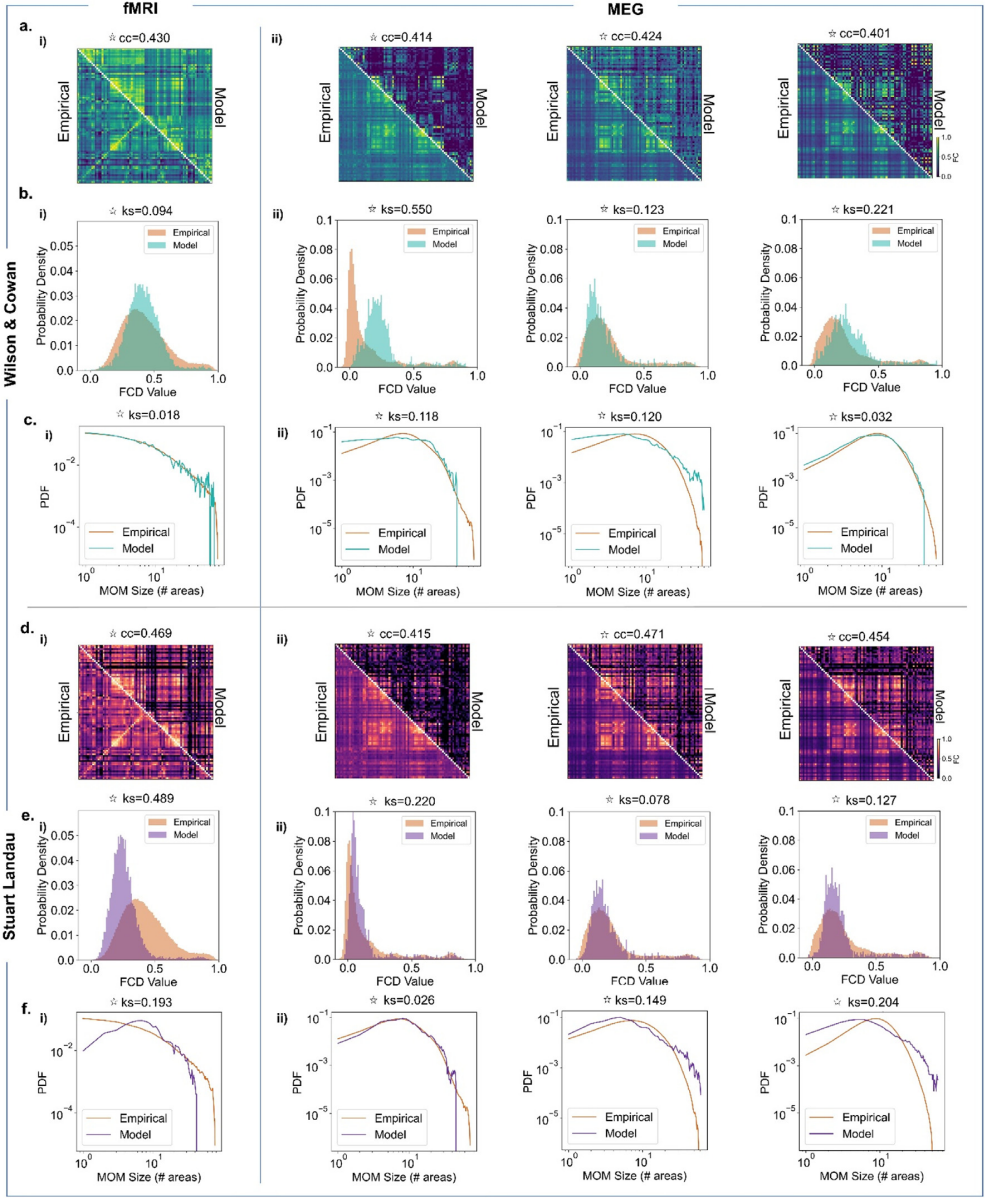
Optimal model performance in simultaneously capturing static and dynamic features: a comparative analysis of Wilson-Cowan and Stuart landau model in relation to empirical fMRI and MEG data. a. Empirical and simulated fMRI BOLD and MEG FC for 78 AAL cortical brain areas in the optimal points for the WC model. i) The selected optimal parameters for BOLD fMRI are C = 0.780, MD=3 ms, with correlation of cc= 0.430. ii) The selected WC parameters for MEG are C = 1.183, MD=3 ms for theta; C = 0.344, MD=3 ms for alpha; C = 2.19, MD=8 ms for beta with correlation values of cc_θ_ =0.414, cc_α_ =0.424, cc_β_ =0.401. b. Empirical and simulated fMRI BOLD and MEG FCD distribution in the optimal point for the WC model. i) The optimal parameters for BOLD fMRI are C = 0.780, MD=3 ms, with ks-distance value of ks=0.094. ii) The optimal parameters for MEG are C = 1.183, MD=3 ms for theta; C = 0.344, MD=3 ms for alpha; C = 2.19, MD=8 ms for beta with ks-distance values of ks_θ_ =0.550, ks_α_ =0.123, ks_β_ =0.221. c. Empirical and simulated BOLD fMRI and MEG MOM size distribution in the optimal point for the WC model. i) The optimal parameters for BOLD fMRI are C = 0.780, MD=3 ms, with ks-distance value of ks=0.018. ii) The optimal parameters for MEG are C = 1.183, MD=3 ms for theta; C = 0.344, MD=3 ms for alpha; C = 2.19, MD=8 ms for beta with ks-distance values of ks_θ_ =0.118, ks_α_ =0.120, ks_β_ =0.032. d. Empirical and simulated fMRI BOLD and MEG FC in the optimal points for the SL model. i) The selected optimal parameters for BOLD fMRI are C = 1194.16, MD=5 ms, with correlation of cc= 0.469. ii) The selected optimal WC parameters for MEG are C = 711.32 MD=2 ms for theta; C = 711.32, MD=4 ms for alpha; C = 252.4, MD=2 ms for beta with correlation values of cc_θ_ =0.415, cc_α_ =0.471, cc_β_ =0.454. e. Empirical and simulated fMRI BOLD and MEG FCD distribution in the optimal point for the SL model. i) The optimal parameters for BOLD fMRI are C = 1194.16, MD=5 ms, with ks-distance value of ks=0.489. ii) The optimal points for MEG are C = 711.32, MD=2 ms for theta; C = 711.32, MD=4 ms for alpha; C = 252.4, MD=2 ms for beta with ks distance values of ks_θ_ =0.220, ks_α_ =0.078, ks_β_ =0.127. f. Empirical and simulated BOLD fMRI and MEG MOM size distribution in the optimal point for the SL model. i) The optimal parameters for BOLD fMRI are C = 1194.16, MD=5 ms, with ks-distance value of ks=0.193. ii) The optimal points for MEG are C = 711.32, MD=2 ms for theta; C = 711.32, MD=4 ms for alpha; C = 252.4, MD=2 ms for beta with ks distance values of ks_θ_ =0.026, ks_α_ =0.149, ks_β_ =0.204.

Consequently, we identify optimal points for cross-feature representation of each of the modalities of interest (see Methods and Figure S4). Fig. 5 shows the resulting FC matrices, FCD, and MOM size distributions.

With respect to FC, all models achieve a reasonable approximation of FC patterns at the optimal points, with most modalities achieving a performance of at least 0.4. Furthermore, both models maintain a fair approximation of network dynamics at this performance level. For FCD, we observe a high proportion of correlations close to 0 for theta-band FCD in empirical data, indicating difficulty detecting transient FC patterns across time, which can explain the poorer model performance in this frequency band. Interestingly, the WC model's simulated distribution is biased towards higher values, similarly to other frequency bands.

In terms of MOM size distributions, while empirical distributions for BOLD signals lack scale-free properties observed, for example, in neural avalanches (Sorrentino et al., 2021), there is still no characteristic size (that is, a peak in the size distribution). Conversely, for MEG signals across frequency bands, empirical MOMs distributions have a characteristic size of around 7–9 areas. The WC model reveals no characteristic size in slow dynamics (i.e., BOLD fMRI), with a more pronounced characteristic size for higher frequencies. Furthermore, while we focus on the assessment of the simultaneous emergence of all the relevant features in our models, it is important to stress that, when optimised for any given feature in isolation, both models can generally attain considerably higher levels of performance (SM, Table S1).

Assessing the impact of integrating delays, quantitative results for null-delay scenarios are presented (Table 1). Interestingly, the role of delays becomes more evident when optimising individual features (SM, Section III, Table S1-S2) Table 2.

**Table 2:** Performance values, optimised for FC, FCD and MOMs size. a. Performance values in presence of delays. b. Performance values in absence of delays. Note that for Pearson correlation a greater value corresponds to a better fit whereas for KS distance it is the opposite.

a.	Best fit with delays, optimised across features (FC)				Best fit with delays, optimised across features (FCD)				Best fit with delays, optimised across features (MOM size)			
	fMRI	MEG θ	MEG α	MEG β	fMRI	MEG θ	MEG α	MEG β	fMRI	MEG θ	MEG α	MEG β
WC	0.430	0.414	0.424	0.401	0.094	0.550	0.123	0.221	0.018	0.118	0.120	0.032
SL	0.469	0.415	0.471	0.454	0.454	0.220	0.078	0.127	0.193	0.026	0.149	0.204

b.	Best fit without delays, optimised across features (FC)				Best fit without delays, optimised across features (FCD)				Best fit without delays, optimised across features (MOM size)			
	fMRI	MEG θ	MEG α	MEG β	fMRI	MEG θ	MEG α	MEG β	fMRI	MEG θ	MEG α	MEG β

WC	0.419	0.246	0.290	0.258	0.268	0.478	0.225	0.152	0.084	0.074	0.134	0.462
SL	0.458	0.165	0.267	0.460	0.584	0.366	0.735	0.126	0.205	1.000	1.000	1.000

### 3.3 Comparing models’ performance in simultaneously representing brain signals

**Fig. 6:**
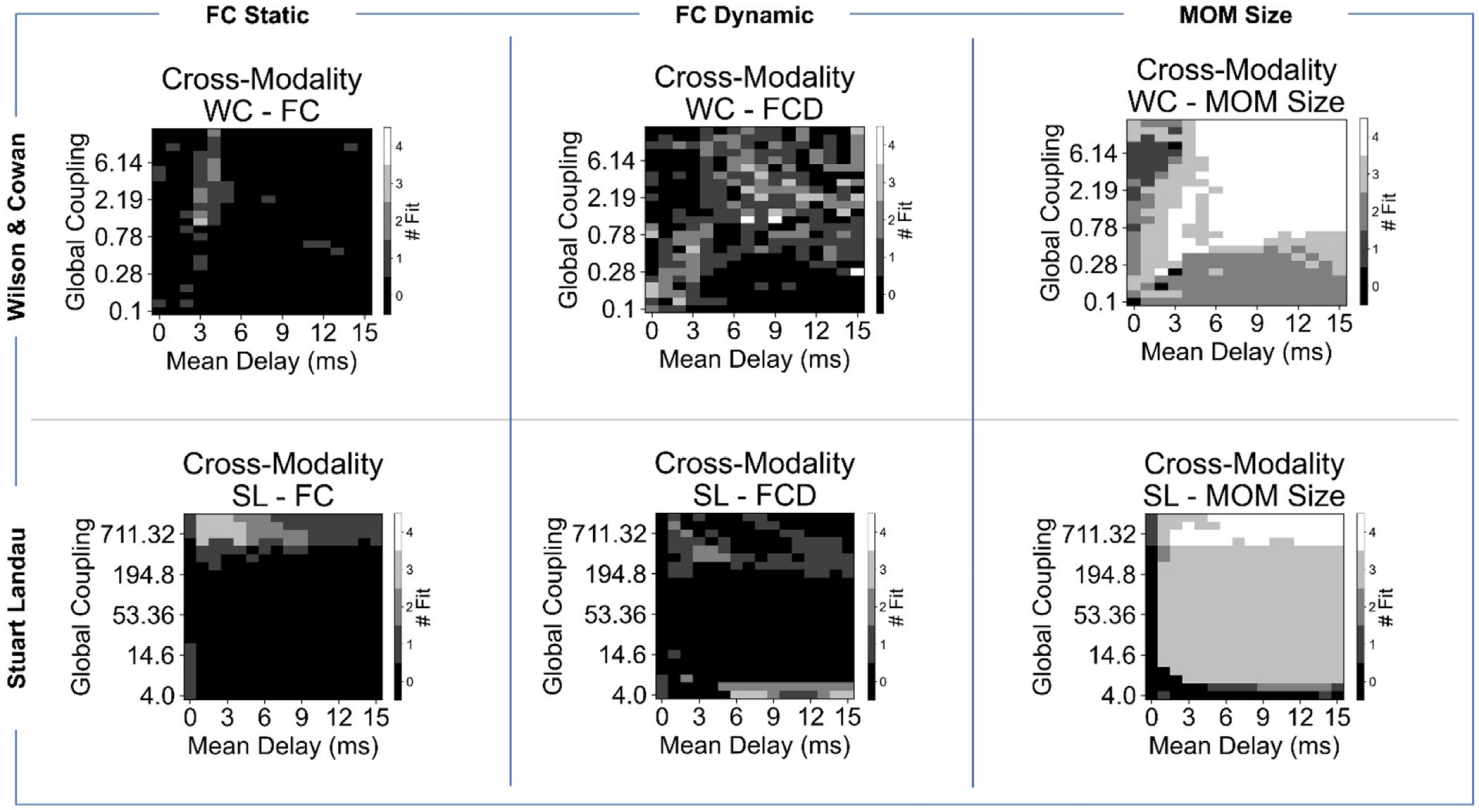
Ability of the models in explaining connectivity and dynamic spatiotemporal features across modalities. Agreement plots indicating the number of modalities (BOLD, MEG theta, MEG alpha, MEG beta) with correlation between empirical and simulated FC above 0.4 and KS distance below 0.3 (FCD and MOM size) for Wilson and Cowan and Stuart Landau model.

To summarise both models’ ability to replicate connectivity and spatiotemporal fMRI/MEG patterns, we perform a cross-modality analysis to search for a region of conjunction (Fig. 6).

In terms of FC, the WC model requires a specific range of delays to fit FC patterns across modalities, limiting overlap regions. In contrast, the SL model performs better in reproducing empirical FC across modalities, with a broader range of parameter space for adequate FC in each modality. The SL model's optimal parameters lie between 1 and 4 ms and high couplings, where cross-modality performance aligns. However, no point in either model's parameter space accurately represents FC across all modalities.

For FCD, the SL model performs considerably worse in the cross-modality analysis, with no point in the parameter space performing well for more than two modalities simultaneously. Conversely, the WC model exhibits a wide region of the parameter space where FCD fits reasonably across modalities, especially with increased global coupling and mean delays. This result suggests that the WC model's local dynamics are regulated towards a common target across the brain, through E-I homoeostasis, which renders its dynamics more robust to changes in global parameters.

Regarding MOM size, both models perform relatively well, still with differences in behaviour. The WC model approximates MOM size distributions across modalities for a broad range of parameters, and this region becomes wider as the global couplings and delays increase. In contrast, the SL model depends on global coupling for determining the size of MOMs across modalities. This result seems to contradict the findings of (Cabral et al., 2022), where delays were shown to play an important role in the emergence of MOMs. Nevertheless, it is essential to note that the threshold for detecting MOMs in Cabral and Castaldo's study was obtained from a point in the parameter space without delayed interactions, while our MOM detection criterion involves comparing instantaneous fluctuations with the level of variability of the signal itself. Therefore, given that local dynamics are essential in determining how nodes can engage in network events, our conception of MOMs is more dependant on the interplay between local and global dynamics and less directly on the presence of delayed interactions. However, the importance of delays is still evident in our results, as MOM-like dynamics can only emerge from the SL model with delays.

Overall, both models perform similarly in fitting FC across modalities, but the WC model holds a clear advantage in spatiotemporal and oscillatory dynamics, likely due to the homoeostatic effect of E-I balance, which transverses the local scale into global dynamics.

### 3.4 Functionally relevant sub-networks

In the previous results section, we discovered both models’ unsatisfactory performance in representing FC across modalities. We further investigate by measuring their performance in fitting FC sub-networks, identifying sub-networks from empirical data using a clustering method on averaged FC matrices (see Methods).

Empirical data-derived sub-networks analysis focused on six clusters of connectivity in the BOLD data, which are remarkably similar to the canonical resting-state networks (RSNs) (Lee et al., 2013; Power et al., 2011). For example, networks 1 and 2 correspond to visual and sensorimotor RSNs, respectively, while networks 4 and 6 relate to default mode and limbic networks, respectively. The sub-networks’ similarity to canonical RSNs suggests that our extraction method is able to capture the underlying functional architecture of the hemodynamic signals.

MEG networks, however, show no resemblance to canonical RSNs, appearing more spatially constrained and less distributed than some of the canonical RSNs like the default mode network (DMN) or frontoparietal network. Specifically, although the number of networks varied for all frequency bands, they appear to be distributed along an occipital-frontal axis with minimal spatial overlap.

**Fig. 7:**
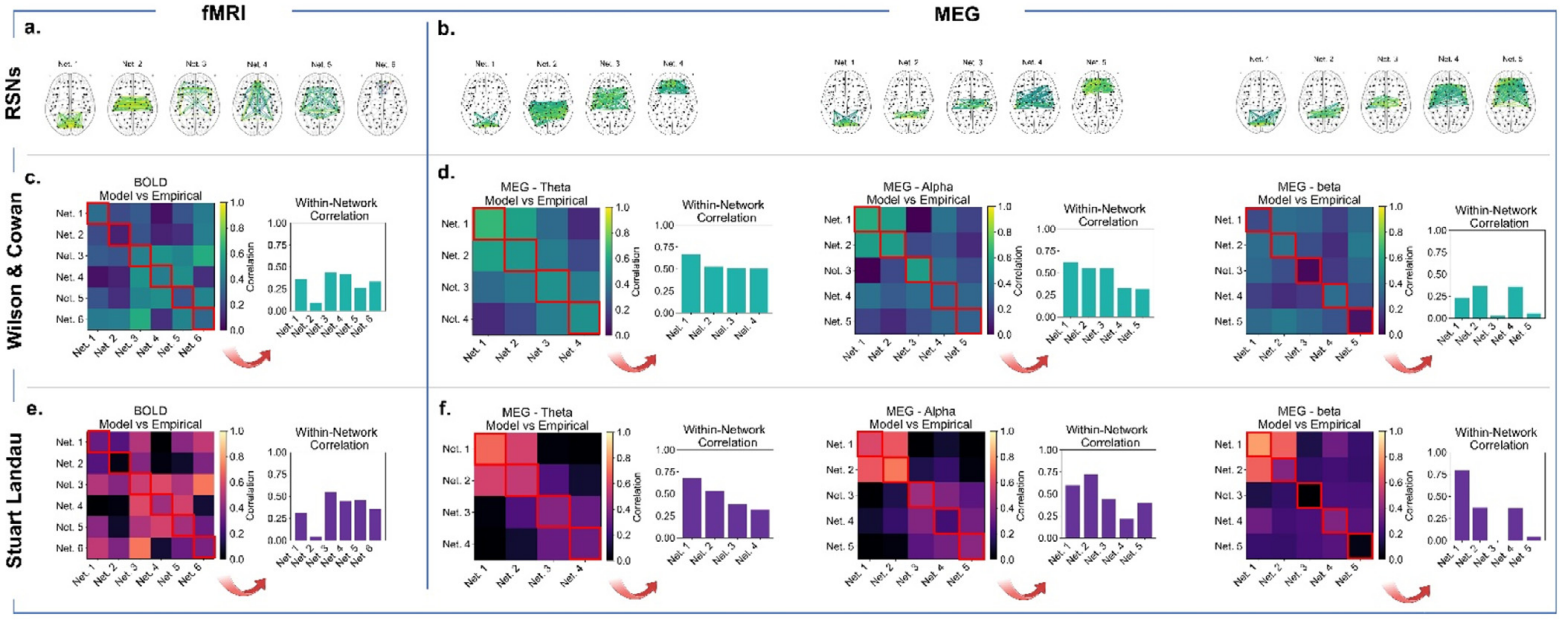
Assessing frequency-specific network correspondence between empirical and simulated functional connectivity. a. BOLD fMRI Resting state networks obtained with k-means clustering following the elbow method to choose the right number of clusters (k). b. MEG Resting state networks obtained with k-means clustering following the elbow method to choose the right number of clusters (k), for each frequency of interest. c. Network-wise functional correspondence (Pearson Correlation) for empirical and WC simulated data for BOLD fMRI. On its left side, bar plot of the values on the diagonal of the correlation matrix (highlighted in red). d. Network-wise functional correspondence for empirical and WC simulated data for MEG in theta (left), alpha (middle) and beta (right) bands (FC normalised between 0 and 1) with their correspondent bar plot of the values in the diagonal of the correlation matrix. e. Network-wise functional correspondence for empirical and SL simulated data for BOLD fMRI. On its left side, bar plot of the values on the diagonal of the correlation matrix. f. Network-wise functional correspondence for empirical and SL simulated data for MEG in theta (left), alpha (middle) and beta (right) bands with their correspondent bar plot of the values in the diagonal of the correlation matrix.

We then evaluate both model's performance in representing connectivity within and between RSNs from empirical data. To do this, we use the FC matrices displayed in Fig. 5. Our analysis indicates that both models display similar performance in representing within-network and between-network connectivity. This suggests that the issues with functional connectivity in the models are not related to the level of detail in local dynamics or inter-areal communication, but rather to the underlying anatomical framework. We discuss this in more detail in the following section. Generally, both models perform better in fitting within-network connectivity compared to between-network connectivity. This is consistent with the modularity of brain structural and functional networks, where connections within networks tend to be stronger than those between networks. We observed a similar distribution of performance across networks for both models, as shown in the matrices in Fig. 7c-f. However, connections between networks are generally weaker and more susceptible to noisy estimates, which can make them more difficult to represent accurately in models.

To further analyse the models' performance in representing FC within and between networks, we delve into the details for each modality. For BOLD, both models perform remarkably poorly in representing Network 2 (or sensorimotor), which was also poorly represented in MEG beta (Network 3). This may be related to the area's high myelination, which is not currently considered in our models (Paquola and Hong, 2023). Interestingly, both models performed better in representing more distributed BOLD fMRI networks (3 and 4) than more localized ones (1, 2, and 6). In the context of MEG, the WC model has better performance in representing between-network connectivity, particularly in theta and alpha frequencies, while within-network frequency remains similar for both models. Another interesting feature, observed in both models but more strongly in the SL, was a decrease in performance from more posterior to more anterior regions across frequencies.

In summary, we observe varying performance depending on the network and modality. However, no salient differences in FC pattern approximation exist between models, even at this level of detail, suggesting that the limitations in accurately representing FC are likely related to the underlying anatomical framework, rather than the level of detail in local dynamics.

### 3.5 The role of the connectome

**Fig. 8:**
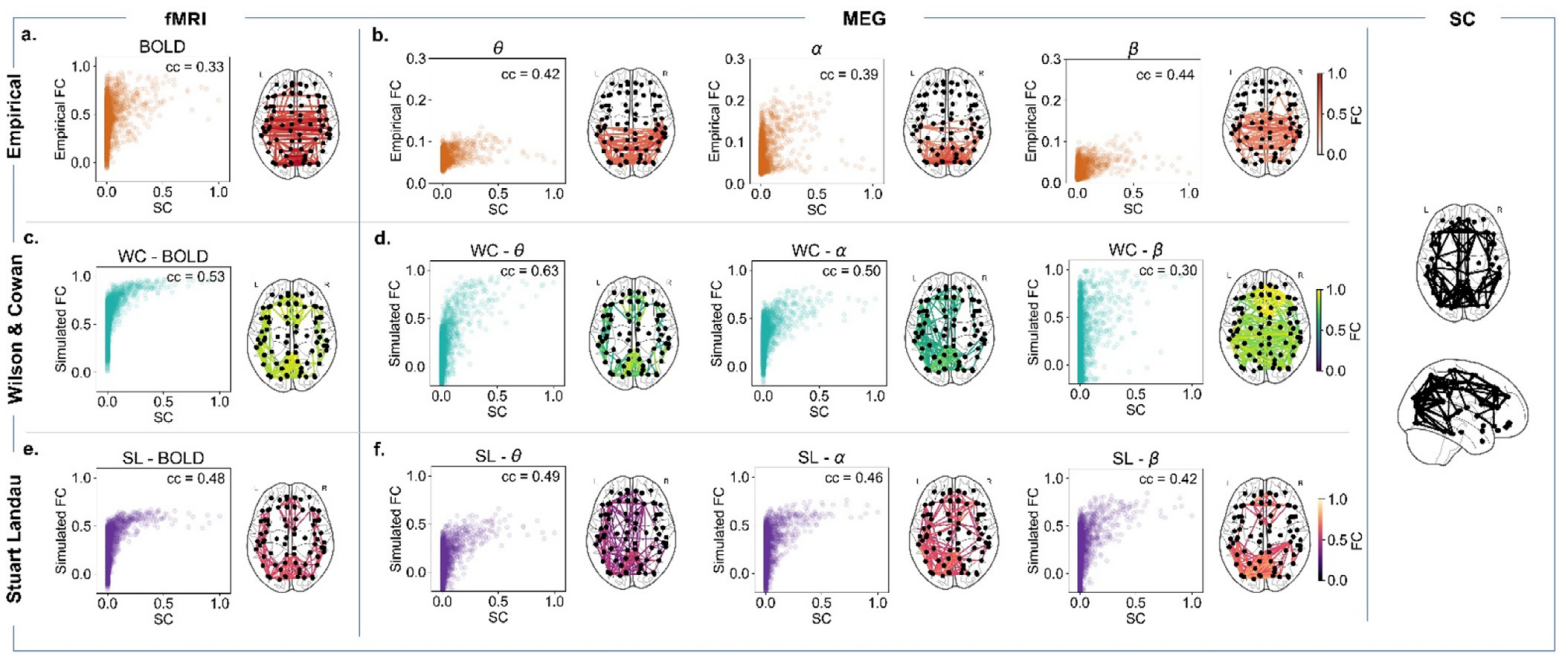
The role of the connectome. a. Scatter plot of empirical BOLD fMRI Functional Connectivity (FC) versus Structural connectivity (SC) with a correlation value of cc=0.33. b. *Left* - Scatter plot of MEG theta FC versus SC with a correlation value of cc=0.42. *Middle -* Scatter plot of MEG alpha FC versus SC with a correlation value of cc=0.39. *Right -* Scatter plot of MEG beta FC versus SC with a correlation value of cc=0.44. Brain plots showing the 5% strongest connections of empirical FC. c. Scatter plot of simulated WC BOLD fMRI FC versus SC, with a correlation value of cc=0.53. d. *Left -* Scatter plot of simulated WC MEG theta FC versus SC, with a correlation value of cc=0.63. *Middle -* Scatter plot of simulated WC MEG alpha FC versus SC, with a correlation value of cc=0.50. *Right -* Scatter plot of simulated WC MEG beta FC versus SC, with a correlation value of cc=0.30. e. Scatter plot of simulated SL BOLD fMRI FC versus SC, with a correlation value of cc=0.48. f. *Left -* Scatter plot of simulated SL MEG theta FC versus SC, with a correlation value of cc=0.49. *Middle -* Scatter plot of simulated SL MEG alpha FC versus SC, with a correlation value of cc=0.46. *Right -* Scatter plot of simulated SL MEG beta FC versus SC, with a correlation value of cc=0.42. Brain plots showing the 5% strongest connections of simulated FC.

The repertoire of functional networks lies upon the hidden structural architecture of connections that facilitates hierarchical functional integration (Park and Friston 2013). Here, we explore the performance of two large-scale generative models, with the goal of understanding the underlying processes giving rise to coherent large-scale functional networks. Nonetheless, both models have the human DTI-based structural connectome as the only empirically derived element. Such models can also be understood as a nonlinear system which, taking the connectome as the input, can be used to evaluate the possible causal mechanisms for a phenomenon of interest to emerge (i.e., FC patterns). Given the limitations of both models in the representation of empirical FC, particularly at the level of its subnetworks, we investigate the non-trivial structure-function relationship. The magnified correlation between simulated FC and SC (Fig. 8) may explain the models’ predictive ability not reaching higher performance levels.

More specifically, as shown in Fig. 8c/f, the functional topology patterns appear largely constrained by structure, regardless of the model implemented — the relationship between FC and SC remains the same for both models. Furthermore, relating simulated FC with more detailed graph properties of the underlying might help elucidate generative processes in our models that might hinder the representation of relevant FC patterns (SM, Section IV, Figure S16). For the SL model, structural communicability (reflective the efficiency with which information can be communicated between two give nodes) (Estrada et al., 2012), appears to be the most consistent defining factor of simulated FC, highlighting the role of diffuse interactions in shaping model activity (Fornito et al., 2016). Conversely, for the WC model, the picture is more complex. In general, we observe a stronger correlation between FC and SC, associated to a high correlation with Euclidean distance between nodes, either stronger or quantitatively similar to the values in empirical data. The exception is beta band connectivity, where the highest correlation is found with communicability. However, we stress the fact that, due to our cross-feature optimization, the particular working point chosen for the models is optimised not only for FC, but also dynamics. This is evidenced especially in the beta-band, where the optimal regions do not overlap significantly, and the chosen point is more optimised for dynamics. Therefore, results in the beta-band should be interpreted with care. Importantly, these findings are stronger when optimising both models for FC only (SM, Section IV, Figure S17). To conclude, the SL model is mostly constrained by the communicability of the underlying SC, while FC in the WC model is more reflective of the weight of structural connections, including the known exponential decay rule (EDR) of connectivity with distance (Ercsey-Ravasz et al., 2013).

On another note, similar conclusions can be drawn when observing the topography of the most descriptive spatial patterns of MOMs (SM, Section III, Figure S13). While empirical MOMs reflect the canonical RSNs, in BOLD signals, and the same spatially constrained networks observed for static MEG connectivity across frequency bands, the same patterns are not observed in simulated data. Conversely, the MOM patterns generated by both models are not as reflective of the resting-state networks. More specifically, we mainly observe MOMs that are spatially constrained across all modalities, where activity is confined within a well-defined region of the human cortex (such as the occipital or temporal lobes). In addition, the measured patterns for BOLD and all three MEG bands are considerably more similar between each other than the ones observed in empirical data, suggesting a strong role of structural connectivity in defining simulated MOMs across modalities.

Nonetheless, despite the constraints imposed by SC, realistic between-area connections are still a relevant piece in biophysical models, since without the right structure (e.g., shuffling SC connections while maintaining the distribution of weights and symmetry), there can be no emergence of function (SM, Section IV, Figure S15).

## 4 Discussion

MEG and BOLD signals are believed to reflect two different aspects of neural activity, occurring at timescales that are orders of magnitude apart. While BOLD signals are thought to represent changes in haemodynamics (Hillman, 2014) — likely triggered by synaptic transmission, the most energy intensive process in the human brain (Harris et al., 2012) —, MEG signals reflect changes in magnetic fields created by dipole currents that flow along neuronal processes (Lopes da Silva, 2013). These dipole currents depend on dendritic synaptic input (Lopes da Silva, 2013) and are, therefore, related to the same processes involved in the generation of BOLD signals. Nonetheless, even though both modalities share the same neural substrate, large-scale models, to date, are usually tailored to represent only one modality at a time. A common example is the practice of tuning the intrinsic frequency of oscillation of local populations to the frequencies of interest in the respective modalities (e.g. ∼10 Hz for MEG, <0.01 Hz for BOLD) (Abeysuriya et al., 2018; Deco et al., 2017, 2017). In this work, we argue that models should be able to simultaneously generate multiresolution modalities with the same underlying generative (neuronal) mechanisms, without tuning parameters *a priori* to selectively reproduce features of interest. Combining multiresolution multimodal data with large-scale modelling, potentially allows one to disentangle the generative mechanisms behind brain function and its dynamical underpinnings from its multiscale expression in various measurement modalities.

Our first step towards cross-modality convergence involves imposing an intrinsic oscillation frequency of ∼40 Hz, in the gamma range. Gamma rhythms in the human cortex are believed to be generated by a myriad of mechanisms, including reciprocal interactions between pyramidal neurons and fast-spiking interneurons (Buzsáki, 2006) (Buzsáki and Wang, 2012). Indeed, BOLD signal fluctuations have been hypothesised to emerge from changes in synchrony between gamma-band oscillations (Deco et al., 2009). Moreover, recent findings suggest that functional networks in lower-frequency bands (i.e. theta, alpha and beta) can be generated through delayed interactions between gamma oscillators (Cabral et al., 2022). Therefore, multiresolution recordings might reveal distinct facets of gamma activity, and models with local gamma oscillations might reproduce empirical properties of both BOLD and MEG FC. Our approach extends beyond assessing model performance across modalities. Although the field of large-scale modelling has primarily focused on reproducing functional connectivity (Cabral et al., 2011; Coombes, 2005; Deco et al., 2008; Deco et al., 2013; Honey et al., 2007, 2010), research indicates that functional networks are, in fact, dynamic (Schirner et al., 2022; Vidaurre et al., 2017) and that FC dynamics are linked to healthy brain function features, such as metastability (Deco et al., 2017), and might support crucial cognitive processes (Bonkhoff et al., 2021; Filippi et al., 2019). In addition, recent results show network-wide engagement in transient oscillatory modes as an important emergent feature of brain networks (Cabral et al., 2022). Consequently, we argue that brain network models should reflect not only statistical dependencies amongst brain signals (i.e., FC), but also the dynamics underlying the spontaneous and transient appearance of functional networks. Hence, we examine model performance in representing not only FC, but also dynamics (FCD) and transient oscillatory modes (MOMs), exploring the role of properties such as axonal conduction delays and local E-I balance in representing static and dynamic network features.

### 4.1 Role of delayed interactions

Conduction delays have been shown to provide a rich dynamic framework for the emergence of resting brain oscillations (Abeysuriya et al., 2018; Cabral et al., 2014; Petkoski and Jirsa, 2019). Building on prior research (Cabral et al., 2022), incorporating delays significantly improves model performance in explaining empirical MEG static and dynamic patterns.

A notable aspect of the SL model in our results is the diminishing role of delays for the emergence of network features of slower fluctuations. We posit that the decreased dependence of low-frequency oscillatory patterns on the mean delay (for the explored range) is related to the ratio between oscillations’ period and the delay itself. For instance, while a 10 ms change in the mean delay represents 25% of a beta rhythm cycle (∼25 Hz, 40 ms period), the same change accounts for only 0.01% of a slower BOLD rhythm cycle (∼0.01 Hz, 100 s period). Thus, we hypothesise that, for higher frequency bands, changes in conduction velocity have a more substantial impact on the ability of regions to synchronise at those frequencies due to larger phase-relationship alterations.

On this note, for the WC model, the relevance of delays is evident across all the analysed modalities, emphasising their importance for generating even slow signals, such as BOLD fluctuations, when implementing neural mass models with “synaptic-like” communication between nodes (i.e., WC models). Accordingly, previous research using similar models informed by a macaque connectome (Deco et al., 2009) revealed that BOLD signal fluctuations could be generated by transient synchronisation of coupled Wilson-Cowan nodes resonating at 40 Hz. Importantly, the same model was sensitive to changes in conduction velocity, showing an optimal range of conduction speeds, even without modelling local E-I balance.

Overall, these results emphasise the importance of delayed interactions, especially when seeking a unified explanation for static and dynamic features across various neuroimaging modalities. Within our multi-modal framework, founded on underlying interactions between gamma oscillators, it becomes evident that inter-areal conduction plays a complex and crucial role in the emergence of relevant spatiotemporal dynamics.

Our conclusions align with previous modelling results suggesting that deficits in the regulation of axonal myelination could significantly impact the ability of coupled oscillators to synchronise at high frequencies (Pajevic et al., 2014). Furthermore, the profound importance of modelling conduction delays was established using Bayesian model comparison (comparing models with and without delays) at the inception of dynamic causal modelling for fast, event-related responses as measured with EEG (David et al., 2006).

The importance of modelling delays raises questions about their role in the brain. Our results suggest that conduction delays underscore the emergence of relevant dynamics, especially when in frequency-specific oscillatory bands (high-frequency in SL, across frequencies for WC). Therefore, it is reasonable to expect axonal conduction velocities to be precisely structured in the human brain. However, empirical data reveal a high level of heterogeneity in the distribution of axonal diameters and levels of myelination, both of which determine conduction speeds (Saab and Nave, 2017; Sorrentino et al., 2022), with complex interactions between the two (Waxman, 1980). Moreover, research suggests a dynamical regulation of myelination, at least in sensory systems (Saab and Nave, 2017). We suggest that such heterogeneities, including activity-dependant myelination (Noori et al., 2020; Pathak et al., 2022), are crucial aspects of computational architectures and message passing in the brain. Accounting for heterogeneous conduction velocities could help large-scale models — such as the ones implemented here — to better explain empirical patterns of MEG connectivity, which are less clearly constrained by structural connectivity.

### 4.2 Role of E-I balance

The well-documented significance of excitatory-inhibitory (E/I) balance for cortical function (Dehghani et al., 2016; Froemke et al., 2007; Páscoa dos Santos et al., 2022; Sprekeler, 2017; Tao and Poo, 2005; Xue et al., 2014), and the presence of synaptic plasticity in response to perturbations and developmental changes (Ma et al., 2019; Turrigiano, 2011; Turrigiano et al., 1998; Vogels et al., 2011) underpins the extended WC approach, building upon established work on neural-mass models with excitatory and inhibitory populations (Abeysuriya et al., 2018; Deco et al., 2019; Hellyer et al., 2016). Furthermore, since our cortical connectome has a wide range of node degrees (sum of incoming connections to a node) — which vary by at least one order of magnitude — nodes receive varying levels of excitatory input. Therefore, through the process of E-I homoeostasis local dynamics are adapted to such discrepancies, allowing cortical areas to maintain their responsiveness to network level events and supporting balanced propagation of activity (Hellyer et al., 2016; Ma et al., 2019). Indeed, our results suggest that neglecting local E-I balance in the WC model strongly affects the models’ ability to exhibit empirical-like network dynamics (SM, Section III, Figure S6). The pronounced impact of MOMs underscores the pivotal role of local E-I balance in supporting network-wide events, likely by optimising the responsiveness of cortical networks at the mesoscale.

Furthermore, the WC model with plasticity reproduces FCD features more robustly than the SL, particularly regarding BOLD signals, which the SL model fails to replicate. In fact, relevant patterns of BOLD FCD could not be observed in the SL model, for any combination of parameters within the explored range. Although the WC model fails to approximate MEG theta FCD when optimising for all measured features (Fig. 3), it performs equitably or better than the SL model when optimising for FCD or MOM sizes individually (SM, Section III, Table S1-S2). More importantly, when optimising models across modalities, the WC model consistently outperforms the SL by enabling a larger region in the parameter space where relevant network dynamics manifest across frequency bands and signal modalities (Fig. 6). This confirms the pivotal role of local E-I homoeostasis for global dynamics - especially in slow fluctuations - extending its influence beyond the mesoscale and toward the emergence of global spatiotemporal dynamics. Given the demonstrable relevance of FCD (Deco et al., 2017) and metastable oscillatory dynamics (Cabral et al., 2022) for distributed neural processes associated with higher-order cognition (Deco et al., 2021, 2017; Filippi et al., 2019), we suggest mesoscale E-I balance as one of the fundamental mechanisms that scaffold large-scale brain dynamics.

### 4.3 Structure-function relationship

In modelling large-scale brain activity on a structural connectome substrate, it is vital for models to support the emergence of functional structures beyond those dictated solely by the structural connectome. By contrasting the correlation between FC and SC in both empirical and simulated data, our results show that, while both models can reasonably approximate empirical FC (Table 2 and SM, Section III, Table S1), simulated FC is considerably more correlated with SC than what is observed for empirical data. More specifically, the most defining patterns of functional connectivity, in both models and across modalities, strongly reflect the underlying anatomical framework, instead of the particular spatial organisation that we find in empirical data (e.g., strong beta band connectivity around the pre-central gyrus) (Fig. 8). More relevantly, our analysis reveals the nature of structural constraints imposed by SC in each of the models. FC patterns in the WC model are more reflective of the weights of the underlying SC, and thus also display a clearer relationship with Euclidean distance between nodes, which is characteristic of the structural connectome (Ercsey-Ravasz et al., 2013). Conversely, FC in the SL model is strongly correlated with communicability, which is a metric of the efficiency in communication between two given nodes (Estrada et al., 2012). Since communicability reflects diffusive interactions between nodes (Fornito et al., 2016), we argue that this effect is likely a consequence of the implementation of diffusive coupling in the SL model (see Methods). Nonetheless, even though both models show different structure-function relationships, we argue that the constraints imposed by structural connectivity on each model affect the approximation of empirical FC to a comparable degree.

Two potential, non-exclusive interpretations arise from this result. First, both modelling approaches are overly constrained by the structural connectome, which lacks information about the strength of effective connectivity and the direction of connectivity (e.g., forwards versus backwards). In addition, research suggests that there are gradients in microcircuitry organisation, such as asymmetries in laminar-specific forward and backward connections and recurrent excitation or the distribution of inhibitory interneurons (Wang, 2020), that reflect the hierarchical organisation of the human cortex (Felleman and Van Essen, 1991). Not only is this hierarchical organisation functionally relevant for processes such as perception (van Vugt et al., 2018; Wyss et al., 2006) and memory (Froudist-Walsh et al., 2021), but recent modelling results show that accounting for these asymmetries improves the reproduction of FC and FCD, while allowing for the emergence of important (i.e., non-dissipative) features of brain activity, such as ignition dynamics (Deco et al., 2021). Furthermore, the spatial distribution of such asymmetries and variations in synaptic time constants might explain why particular frequency bands are more prominent in certain anatomical regions, as is the case of beta in the parietal cortex and alpha in the occipital lobe. Indeed, myelination imaging indicates that these regions include areas with the highest myelin content (Glasser et al., 2016; Rowley et al., 2015), which could relate to higher conduction speeds (Saab and Nave, 2017), favourable to the emergence of relevant functional networks at these higher frequencies. Second, structural connections may be underestimated using tractography. One example is the limited ability of DTI to estimate interhemispheric white matter tracts, leading to a difficulty in reproducing the strong homotopic interhemispheric functional correlations present in fMRI (Deco et al., 2013). Additionally, recent results show that communication between cortical areas at different frequency bands has varying degrees of dependence on the underlying anatomy (Vezoli et al., 2021), suggesting that empirical FC reflects processes that go beyond structure.

Furthermore, our exploration of model performance in the representation of FC at the sub-network level (Fig. 7) reveals varying levels of performance for specific networks. In fMRI, the representation of networks associated with visual (Net. 1) and, especially, motor areas (Net. 2) is particularly low. For MEG signals, we found a general decrease in performance along the occipital-frontal axis across all three frequency bands. We suggest that both issues can be reflective of the lack of empirically derived sources of heterogeneity in cortical circuitry. First, visual and motor areas are associated with increased myelination (Glasser et al., 2016; Rowley et al., 2015), the lack of which could affect model performance in representing these networks. Second, the fact that adjustments in microcircuitry across the cortical hierarchy (Wang, 2020), such as increased recurrent excitation in more frontal areas, were not accounted for might explain the decreased performance for more frontal networks of MEG FC. Importantly, both models reveal a similar behaviour in the representation of FC sub-networks (Fig. 7), suggesting that the discussed issues arise from the underlying structural framework, which is common to the two models.

Nonetheless, both models can account for emergent properties of human FC not solely captured by structural connectivity. Besides stressing the role of non-linear dynamics and interactions in brain networks, this further establishes the validity of integrating delayed interactions in models for the prediction of even static FC (Cabral et al., 2011; Deco et al., 2009). In fact, although there is an established exponential relationship between connection strength and distance (Ercsey-Ravasz et al., 2013), there are exceptions to this rule, shown to be relevant for large-scale functional networks (Deco et al., 2021). Therefore, we argue that the conduction delays between areas enrich models beyond the underlying structural framework. Furthermore, although the WC model with plasticity performed better when reproducing dynamical spatiotemporal features (FCD and MOMs), especially for BOLD, its added complexity did not guarantee a better approximation of functional patterns. This suggests that models could benefit from the inclusion of more detailed empirically-derived information about sources of heterogeneity such as local microcircuitry (Wang, 2020) or myelination (Boshkovski et al., 2021) as discussed above.

### 4.4 Metastable Oscillatory Modes

Analysis of recurrent metastable oscillatory modes (MOMs) may elucidate the mechanisms behind the functional integration - segregation relationship (Friston, 1997, 2000). As shown in recent work (Cabral et al., 2022), and further validated in the current study, when the coupling is sufficiently strong, the emergent dynamics will start to resemble the complex and intermittent dynamics observed in neuronal timeseries. As we further increase the extrinsic coupling of our models, the system locks into a regime of complete entrainment losing the frequency-specific intermittency.

Our results suggest that self-limiting transient oscillations are also detectable in signals with spontaneous sustained periodicity, such as fMRI timeseries. This is in line with the notion that synchronisation underscores fMRI correlations (Lu et al., 2007) and the potential of fMRI to map neural oscillations (Lewis et al., 2016), suggesting the possible coexistence of both transient events and sustained oscillations in the brain (van Ede et al., 2018).

In this work, we focus on the distributions of MOM sizes (that is, the number of regions engaged in a MOM at a given point in time) as a point of comparison between empirical and simulated data. Importantly, while still reflective of network dynamics, this approach differs fundamentally from FCD since MOM sizes are not directly informative of the spatial topology of transient oscillatory modes, but instead of the statistics of their propagation through the network. Nonetheless, since models constrained by a shuffled connectome (while maintaining the same distribution of weights) are not able to approximate empirical MOM size distributions, particularly the characteristic sizes of MEG MOMs (SM, Section IV, Figure S15), we argue that the statistics observed in empirical data are still informed by the architecture of functional interactions.

Conversely, we perform a similar analysis for MOM durations (i.e., how long the network is engaged in a MOM). In general, model performance is satisfactory across the parameter space, given that delayed interactions are considered for all modalities. This might suggest that the distribution of MOM durations, and particularly their characteristic values (corresponding to peaks in the probability distribution), are inherent properties of how MOMs are extracted from the data. Indeed, higher-frequency MOMs show shorter characteristic durations and, when plotted as the number of cycles, the characteristic duration of MOMs falls between 0.5 and 2 cycles for all modalities (SM, Section III, Figure S11). Therefore, MOM size distributions are more closely related to the network architecture and better evaluate model performance in fitting empirical spatiotemporal dynamics. In addition, the topography of empirical MOMs is also reminiscent of general principles of the functional organisation of the brain, such as the canonical BOLD fMRI RSNs (SM, Section III, Figure S13).

On another note, the relevance of local E-I balance in the emergence of empirical-like MOM dynamics is evidenced through the deterioration of performance in the WC model when plasticity is removed (SM, Section III, Figure S6) and the balanced WC model outperforming the SL in representing MOMs across modalities (Fig. 3). That said, while it is known that cortical neurons maintain E-I balance through a variety of homoeostatic mechanisms (Turrigiano, 2011), crucial for regulating mesoscale dynamics (Ma et al., 2019), our results suggest that these balancing mechanisms might have broader implications on a larger scale, affecting micro to macroscale dynamics. Nonetheless, our results do not detract from the relevance of conduction delays, since they are still required in the SL model for the generation of MOMs and become increasingly important in the WC model as we move towards higher frequency bands (both for sizes and durations) (Cabral et al., 2022). Therefore, we argue that, while it is true that local E-I balance facilitates the occurrence and propagation of MOMs, appropriate inter-areal conduction delays are nonetheless an essential factor in shaping the patterns of oscillatory interaction observed in large-scale cortical dynamics.

On a different note, both models include noise. This may lead to questioning if the observed transient stability of oscillatory modes is genuinely due to intrinsic metastability or rather due to noise-driven transitions between multiple stable attractors. However, we underline that the properties of the MOMs vary across the parameter space (Fig. 3), suggesting that the emergence of empirical-like oscillatory transients is not solely reliant on noise-driven oscillations but also on the interaction of global parameters and local E-I balance (for the WC model). Furthermore, previous studies have found that at the border between synchrony and asynchrony, coupled oscillator systems with heterogeneous delays exhibit non-steady order parameters even in the absence of noise, and the system switches constantly between different coherent pseudo-attractors, never setting in a given attractor (Lee et al., 2009; Niebur et al., 1991).

Given the similar properties of the oscillatory modes observed in simulations in both models and in real data, we keep the term Metastable Oscillatory Modes, assuming the universality of this phenomenon to more complex models even in the presence of low levels of noise.

## 5 Limitations and future work

### 5.1 Averaging (over subjects)

We used the structural connectome - derived from the average of 32 DTI scans - in this work to define the connectivity matrix. These data were acquired as part of a study separate from the MEG and fMRI HCP data. Averaging over subjects in DTI studies is deemed a necessary step in order to reduce the effect of signal loss due to changes in local magnetic susceptibility, which can lead to the aberrant inferences about diffusion direction being estimated and false positives and false negatives (Damoiseaux and Greicius, 2009).

In effect, we used the average structural connectivity matrix derived from one group to reproduce functional data similar to another group. We suspect that this may have limited our ability to find better correlations between the real and synthetic FCs. This issue suggests a similar analysis, in the future, where an individual's tractography image is used to predict that subject's MEG and fMRI features. In order to leverage the improved SNR of group-average data while accommodating heterogeneity over subjects (Quinn et al., 2021; Wens et al., 2014), a hierarchical model could be entertained.

### 5.2 MEG source reconstruction

Beamformers are a popular method for source reconstruction within the field of MEG, and have been used in FC studies (e.g. (Baker et al., 2014; Brookes et al., 2011; Hipp et al., 2012; Liuzzi et al., 2017). Often, they are chosen because of their ability to suppress sources of interference from outside source space (Boto et al., 2021; Cheyne et al., 2007; Litvak et al., 2010).

Despite their simplicity and popularity, beamformers are limited in the sense that they are, fundamentally, a spatial filter and therefore lack a generative model. This can make comparisons between alternative source inversion results non-trivial. Moreover, beamformers are known to suppress brain areas which exhibit high areas of zero-phase-lag (instantaneous) connections, i.e. correlated sources (Van Veen et al., 1997). Recent work has shown that using a beamformer to study the default mode network (DMN) at rest can be pernicious (Sjøgård et al., 2019). This provides an argument for using a source inversion algorithm with a full generative (i.e., forward) model which can account for correlations between brain areas in the source space, e.g. CHAMPAGNE (Owen et al., 2012) or Multiple Sparse Priors (MSPs) (Friston et al., 2008). However, at the time of writing, MSPs has been primarily optimised for time-averaged data and cannot readily be applied to resting-state scans.

An alternative approach - that we could have adopted in this work - would have been to side-step the ill-posed inverse problem altogether and instead focus efforts on maximising the similarity between sensor level covariance matrices (or some other statistic) of the simulated and real MEG datasets. This would have removed the confound of source leakage during the model screening process, although we would have to have accounted for variations in head position and greater levels of sensor noise which the beamformer implicitly reduces.

### 5.3 MEG FC and FCD

In MEG, FC quantifies how the brain organises itself into macroscopic functional networks across and within frequency bands (Sadaghiani et al., 2022). In this work, we use the amplitude-envelope-correlation (AEC) to quantify FC, a metric which assesses the correlation between the power envelope of two neural signals from different brain regions. Here, MEG AEC is measured as the correlation of the slow temporal fluctuations (envelope) of the orthogonalized MEG signals (for a comprehensive review, refer to (O'Neill et al., 2018). This method has an established reliability and reproducibility in FC research (Colclough et al., 2015). It has been used in biophysical generative modelling (Abeysuriya et al., 2018; Cabral et al., 2022, 2014; Schirner et al., 2022), and has enjoyed widespread use within the M/EEG FC literature (Brookes et al., 2011). This metric of FC enables us to compare outcomes amongst diverse large-scale modelling approaches, making it a suitable benchmark for comparing the number of connections detected across models and modalities. Moreover, we sought a method that has proven reliable for extracting a variety of features measured in our work, such as FC (Colclough et al., 2015), FCD (Liuzzi et al., 2019) and MOMs (Cabral et al., 2022). Additionally, studies examining MEG FC and its relationship to fMRI (de Pasquale et al., 2010; Liu et al., 2010) have both employed amplitude envelope-based measurements. This evidence further bolstered our choice of AEC methodology. Whilst reliable, the AEC method is blind to non-linear interactions between source envelope timeseries, as well as phase information. The question of whether incorporating this type of information enhances the robustness of connectivity measures and model performance remains a subject of debate. Some studies have demonstrated the benefits of utilising measures such as Multi-scale Rank-Vector Entropy (Godfrey and Singh, 2021) and phase-based measures (Abeysuriya et al., 2018), suggesting that these methods can provide valuable additional information. However, a recent analytical study argues that the AEC measure contains highly physiologically relevant information about the co-occurrence of bursts that might be missed when using phase-based measures alone (Hindriks and Tewarie, 2023). This implies that the AEC method has unique merits in capturing certain aspects of brain connectivity that cannot be fully represented by phase-based measures.

In the context of FCD, while the methodology for its computation is more established in fMRI research (Deco et al., 2017, 2021, 2017; Kong et al., 2021), the same cannot be said for MEG signals, which not only display faster fluctuations, but also a wider range of timescales over which they occur (Liuzzi et al., 2019). With that in mind, two different approaches can be taken when measuring MEG FCD. The first is to focus on the slow fluctuations in amplitude envelopes, as in recent studies (Portoles et al., 2022), which means one might disregard faster dynamics. This approach has been previously applied to compare empirical and simulated data (Portoles et al., 2022), and offers a way of harmonising the timescale used to characterise data across frequency bands and between BOLD and MEG signals. The second approach is to use adaptive windows, not only dependant on the modality of interest, but also dynamically changing in time, as suggested by (Liuzzi et al., 2019). This allows one to capture FC dynamics at a wider range of timescales with fewer a-priori assumptions, but results become more difficult to compare across frequency bands and modalities. In our work, we opted for the first choice, given recent papers where the same technique was used for model validation (Portoles et al., 2022), and to ensure that the timescales that we characterised were closer to the more established fMRI FCD. Our results show that the distributions of alpha and beta FCD have a similar shape to BOLD (Fig. 5), suggesting that they reflect the same underlying network dynamics, albeit supported by substantially different rhythms. Conversely, empirical theta FCD distributions were closer to what would be expected from a noisy signal (that is, centred around zero, with small peaks over higher correlations due to the use of overlapping windows). This is particularly evident in the WC model, where — similarly to the other frequency bands — FCD correlations are biased towards correlations higher than zero.

Numerous methods have been employed when investigating functionally relevant sub-networks in resting state activity. Notably, seed-based methods (Fox et al., 2006; Greicius et al., 2003; Vincent et al., 2008), independent components analysis (ICA) (Brookes et al., 2011; Lee et al., 2012), graph methods (Power et al., 2011), k-means clustering algorithm (Golland et al., 2008; Jia et al., 2022), and fuzzy-c-means clustering (Lee et al., 2012) have been widely utilised. However, in the context of MEG sub-networks, the use of the k-means clustering algorithm for detecting resting-state networks (RSNs) has certain limitations. While k-means is a widely used unsupervised learning technique with diverse applications, it necessitates the predefinition of the number of clusters, is sensitive to the initial placement of cluster centroids, and assumes spherical and equally sized clusters. These assumptions may not always hold for RSNs. To address these issues, we have implemented an adapted version of the k-means clustering algorithm. We utilise the elbow method to define the appropriate number of clusters (k) without making a priori assumptions, and we construct an association matrix based on 200 runs to mitigate the impact of initial conditions.

It is important to note that the k-means clustering algorithm lacks temporal information, as it solely operates on spatial patterns and does not inherently consider the temporal dynamics of RSNs. In contrast, ICA takes into account the dynamics of RSNs, making it a suitable alternative method for further validation. However, our analysis primarily focuses on examining the performance of both models in replicating static FC. Thus, we have chosen a method to derive sub-networks based on static FC patterns, without considering the network dynamics captured by ICA.

### 5.4 Generation of hemodynamic and electrophysiological data

One of the main limitations of our modelling approaches is the fact that, although we employ a generative approach to transition from neuronal activity (e.g. LFPs or population firing rates) to BOLD signals (Buxton et al., 1998; Friston et al., 2000), we do not adopt a similar strategy for generating MEG signals. Instead, we assume that the signals generated by our models can be directly mapped to source-reconstructed MEG. However, the MEG/EEG inverse problem is insoluble, and all source inversion algorithms (beamformers, minimum norm etc.) impose some form of assumption. In the context of fMRI, hemodynamic models reflect the physiological relationship between population activity and the blood oxygenation measured through BOLD signals and those have been extensively validated (Buxton et al., 1998; Friston et al., 2000; Handwerker et al., 2012). Therefore, our results would benefit from a comparable generative model to compute the source dipole currents detected via MEG (Lopes da Silva, 2013). Nonetheless, since both our models can still reveal empirically relevant spatiotemporal patterns of MEG signals in a comparable manner, one might argue that this issue does not undermine our conclusions.

Another relevant point is that, in the context of modelling MEG signals, we did not implement the leakage correction algorithm, mainly to due to the effective absence of source leakage in the models. Previous modelling studies have applied this preprocessing step to simulated data (Abeysuriya et al., 2018; Hadida et al., 2018), particularly when using the Wilson-Cowan model for local dynamics, arguing it ensures comparability between simulated and empirical results - since true zero-lag interactions may also be removed from empirical data. Conversely, other studies based on coupled oscillators did not generally apply orthogonalisation to simulated data ((Cabral et al., 2022, 2014; Deco et al., 2017). That said, each model relies differently on true zero-lag synchronization for the emergence of global dynamics. For the SL model, we generally observe short latency interactions, with a notable decay in the distribution as we move towards longer lags. In contrast, the signals generated with the WC model present a more complex landscape, with a significant incidence of correlations with longer latencies (> 40 ms) and distributions that are either bimodal or long-tailed (Supplementary Figure S18-S19). For this reason, we have opted to not perform leakage correction on simulated data to not affect the comparability of both models used in this work. Nonetheless, we suggest that future modelling endeavours should investigate the role of zero-lag interactions in supporting network connectivity and dynamics of different modelling approaches.

### 5.5 Subcortical structures

In this work, network dynamics are modelled without accounting for the influence of subcortical nodes. The first reason is the inadequate subcortical resolution offered by common atlases used in our modelling (i.e., AAL, Schaefer, Desikan-Killiany). The second is related to the difficulty in modelling the dynamics of some subcortical structures using the SL and WC models, which either consider nodes as oscillators or as networks of reciprocally coupled excitatory and inhibitory neurons, suitable for cortical dynamics. While this approach could still be valid for structures such as the hippocampus (Kandel, 2021), it would fail to accurately represent the dynamics of areas such as the striatum, which is mainly composed of inhibitory neurons (Lanciego et al., 2012), or the cerebellum, which has a distinct microcircuitry (Voogd and Glickstein, 1998). The omission of subcortical structures could impact our results, for example by disregarding the influence of widespread thalamocortical projections in the establishment of alpha rhythms (Halgren et al., 2019; Roux et al., 2013) and in supporting interhemispheric connectivity (Teipel et al., 2009; Wang et al., 2019). Nonetheless, such approaches would require more complex models with multilevel structures (Meier et al., 2022). See (van Wijk et al., 2018), for a fuller discussion of this issue in neural mass modelling.

### 5.6 E-I homoeostasis

Regarding the implementation of E-I homoeostasis, we modelled E-I balance through inhibitory plasticity (Abeysuriya et al., 2018; Deco et al., 2019; Deco et al., 2021; Vogels et al., 2011). While research shows the importance of inhibitory connections for the maintenance of balance (Luz and Shamir, 2012; Vogels et al., 2013, 2011), there are other mechanisms in place such as scaling of recurrent excitation (Turrigiano et al., 1998) or regulation of intrinsic excitability of excitatory populations (Desai et al., 1999), which have not yet been explored in large-scale models. While the excitatory and inhibitory time constants determine the oscillatory dynamics of WC nodes (see *Neural mass model,* Methods), changes in local inhibition might further affect local dynamics, especially in highly connected nodes, which require stronger local inhibition. Therefore, including additional homoeostasis mechanisms, that synergistically interact with each other, may reveal relevant patterns of local microcircuitry, possibly related to gradients in cortical organisation (Wang, 2020). That said, still on the topic of regional heterogeneity, we stress that we implemented a universal target firing rate, following (Abeysuriya et al., 2018; Deco et al., 2014; Hellyer et al., 2016). However, it is possible that regional heterogeneities are not limited to the microstructure of cortical networks. While studies show that the cortex homeostatically tunes toward criticality in visual areas (Ma et al., 2019), it is possible that dynamics are adjusted differently across the cortex, especially in the higher hierarchical levels (Felleman and Van Essen, 1991; Wang, 2020). Consequently, future models should explore the possibility of heterogeneity in target firing rates, especially as it pertains to the cortical hierarchy.

### 5.7 Model optimisation

On a more methodological level, the use of a grid search for model optimisation, despite being common in large-scale modelling research (Cabral et al., 2022; Deco et al., 2017; Hellyer et al., 2016), is an inefficient method to explore the parameter space. This can be solved by making use of recent advances such as Bayesian optimisation (Hadida et al., 2018) and corresponding variational procedures used in dynamic causal modelling (Frässle et al., 2017; Razi et al., 2017). In addition, different metrics of performance could have been used to compare empirical and simulated data, such as power-spectrum similarity (Verma et al., 2022), and distance measures such as KL-divergence, KS-distance or mean-squared error between matrices (Savva et al., 2019).

### 5.8 Relationship between BOLD and MEG signals

One of the main perspectives offered by exploring the model performance across modalities is the fact that our models can generate simultaneous MEG and BOLD signals. This is relevant, given that the relationship between MEG and fMRI signals is not yet fully understood (Garcés et al., 2016; Hall et al., 2014). In addition, recent results suggest that this relationship is not homogeneous across the brain, and that it is driven by differences in local circuitry related to the cortical hierarchy (Shafiei et al., 2022). Therefore, multimodal models might help elucidate the interactions between the processes behind the two signals, particularly with studies involving the perturbation of dynamics with external currents. We propose future studies to focus on the mechanistic relationship between MEG and fMRI, and how MEG features such as the relative power at different frequency bands, cross-frequency interactions and synchronisation can reflect the properties of hemodynamic signals. Please see (Friston et al., 2019; Goldman et al., 2002; Jafarian et al., 2020; Wei et al., 2020) for further discussion.

### 5.9 Model augmentation with heterogeneity

Given our conclusions on the constraints imposed by the connectome in both models we explored, a crucial future step in modelling research is the inclusion of empirically derived sources of heterogeneity in large-scale computational models. Recent endeavours have shown the use of including transcriptomically derived differences in the excitability of local populations in the representation of static (Demirtaş et al., 2019) and dynamic (Deco et al., 2021) features of large-scale brain activity. In addition, results suggest that the variations in structure-function coupling across the cortical hierarchy are shaped by heterogeneities in local E-I balance and myelination levels (Fotiadis et al., 2022), or in cortico-subcortical interactions in terms of neuroreceptors density maps (Beliveau et al., 2017), temporal time-scales (Baldassano et al., 2017), gene expression (Hawrylycz et al., 2012), myelin content (in terms of T1/T2-weighted MRI signal) (Glasser and Van Essen, 2011) and functional connectivity (Kong et al., 2021) - offering further explanations as to why empirical FC exhibits characteristics that cannot be explained solely by SC. Therefore, we believe that it is essential for further modelling studies to make use of multilevel datasets (Arnatkevic̆iūtė et al., 2019; Royer et al., 2022) to constrain models with directed connectivity that define cortical hierarchies (Deco et al., 2021). In addition, future avenues for research should also consider that functional interactions in the neocortex are shaped by interactions with subcortical structures, such as the thalamus (Proske et al., 2011), or neuromodulatory systems that control effective interactions between cortical neuronal populations (Amil and Verschure, 2021).

## 6 Conclusion

In this study, we compared the performance of two large-scale models, the Wilson-Cowan (WC) and Stuart-Landau (SL) models, in explaining multiresolution empirical-like functional connectivity, functional connectivity dynamics, and metastable oscillatory modes. Our results suggest that delays have distinct effects on the models' ability to reproduce these features. When assessing cross-feature performance, the WC model can approximate all features with short delays and moderate to high coupling, while the SL model is unable to attain comparable performance across all features. In terms of cross-modality performance, both models could explain FC across modalities. However, the WC model has a clear advantage regarding spatiotemporal and oscillatory dynamics, highlighting the importance of E-I balance, which underwrites the recruitment of local dynamics and propagation of relevant spatiotemporal patterns of network activity. When assessing the sub-networks, we observed varying levels of performance depending on the network and modality of interest. The limitations observed in the reproduction of empirical FC and associated sub-networks may be due to excessive structural constraints: while the WC model reflects the underlying weight and distance dependence of SC, the SL model is mainly constrained by node communicability. We argue that adding sources of local heterogeneity might contribute to the emergence of the functional networks observed in empirical data. In conclusion, we have demonstrated how the interaction between local dynamics (E-I balance), network properties (conduction delays) and the underlying structural framework shape network interactions and dynamics of the neocortex. Furthermore, we highlight the strengths and limitations of current modelling approaches in studying the defining principles of large-scale brain dynamics.

## CRediT authorship contribution statement

**Francesca Castaldo:** Conceptualization, Methodology, Investigation, Visualization, Project administration, Writing – original draft, Writing – review & editing. **Francisco Páscoa dos Santos:** Conceptualization, Methodology, Investigation, Visualization, Writing – original draft, Writing – review & editing. **Ryan C Timms:** Conceptualization, Methodology. **Joana Cabral:** Conceptualization, Methodology, Supervision, Writing – review & editing. **Jakub Vohryzek:** Methodology, Writing – review & editing. **Gustavo Deco:** Funding acquisition, Supervision. **Mark Woolrich:** Methodology. **Karl Friston:** Funding acquisition, Supervision, Writing – review & editing. **Paul Verschure:** Funding acquisition, Supervision. **Vladimir Litvak:** Conceptualization, Methodology, Funding acquisition, Project administration, Supervision, Writing – review & editing.

## Declaration of Competing Interest

Authors declare that they have no competing interests.

## Data Availability

All simulations and analysis were performed in Python except for the Source Reconstruction algorithm which was performed in MATLAB2021b. The codes and materials used in this study are available at: https://gitlab.com/francpsantos/whole_brain_generative_models.

## Appendix Supplementary materials

Image, application 1

## References

[bib0001] Abeysuriya R.G., Hadida J., Sotiropoulos S.N., Jbabdi S., Becker R., Hunt B.A.E., Brookes M.J., Woolrich M.W. (2018). A biophysical model of dynamic balancing of excitation and inhibition in fast oscillatory large-scale networks. PLoS Comput. Biol..

[bib0002] Ahlfors S.P., Han J., Belliveau J.W., Hämäläinen M.S. (2010). Sensitivity of MEG and EEG to source orientation. Brain Topogr..

[bib0003] Alexander A.L., Lee J.E., Lazar M., Field A.S. (2007). Diffusion tensor imaging of the brain. Neurotherapeutics.

[bib0004] Amil A.F., Verschure P.F.M.J. (2021). Supercritical dynamics at the edge-of-chaos underlies optimal decision-making. J. Phys.: Complexity.

[bib0005] Andronov A.A., Vitt A.A., Khaǐkin S.E (1966). https://books.google.es/books?id=PfkAtAEACAAJ.

[bib0006] Arnatkevic̆iūtė A., Fulcher B.D., Fornito A. (2019). A practical guide to linking brain-wide gene expression and neuroimaging data. Neuroimage.

[bib0007] Ashburner J., Friston K.J. (2005). Unified segmentation. Neuroimage.

[bib0008] Atasoy S., Donnelly I., Pearson J. (2016). Human brain networks function in connectome-specific harmonic waves. Nat. Commun..

[bib0009] Baker A.P., Brookes M.J., Rezek I.A., Smith S.M., Behrens T., Probert Smith P.J., Woolrich M (2014). Fast transient networks in spontaneous human brain activity. Elife.

[bib0010] Baldassano C., Chen J., Zadbood A., Pillow J.W., Hasson U., Norman K.A. (2017). Discovering event structure in continuous narrative perception and memory. Neuron.

[bib0011] Beggs J., Timme N. (2012). Being Critical of Criticality in the Brain [Review]. Front. Physiol..

[bib0012] Beliveau V., Ganz M., Feng L., Ozenne B., Højgaard L., Fisher P.M., Svarer C., Greve D.N., Knudsen G.M. (2017). A high-resolution in vivo atlas of the human brain's serotonin system. J. Neurosci..

[bib0013] Beurle R.L., Matthews B.H.C. (1956). Properties of a mass of cells capable of regenerating pulses. Philos. Trans. R. Soc. Lond., B, Biol. Sci..

[bib0014] Bick C., Goodfellow M., Laing C.R., Martens E.A. (2020). Understanding the dynamics of biological and neural oscillator networks through exact mean-field reductions: a review. J. Mathe. Neurosci..

[bib0015] Bonkhoff A.K., Schirmer M.D., Bretzner M., Etherton M., Donahue K., Tuozzo C., Nardin M., Giese A.K., Wu O., V D.C., Grefkes C., Rost N.S (2021). Abnormal dynamic functional connectivity is linked to recovery after acute ischemic stroke. Hum. Brain Mapp..

[bib0016] Boshkovski T., Kocarev L., Cohen-Adad J., Mišić B., Lehéricy S., Stikov N., Mancini M. (2021). The R1-weighted connectome: complementing brain networks with a myelin-sensitive measure. Network Neurosci..

[bib0017] Boto E., Hill R.M., Rea M., Holmes N., Seedat Z.A., Leggett J., Shah V., Osborne J., Bowtell R., Brookes M.J. (2021). Measuring functional connectivity with wearable MEG. Neuroimage.

[bib0018] Breakspear M. (2017). Dynamic models of large-scale brain activity. Nat. Neurosci..

[bib0019] Brookes M.J., Hale J.R., Zumer J.M., Stevenson C.M., Francis S.T., Barnes G.R., Owen J.P., Morris P.G., Nagarajan S.S. (2011). Measuring functional connectivity using MEG: methodology and comparison with fcMRI. Neuroimage.

[bib0020] Brookes M.J., Woolrich M., Luckhoo H., Price D., Hale J.R., Stephenson M.C., Barnes G.R., Smith S.M., Morris P.G. (2011). Investigating the electrophysiological basis of resting state networks using magnetoencephalography. Proceed. Nat. Acad. Sci..

[bib0021] Buxton R.B., Wong E.C., Frank L.R. (1998). Dynamics of blood flow and oxygenation changes during brain activation: the balloon model. Magn. Reson. Med..

[bib0022] Buzsáki G. (2006).

[bib0023] Buzsáki G., Wang X.J. (2012). Mechanisms of gamma oscillations. Annu. Rev. Neurosci..

[bib0024] Cabral J., Castaldo F., Vohryzek J., Litvak V., Bick C., Lambiotte R., Friston K., Kringelbach M.L., Deco G. (2022). Metastable oscillatory modes emerge from synchronization in the brain spacetime connectome. Commun. Phys..

[bib0025] Cabral J., Hugues E., Sporns O., Deco G. (2011). Role of local network oscillations in resting-state functional connectivity. Neuroimage.

[bib0027] Cabral J., Luckhoo H., Woolrich M., Joensson M., Mohseni H., Baker A., Kringelbach M.L., Deco G. (2014). Exploring mechanisms of spontaneous functional connectivity in MEG: how delayed network interactions lead to structured amplitude envelopes of band-pass filtered oscillations. Neuroimage.

[bib0045] Cabral J., Kringelbach M.L., Deco J. (2017). Functional connectivity dynamically evolves on multiple time-scales over a static structural connectome: Models and mechanisms. NeuroImage.

[bib0028] Cabral J., Vidaurre D., Marques P., Magalhães R., Silva Moreira P., Miguel Soares J., Deco G., Sousa N., Kringelbach M.L (2017). Cognitive performance in healthy older adults relates to spontaneous switching between states of functional connectivity during rest. Sci. Rep..

[bib0029] Cheyne D., Bostan A.C., Gaetz W., Pang E.W. (2007). Event-related beamforming: a robust method for presurgical functional mapping using MEG. Clin. Neurophysiol..

[bib0030] Choe C.-U., Dahms T., Hövel P., Schöll E. (2010). Controlling synchrony by delay coupling in networks: from in-phase to splay and cluster states. Phys. Rev. E.

[bib0031] Cocchi L., Gollo L.L., Zalesky A., Breakspear M. (2017). Criticality in the brain: a synthesis of neurobiology, models and cognition. Prog. Neurobiol..

[bib0032] Cofré R., Herzog R., Mediano P.A.M., Piccinini J., Rosas F.E., Sanz Perl Y., Tagliazucchi E. (2020). Whole-brain models to explore altered states of consciousness from the bottom up. Brain Sci..

[bib0033] Colclough G.L., Brookes M.J., Smith S.M., Woolrich M.W. (2015). A symmetric multivariate leakage correction for MEG connectomes. Neuroimage.

[bib0034] Conturo T.E., Lori N.F., Cull T.S., Akbudak E., Snyder A.Z., Shimony J.S., McKinstry R.C., Burton H., Raichle M.E. (1999). Tracking neuronal fiber pathways in the living human brain. Proc. Natl. Acad. Sci. U. S. A..

[bib0035] Coombes S. (2005). Waves, bumps, and patterns in neural field theories. Biol. Cybern..

[bib0036] Damoiseaux J.S., Greicius M.D. (2009). Greater than the sum of its parts: a review of studies combining structural connectivity and resting-state functional connectivity. Brain Struct. Funct..

[bib0037] David O., Kiebel S.J., Harrison L.M., Mattout J., Kilner J.M., Friston K.J. (2006). Dynamic causal modeling of evoked responses in EEG and MEG. Neuroimage.

[bib0038] Deco G., Cabral J., Woolrich M.W., Stevner A.B., van Hartevelt T.J., Kringelbach M.L. (2017). Single or multi-frequency generators in on-going brain activity: a mechanistic whole-brain model of empirical MEG data. Neuroimage.

[bib0039] Deco G., Cruzat J., Kringelbach M.L. (2019). Brain songs framework used for discovering the relevant timescale of the human brain. Nat. Commun..

[bib0040] Deco G., Jirsa V., McIntosh A.R., Sporns O., Kotter R. (2009). Key role of coupling, delay, and noise in resting brain fluctuations. Proc. Natl. Acad. Sci. U. S. A..

[bib0041] Deco G., Jirsa V., McIntosh A.R., Sporns O., Kötter R. (2009). Key role of coupling, delay, and noise in resting brain fluctuations. Proc. Natl. Acad. Sci. U. S. A..

[bib0042] Deco G., Jirsa V.K., McIntosh A.R. (2013). Resting brains never rest: computational insights into potential cognitive architectures. Trends Neurosci..

[bib0043] Deco G., Jirsa V.K., Robinson P.A., Breakspear M., Friston K. (2008). The dynamic brain: from spiking neurons to neural masses and cortical fields. PLoS Comput. Biol..

[bib0044] Deco G., Kringelbach M.L., Arnatkeviciute A., Oldham S., Sabaroedin K., Rogasch N.C., Aquino K.M., Fornito A. (2021). Dynamical consequences of regional heterogeneity in the brain's transcriptional landscape. Sci. Adv..

[bib0046] Deco G., McIntosh A.R., Shen K., Hutchison R.M., Menon R.S., Everling S., Hagmann P., Jirsa V.K. (2014). Identification of optimal structural connectivity using functional connectivity and neural modeling. J. Neurosci..

[bib0047] Deco G., Perl Y.S., Vuust P., Tagliazucchi E., Kennedy H., Kringelbach M.L. (2021). Rare long-range cortical connections enhance human information processing. Current Biology.

[bib0048] Deco G., Ponce-Alvarez A., Hagmann P., Romani G.L., Mantini D., Corbetta M. (2014). How local excitation-inhibition ratio impacts the whole brain dynamics. J. Neurosci..

[bib0049] Deco G., Ponce-Alvarez A., Mantini D., Romani G.L., Hagmann P., Corbetta M. (2013). Resting-state functional connectivity emerges from structurally and dynamically shaped slow linear fluctuations. J. Neurosci..

[bib0050] Deco G., Vidaurre D., Kringelbach M.L. (2021). Revisiting the global workspace orchestrating the hierarchical organization of the human brain. Nat. Hum. Behav..

[bib0051] Dehghani N., Peyrache A., Telenczuk B., Le Van Quyen M., Halgren E., Cash S.S., Hatsopoulos N.G., Destexhe A. (2016). Dynamic balance of excitation and inhibition in human and monkey neocortex. Sci. Rep..

[bib0052] Demirtaş M., Burt J.B., Helmer M., Ji J.L., Adkinson B.D., Glasser M.F., Van Essen D.C., Sotiropoulos S.N., Anticevic A., Murray J.D. (2019). Hierarchical heterogeneity across human cortex shapes large-scale neural dynamics. Neuron.

[bib0053] Desai N.S., Rutherford L.C., Turrigiano G.G. (1999). Plasticity in the intrinsic excitability of cortical pyramidal neurons. Nat. Neurosci..

[bib0054] Ercsey-Ravasz M., Markov N.T., Lamy C., Van Essen D.C., Knoblauch K., Toroczkai Z., Kennedy H. (2013). A predictive network model of cerebral cortical connectivity based on a distance rule. Neuron.

[bib0055] Estrada E., Hatano N., Benzi M. (2012). The physics of communicability in complex networks. Phys. Rep..

[bib0056] Felleman D.J., Van Essen D.C. (1991). Distributed hierarchical processing in the primate cerebral cortex. Cereb. Cortex.

[bib0057] Filippi M., Spinelli E.G., Cividini C., Agosta F. (2019). Resting state dynamic functional connectivity in neurodegenerative conditions: a review of magnetic resonance imaging findings [Mini Review]. Front. Neurosci..

[bib0058] Chapter 7 - paths, diffusion, and navigation, Fornito A., Zalesky A., Bullmore E.T. (2016). Fundamentals of Brain Network Analysis.

[bib0059] Fotiadis, P., Cieslak, M., He, X., Caciagli, L., Ouellet, M., Satterthwaite, T.D., Shinohara, R.T., & Bassett, D.S. (2022). Myelination and excitation-inhibition balance synergistically shape structure-function coupling across the human cortex. *bioRxiv*, 2022.2010.2020.512802. doi:10.1101/2022.10.20.512802. PMC10542365 37777569

[bib0060] Fox M.D., Corbetta M., Snyder A.Z., Vincent J.L., Raichle M.E. (2006). Spontaneous neuronal activity distinguishes human dorsal and ventral attention systems. Proceed. Nat. Acad. Sci..

[bib0061] Frässle S., Lomakina E.I., Razi A., Friston K.J., Buhmann J.M., Stephan K.E. (2017). Regression DCM for fMRI. Neuroimage.

[bib0062] Friston K., Harrison L., Daunizeau J., Kiebel S., Phillips C., Trujillo-Barreto N., Henson R., Flandin G., Mattout J. (2008). Multiple sparse priors for the M/EEG inverse problem. Neuroimage.

[bib0063] Friston K.J. (1994). Functional and effective connectivity in neuroimaging: a synthesis. Hum. Brain Mapp..

[bib0064] Friston K.J. (1997). Transients, metastability, and neuronal dynamics. Neuroimage.

[bib0065] Friston K.J. (2000). The labile brain. I. Neuronal transients and nonlinear coupling. Philos. Trans. R. Soc. Lond. B Biol. Sci..

[bib0066] Friston K.J., Harrison L., Penny W. (2003). Dynamic causal modelling. Neuroimage.

[bib0067] Friston K.J., Mechelli A., Turner R., Price C.J. (2000). Nonlinear responses in fMRI: the balloon model, volterra kernels, and other hemodynamics. Neuroimage.

[bib0068] Friston K.J., Preller K.H., Mathys C., Cagnan H., Heinzle J., Razi A., Zeidman P. (2019). Dynamic causal modelling revisited. Neuroimage.

[bib0069] Froemke R.C., Merzenich M.M., Schreiner C.E. (2007). A synaptic memory trace for cortical receptive field plasticity. Nature.

[bib0070] Froudist-Walsh S., Bliss D.P., Ding X., Rapan L., Niu M., Knoblauch K., Zilles K., Kennedy H., Palomero-Gallagher N., Wang X.-J. (2021). A dopamine gradient controls access to distributed working memory in the large-scale monkey cortex. Neuron.

[bib0071] Garcés P., Pereda E., Hernández-Tamames J.A., Del-Pozo F., Maestú F., Ángel Pineda-Pardo J. (2016). Multimodal description of whole brain connectivity: a comparison of resting state MEG, fMRI, and DWI. Hum. Brain Mapp..

[bib0072] Glasser M.F., Coalson T.S., Robinson E.C., Hacker C.D., Harwell J., Yacoub E., Ugurbil K., Andersson J., Beckmann C.F., Jenkinson M., Smith S.M., Van Essen D.C. (2016). A multi-modal parcellation of human cerebral cortex. Nature.

[bib0073] Glasser M.F., Smith S.M., Marcus D.S., Andersson J.L., Auerbach E.J., Behrens T.E., Coalson T.S., Harms M.P., Jenkinson M., Moeller S., Robinson E.C., Sotiropoulos S.N., Xu J., Yacoub E., Ugurbil K., Van Essen D.C. (2016). The human connectome project's neuroimaging approach. Nat. Neurosci..

[bib0074] Glasser M.F., Van Essen D.C. (2011). Mapping human cortical areas in vivo based on myelin content as revealed by T1-and T2-weighted MRI. J. Neurosci..

[bib0075] Glomb K., Cabral J., Cattani A., Mazzoni A., Raj A., Franceschiello B. (2022). Computational models in electroencephalography. Brain Topogr..

[bib0076] Godfrey M., Singh K.D. (2021). Measuring robust functional connectivity from resting-state MEG using amplitude and entropy correlation across frequency bands and temporal scales. Neuroimage.

[bib0077] Goldman R.I., Stern J.M., Engel J., Cohen M.S (2002). Simultaneous EEG and fMRI of the alpha rhythm. Neuroreport.

[bib0078] Golland Y., Golland P., Bentin S., Malach R. (2008). Data-driven clustering reveals a fundamental subdivision of the human cortex into two global systems. Neuropsychologia.

[bib0079] Greicius M.D., Krasnow B., Reiss A.L., Menon V. (2003). Functional connectivity in the resting brain: a network analysis of the default mode hypothesis. Proceed. Nat. Acad. Sci..

[bib0080] Hadida J., Sotiropoulos S.N., Abeysuriya R.G., Woolrich M.W., Jbabdi S. (2018). Bayesian Optimisation of Large-Scale Biophysical Networks. Neuroimage.

[bib0081] Hagmann P., Cammoun L., Gigandet X., Meuli R., Honey C.J., Wedeen V.J., Sporns O. (2008). Mapping the Structural Core of Human Cerebral Cortex. PLoS Biol..

[bib0082] Halgren M., Ulbert I., Bastuji H., Fabó D., Erőss L., Rey M., Devinsky O., Doyle W.K., Mak-McCully R., Halgren E., Wittner L., Chauvel P., Heit G., Eskandar E., Mandell A., Cash S.S. (2019). The generation and propagation of the human alpha rhythm. Proceed. Nat. Acad. Sci..

[bib0083] Hall E.L., Robson S.E., Morris P.G., Brookes M.J. (2014). The relationship between MEG and fMRI. Neuroimage.

[bib0084] Hämäläinen M., Hari R., Ilmoniemi R.J., Knuutila J., Lounasmaa O.V. (1993). Magnetoencephalography—theory, instrumentation, and applications to noninvasive studies of the working human brain. Rev. Mod. Phys..

[bib0085] Handwerker D.A., Gonzalez-Castillo J., D'Esposito M., Bandettini P.A. (2012). The continuing challenge of understanding and modeling hemodynamic variation in fMRI. Neuroimage.

[bib0086] Harris Julia J., Jolivet R., Attwell D. (2012). Synaptic energy use and supply. Neuron.

[bib0087] Hawrylycz M.J., Lein E.S., Guillozet-Bongaarts A.L., Shen E.H., Ng L., Miller J.A., Van De Lagemaat L.N., Smith K.A., Ebbert A., Riley Z.L. (2012). An anatomically comprehensive atlas of the adult human brain transcriptome. Nature.

[bib0088] Hellyer P.J., Jachs B., Clopath C., Leech R. (2016). Local inhibitory plasticity tunes macroscopic brain dynamics and allows the emergence of functional brain networks. Neuroimage.

[bib0089] Hermundstad A.M., Brown K.S., Bassett D.S., Aminoff E.M., Frithsen A., Johnson A., Tipper C.M., Miller M.B., Grafton S.T., Carlson J.M. (2014). Structurally-constrained relationships between cognitive states in the human brain. PLoS Comput. Biol..

[bib0090] Hillman E.M. (2014). Coupling mechanism and significance of the BOLD signal: a status report. Annu. Rev. Neurosci..

[bib0091] Hindriks R., Tewarie P.K.B. (2023). Dissociation between phase and power correlation networks in the human brain is driven by co-occurrent bursts. Commun. Biol..

[bib0092] Hipp J.F., Hawellek D.J., Corbetta M., Siegel M., Engel A.K. (2012). Large-scale cortical correlation structure of spontaneous oscillatory activity. Nat. Neurosci..

[bib0093] Honey C.J., Kotter R., Breakspear M., Sporns O. (2007). Network structure of cerebral cortex shapes functional connectivity on multiple time scales. Proc. Natl. Acad. Sci. U. S. A..

[bib0094] Honey C.J., Thivierge J.-P., Sporns O. (2010). Can structure predict function in the human brain?. Neuroimage.

[bib0095] Horn A., Blankenburg F. (2016). Toward a standardized structural–functional group connectome in MNI space. Neuroimage.

[bib0096] Hutchison R.M., Womelsdorf T., Allen E.A., Bandettini P.A., Calhoun V.D., Corbetta M., Della Penna S., Duyn J.H., Glover G.H., Gonzalez-Castillo J. (2013). Dynamic functional connectivity: promise, issues, and interpretations. Neuroimage.

[bib0097] Jafarian A., Litvak V., Cagnan H., Friston K.J., Zeidman P. (2020). Comparing dynamic causal models of neurovascular coupling with fMRI and EEG/MEG. Neuroimage.

[bib0098] Jia X., Siegle J.H., Durand S., Heller G., Ramirez T.K., Koch C., Olsen S.R. (2022). Multi-regional module-based signal transmission in mouse visual cortex. Neuron.

[bib0099] Kandel E.R. (2021).

[bib0100] Kong X., Kong R., Orban C., Wang P., Zhang S., Anderson K., Holmes A., Murray J.D., Deco G., van den Heuvel M (2021). Sensory-motor cortices shape functional connectivity dynamics in the human brain. Nat. Commun..

[bib0101] Kuramoto Y. (1975). International symposium on mathematical problems in theoretical physics.

[bib0102] Lanciego J.L., Luquin N., Obeso J.A. (2012). Functional neuroanatomy of the basal ganglia. Cold Spring Harb. Perspect. Med..

[bib0103] Landau I.D., Egger R., Dercksen V.J., Oberlaender M., Sompolinsky H. (2016). The Impact of Structural Heterogeneity on Excitation-Inhibition Balance in Cortical Networks. Neuron.

[bib0104] Lee M.H., Hacker C.D., Snyder A.Z., Corbetta M., Zhang D., Leuthardt E.C., Shimony J.S. (2012). Clustering of Resting State Networks. PLoS ONE.

[bib0105] Lee M.H., Smyser C.D., Shimony J.S. (2013). Resting-state fMRI: a review of methods and clinical applications. AJNR Am. J. Neuroradiol..

[bib0106] Lee W.S., Ott E., Antonsen T.M. (2009). Large coupled oscillator systems with heterogeneous interaction delays. Phys. Rev. Lett..

[bib0107] Lewis L.D., Setsompop K., Rosen B.R., Polimeni J.R. (2016). Fast fMRI can detect oscillatory neural activity in humans. Proc. Natl. Acad. Sci. U. S. A..

[bib0108] Litvak V., Eusebio A., Jha A., Oostenveld R., Barnes G.R., Penny W.D., Zrinzo L., Hariz M.I., Limousin P., Friston K.J., Brown P. (2010). Optimized beamforming for simultaneous MEG and intracranial local field potential recordings in deep brain stimulation patients. Neuroimage.

[bib0109] Litwin-Kumar A., Doiron B. (2012). Slow dynamics and high variability in balanced cortical networks with clustered connections. Nat. Neurosci..

[bib0110] Litwin-Kumar A., Doiron B. (2014). Formation and maintenance of neuronal assemblies through synaptic plasticity. Nat. Commun..

[bib0111] Liuzzi L., Gascoyne L.E., Tewarie P.K., Barratt E.L., Boto E., Brookes M.J. (2017). Optimising experimental design for MEG resting state functional connectivity measurement. Neuroimage.

[bib0112] Liuzzi L., Quinn A.J., O'Neill G.C., Woolrich M.W., Brookes M.J., Hillebrand A., Tewarie P (2019). How sensitive are conventional meg functional connectivity metrics with sliding windows to detect genuine fluctuations in dynamic functional connectivity? [Original Research]. Front. Neurosci..

[bib0113] Lombardo D., Cassé-Perrot C., Ranjeva J.P., Le Troter A., Guye M., Wirsich J., Payoux P., Bartrés-Faz D., Bordet R., Richardson J.C., Felician O., Jirsa V., Blin O., Didic M., Battaglia D. (2020). Modular slowing of resting-state dynamic functional connectivity as a marker of cognitive dysfunction induced by sleep deprivation. Neuroimage.

[bib0114] Lopes, R.H., Reid, I., & Hobson, P.R. (2007). The two-dimensional Kolmogorov-Smirnov test.

[bib0115] Lopes da Silva F. (2013). EEG and MEG: relevance to Neuroscience. Neuron.

[bib0116] Lu H., Zuo Y., Gu H., Waltz J.A., Zhan W., Scholl C.A., Rea W., Yang Y., Stein E.A. (2007). Synchronized delta oscillations correlate with the resting-state functional MRI signal. Proc. Natl. Acad. Sci. U. S. A..

[bib0117] Luz Y., Shamir M. (2012). Balancing feed-forward excitation and inhibition via hebbian inhibitory synaptic plasticity. PLoS Comput. Biol..

[bib0118] Ma Z., Turrigiano G.G., Wessel R., Hengen K.B. (2019). Cortical circuit dynamics are homeostatically tuned to criticality in vivo. Neuron.

[bib0119] Maffei A., Turrigiano G.G. (2008). Multiple modes of network homeostasis in visual cortical layer 2/3. J. Neurosci..

[bib0120] Mariño J., Schummers J., Lyon D.C., Schwabe L., Beck O., Wiesing P., Obermayer K., Sur M. (2005). Invariant computations in local cortical networks with balanced excitation and inhibition. Nat. Neurosci..

[bib0121] McCulloch W.S., Pitts W. (1943). A logical calculus of the ideas immanent in nervous activity. Bull. Math. Biophys..

[bib0122] Meier J.M., Perdikis D., Blickensdörfer A., Stefanovski L., Liu Q., Maith O., Dinkelbach H.Ü., Baladron J., Hamker F.H., Ritter P. (2022). Virtual deep brain stimulation: multiscale co-simulation of a spiking basal ganglia model and a whole-brain mean-field model with The Virtual Brain. Exp. Neurol..

[bib0123] Niebur E., Schuster H.G., Kammen D.M. (1991). Collective frequencies and metastability in networks of limit-cycle oscillators with time delay. Phys. Rev. Lett..

[bib0124] Nolte G. (2003). The magnetic lead field theorem in the quasi-static approximation and its use for magnetoencephalography forward calculation in realistic volume conductors. Phys. Med. Biol..

[bib0125] Noori R., Park D., Griffiths J.D., Bells S., Frankland P.W., Mabbott D., Lefebvre J. (2020). Activity-dependent myelination: a glial mechanism of oscillatory self-organization in large-scale brain networks. Proceed. Nat. Acad. Sci..

[bib0126] Owen J.P., Wipf D.P., Attias H.T., Sekihara K., Nagarajan S.S. (2012). Performance evaluation of the Champagne source reconstruction algorithm on simulated and real M/EEG data. Neuroimage.

[bib0127] Pajevic S., Basser P.J., Fields R.D. (2014). Role of myelin plasticity in oscillations and synchrony of neuronal activity. Neuroscience.

[bib0128] Paquola C., Hong S.-J. (2023). The Potential of Myelin-Sensitive Imaging: redefining Spatiotemporal Patterns of Myeloarchitecture. Biol. Psychiatry.

[bib0129] Park S.H., Lefebvre J. (2020). Synchronization and resilience in the Kuramoto white matter network model with adaptive state-dependent delays. J. Math. Neurosci..

[bib0130] Páscoa dos Santos, F., Jakub, V., & Paul, F.M.J.V. (2022). Multiscale effects of excitatory-inhibitory homeostasis in lesioned cortical networks: a computational study. *bioRxiv*, 2022.2011.2023.517696. doi:10.1101/2022.11.23.517696. PMC10355437 37418506

[bib0131] Páscoa Dos Santos F., Verschure P. (2021). Excitatory-Inhibitory Homeostasis and Diaschisis: tying the Local and Global Scales in the Post-stroke Cortex. Front. Syst. Neurosci..

[bib0132] Pathak A., Sharma V., Roy D., Banerjee A. (2022). Biophysical mechanism underlying compensatory preservation of neural synchrony over the adult lifespan. Commun. Biol..

[bib0133] Petkoski S., Jirsa V.K. (2019). Transmission time delays organize the brain network synchronization. Philos. Trans. A Math. Phys. Eng. Sci..

[bib0134] Pikovsky A., Kurths J., Rosenblum M., Kurths J. (2003).

[bib0135] Pikovsky A., Rosenblum M. (2015). Dynamics of globally coupled oscillators: progress and perspectives. Chaos.

[bib0136] Pinotsis D.A., Moran R.J., Friston K.J. (2012). Dynamic causal modeling with neural fields. Neuroimage.

[bib0137] Polverino A., Troisi E., Lopez R., M., Marianna L., Antonella R., Francesca T., Fabio L., Leonardo G., Viktor J., Giuseppe S., Pierpaolo S. (2022). Flexibility of fast brain dynamics and disease severity in amyotrophic lateral sclerosis. Neurology.

[bib0138] Portoles O., Qin Y., Hadida J., Woolrich M., Cao M., van Vugt M. (2022). Modulations of local synchrony over time lead to resting-state functional connectivity in a parsimonious large-scale brain model. PLoS ONE.

[bib0139] Powanwe A.S., Longtin A. (2021). Amplitude-phase description of stochastic neural oscillators across the Hopf bifurcation. Phys. Rev. Res..

[bib0140] Power J.D., Cohen A.L., Nelson S.M., Wig G.S., Barnes K.A., Church J.A., Vogel A.C., Laumann T.O., Miezin F.M., Schlaggar B.L. (2011). Functional network organization of the human brain. Neuron.

[bib0141] Proske H.J., Jeanmonod D., Verschure P.F.M.J (2011). A computational model of thalamocortical dysrhythmia. Eur. J. neurosci..

[bib0142] Puigbò J.Y., Maffei G., Herreros I., Ceresa M., González Ballester M.A., Verschure P (2018). Cholinergic behavior state-dependent mechanisms of neocortical gain control: a neurocomputational study. Mol. Neurobiol..

[bib0143] Quinn A.J., Green G.G.R., Hymers M. (2021). Delineating between-subject heterogeneity in alpha networks with spatio-spectral eigenmodes. Neuroimage.

[bib0144] Rabuffo G., Fousek J., Bernard C., Jirsa V. (2021). Neuronal cascades shape whole-brain functional dynamics at rest. eNeuro.

[bib0145] Raj A., Cai C., Xie X., Palacios E., Owen J., Mukherjee P., Nagarajan S. (2020). Spectral graph theory of brain oscillations. Hum. Brain Mapp..

[bib0146] Raj A., Verma P., Nagarajan S. (2022). Structure-function models of temporal, spatial, and spectral characteristics of non-invasive whole brain functional imaging [Review]. Front. Neurosci..

[bib0147] Ranzenberger L.R., Snyder T. (2022). StatPearls.

[bib0149] Razi A., Seghier M.L., Zhou Y., McColgan P., Zeidman P., Park H.J., Sporns O., Rees G., Friston K.J. (2017). Large-scale DCMs for resting-state fMRI. Netw. Neurosci..

[bib0150] Roberts J.A., Gollo L.L., Abeysuriya R.G., Roberts G., Mitchell P.B., Woolrich M.W., Breakspear M. (2019). Metastable brain waves. Nat. Commun..

[bib0151] Rocha R.P., Koçillari L., Suweis S., Corbetta M., Maritan A. (2018). Homeostatic plasticity and emergence of functional networks in a whole-brain model at criticality. Sci. Rep..

[bib0152] Rössler O.E. (1976). An equation for continuous chaos. Phys. Lett. A.

[bib0153] Roux F., Wibral M., Singer W., Aru J., Uhlhaas P.J. (2013). The phase of thalamic alpha activity modulates cortical gamma-band activity: evidence from resting-state MEG recordings. J. Neurosci..

[bib0154] Rowley C.D., Bazin P.-L., Tardif C.L., Sehmbi M., Hashim E., Zaharieva N., Minuzzi L., Frey B.N., Bock N.A. (2015). Assessing intracortical myelin in the living human brain using myelinated cortical thickness [Original Research]. Front. Neurosci..

[bib0155] Royer J., Rodríguez-Cruces R., Tavakol S., Larivière S., Herholz P., Li Q., Vos de Wael R., Paquola C., Benkarim O., Park B.-y., Lowe A.J., Margulies D., Smallwood J., Bernasconi A., Bernasconi N., Frauscher B., Bernhardt B.C. (2022). An Open MRI dataset for multiscale neuroscience. Sci. Data.

[bib0156] Rubin R., Abbott L.F., Sompolinsky H. (2017). Balanced excitation and inhibition are required for high-capacity, noise-robust neuronal selectivity. Proceed. Nat. Acad. Sci..

[bib0157] Saab A.S., Nave K.-A. (2017). Myelin dynamics: protecting and shaping neuronal functions. Curr. Opin. Neurobiol..

[bib0158] Sanz Leon P., Knock S., Woodman M., Domide L., Mersmann J., McIntosh A., Jirsa V. (2013). The virtual brain: a simulator of primate brain network dynamics [Methods]. Front. Neuroinform..

[bib0159] Savva A.D., Mitsis G.D., Matsopoulos G.K. (2019). Assessment of dynamic functional connectivity in resting-state fMRI using the sliding window technique. Brain Behav..

[bib0160] Schirner M., Kong X., Yeo B.T.T., Deco G., Ritter P. (2022). Dynamic primitives of brain network interaction. Neuroimage.

[bib0161] Sekihara K., Nagarajan S.S., Poeppel D., Marantz A. (2004). Asymptotic SNR of scalar and vector minimum-variance beamformers for neuromagnetic source reconstruction. IEEE Trans. Biomed. Eng..

[bib0162] Shafiei G., Baillet S., Misic B. (2022). Human electromagnetic and haemodynamic networks systematically converge in unimodal cortex and diverge in transmodal cortex. PLoS Biol..

[bib0163] Shimbel A., Rapoport A. (1948). A statistical approach to the theory of the central nervous system. Bull. Math. Biophys..

[bib0164] Sjøgård M., De Tiège X., Mary A., Peigneux P., Goldman S., Nagels G., van Schependom J., Quinn A.J., Woolrich M.W., Wens V. (2019). Do the posterior midline cortices belong to the electrophysiological default-mode network?. Neuroimage.

[bib0165] Sorrentino P., Petkoski S., Sparaco M., Lopez E.T., Signoriello E., Baselice F., Bonavita S., Pirozzi M.A., Quarantelli M., Sorrentino G., Jirsa V. (2022). Whole-brain propagation delays in multiple sclerosis, a combined tractography-magnetoencephalography study. The J. Neurosci..

[bib0166] Sorrentino P., Seguin C., Rucco R., Liparoti M., Troisi Lopez E., Bonavita S., Quarantelli M., Sorrentino G., Jirsa V., Zalesky A. (2021). The structural connectome constrains fast brain dynamics. Elife.

[bib0167] Sporns O., Tononi G., Kotter R. (2005). The human connectome: a structural description of the human brain. PLoS Comput. Biol..

[bib0168] Sprekeler H. (2017). Functional consequences of inhibitory plasticity: homeostasis, the excitation-inhibition balance and beyond. Curr. Opin. Neurobiol..

[bib0169] Sreenivasan, K.R., Strykowski, P.J., & Olinger, D.J. (1987). Hopf bifurcation, landau equation, and vortex shedding behind circular cylinders.

[bib0170] Tao H.W., Poo M.-m. (2005). Activity-Dependent Matching of Excitatory and Inhibitory Inputs during Refinement of Visual Receptive Fields. Neuron.

[bib0171] Teipel S.J., Pogarell O., Meindl T., Dietrich O., Sydykova D., Hunklinger U., Georgii B., Mulert C., Reiser M.F., Möller H.-J., Hampel H. (2009). Regional networks underlying interhemispheric connectivity: an EEG and DTI study in healthy ageing and amnestic mild cognitive impairment. Hum. Brain Mapp..

[bib0172] Tewarie P., Abeysuriya R., Byrne Á., O'Neill G.C., Sotiropoulos S.N., Brookes M.J., Coombes S. (2019). How do spatially distinct frequency specific MEG networks emerge from one underlying structural connectome? The role of the structural eigenmodes. Neuroimage.

[bib0173] Tremblay R., Lee S., Rudy B. (2016). GABAergic interneurons in the neocortex: from cellular properties to circuits. Neuron.

[bib0174] Turrigiano G. (2011). Too many cooks? Intrinsic and synaptic homeostatic mechanisms in cortical circuit refinement. Annu. Rev. Neurosci..

[bib0175] Turrigiano G.G., Leslie K.R., Desai N.S., Rutherford L.C., Nelson S.B. (1998). Activity-dependent scaling of quantal amplitude in neocortical neurons. Nature.

[bib0176] Uttley A.M., Matthews B.H.C. (1955). The probability of neural connexions. Proceed. Royal Society of London. Series B - Biol. Sci..

[bib0177] van Ede F., Quinn A.J., Woolrich M.W., Nobre A.C. (2018). Neural oscillations: sustained rhythms or transient burst-events?. Trends Neurosci..

[bib0178] Van Essen D.C., Smith S.M., Barch D.M., Behrens T.E., Yacoub E., Ugurbil K., Consortium W.U.-M.H. (2013). The WU-Minn human connectome project: an overview. Neuroimage.

[bib0179] Van Veen B.D., van Drongelen W., Yuchtman M., Suzuki A. (1997). Localization of brain electrical activity via linearly constrained minimum variance spatial filtering. IEEE Trans. Biomed. Eng..

[bib0180] van Vreeswijk C., Sompolinsky H. (1996). Chaos in neuronal networks with balanced excitatory and inhibitory activity. Science.

[bib0181] van Vugt B., Dagnino B., Vartak D., Safaai H., Panzeri S., Dehaene S., Roelfsema P.R. (2018). The threshold for conscious report: signal loss and response bias in visual and frontal cortex. Science.

[bib0182] van Wijk B.C.M., Cagnan H., Litvak V., Kühn A.A., Friston K.J. (2018). Generic dynamic causal modelling: an illustrative application to Parkinson's disease. Neuroimage.

[bib0183] Vattikonda A., Surampudi B.R., Banerjee A., Deco G., Roy D. (2016). Does the regulation of local excitation-inhibition balance aid in recovery of functional connectivity? A computational account. Neuroimage.

[bib0184] Verma P., Nagarajan S., Raj A. (2022). Spectral graph theory of brain oscillations–Revisited and improved. Neuroimage.

[bib0185] Vezoli J., Vinck M., Bosman C.A., Bastos A.M., Lewis C.M., Kennedy H., Fries P. (2021). Brain rhythms define distinct interaction networks with differential dependence on anatomy. Neuron.

[bib0186] Vidaurre D., Smith S.M., Woolrich M.W. (2017). Brain network dynamics are hierarchically organized in time. Proceed. Nat. Acad. Sci..

[bib0187] Vincent J.L., Kahn I., Snyder A.Z., Raichle M.E., Buckner R.L. (2008). Evidence for a frontoparietal control system revealed by intrinsic functional connectivity. J. Neurophysiol..

[bib0188] Vogels T., Froemke R., Doyon N., Gilson M., Haas J., Liu R., Maffei A., Miller P., Wierenga C., Woodin M., Zenke F., Sprekeler H. (2013). Inhibitory synaptic plasticity: spike timing-dependence and putative network function [Review]. Front. Neural. Circuits.

[bib0189] Vogels T.P., Sprekeler H., Zenke F., Clopath C., Gerstner W. (2011). Inhibitory plasticity balances excitation and inhibition in sensory pathways and memory networks. Science.

[bib0190] Vohryzek J., Deco G., Cessac B., Kringelbach M.L., Cabral J. (2020). Ghost Attractors in spontaneous brain activity: recurrent excursions into functionally-relevant bold phase-locking states. Front. Syst. Neurosci..

[bib0191] Voogd J., Glickstein M. (1998). The anatomy of the cerebellum. Trends Cogn. Sci. (Regul. Ed.).

[bib0192] Wang X.-J. (2020). Macroscopic gradients of synaptic excitation and inhibition in the neocortex. Nat. rev. neurosci..

[bib0193] Wang X., Leong A.T.L., Chan R.W., Liu Y., Wu E.X. (2019). Thalamic low frequency activity facilitates resting-state cortical interhemispheric MRI functional connectivity. Neuroimage.

[bib0194] Waxman S.G. (1980). Determinants of conduction velocity in myelinated nerve fibers. Muscle Nerve.

[bib0195] Wehr M., Zador A.M. (2003). Balanced inhibition underlies tuning and sharpens spike timing in auditory cortex. Nature.

[bib0196] Wei H., Jafarian A., Zeidman P., Litvak V., Razi A., Hu D., Friston K.J. (2020). Bayesian fusion and multimodal DCM for EEG and fMRI. Neuroimage.

[bib0197] Wens V., Bourguignon M., Goldman S., Marty B., Op de Beeck M., Clumeck C., Mary A., Peigneux P., Van Bogaert P., Brookes M.J., De Tiège X. (2014). Inter- and intra-subject variability of neuromagnetic resting state networks. Brain Topogr..

[bib0198] Wilson H.R., Cowan J.D. (1972). Excitatory and inhibitory interactions in localized populations of model neurons. Biophys. J..

[bib0199] Wyss R., König P., Verschure P.F.M.J. (2006). A model of the ventral visual system based on temporal stability and local memory. PLoS Biol..

[bib0200] Xue M., Atallah B.V., Scanziani M. (2014). Equalizing excitation-inhibition ratios across visual cortical neurons. Nature.

[bib0201] Zalesky A., Fornito A., Harding I.H., Cocchi L., Yücel M., Pantelis C., Bullmore E.T. (2010). Whole-brain anatomical networks: does the choice of nodes matter?. Neuroimage.

