# Supplementary material for: Multi-modal and multi-model interrogation of large-scale functional brain networks
Source: Neuroimage. 2023 Aug 15;277:120236. doi: 10.1016/j.neuroimage.2023.120236 (PMC10958139; doi:10.1016/j.neuroimage.2023.120236)
Supplement: Supplementary file 1 [file mmc1.docx]

# Multi-modal and multi-model interrogation of large-scale functional brain networks

Francesca Castaldo*, Francisco Santos*, Ryan C Timms, Joana Cabral, Jakub Vohryzek, Gustavo Deco, Mark Woolrich, Karl Friston, Paul Verschure, Vladimir Litvak

# **Supplementary Methods**

# Overview

This supplementary document is divided in *five* main sections.

*Section I* explores the node dynamics of the uncoupled Wilson & Cowan (WC) and Stuart Landau (SL) models. The dynamics of each model is perturbed with noise, generating oscillations with varying amplitude and frequency. The section also presents the frequency profile of the WC model at different values of the population time constants.

*Section II* discusses the homeostatic plasticity and the model optimization for evaluating the stability of the local inhibitory weights. The steady-state test condition for c_EI is also explained. Additionally, this section analyses the metastability, synchrony, and peak frequency of the Stuart Landau and Wilson & Cowan models.

*Section III* focuses on the model performance, specifically the role of inhibitory synaptic plasticity, the importance of including a target firing rate, threshold validation for the analysis across features, and metastable oscillatory modes topographies.

*Section IV* discusses Wilson & Cowan and Stuart Landau model performance when given a shuffled input of structural connectivity. The goal of this analysis is to prove the predictive validity of the connectome, as without the right structure, there will be no emergence of functionally relevant patterns. Also, the relationship between FC and three different network measures (namely shortest path length, Euclidean distance, and communicability) is presented for empirical, WC and SL models.

*Section V* introduces the maths behind MEG amplitude envelope correlation and source reconstruction algorithm.

## SECTION I

### Uncoupled Node Dynamics

**
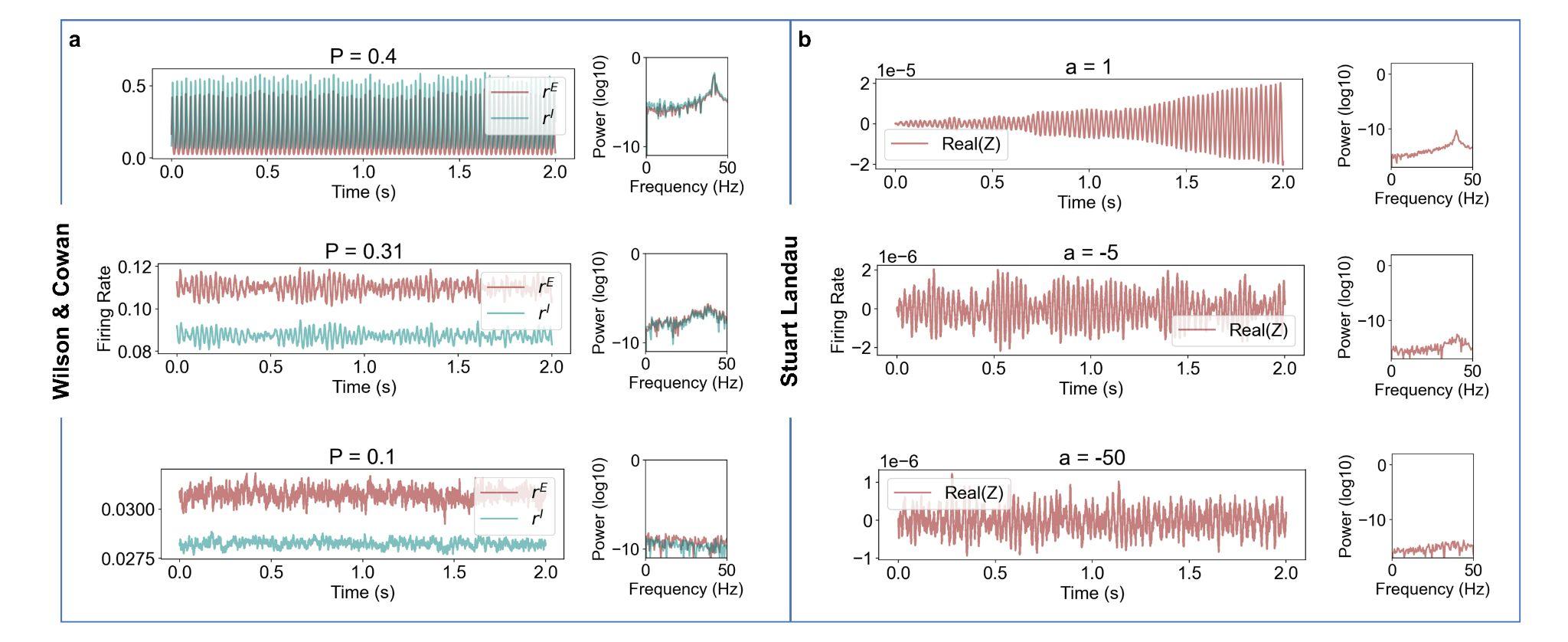
**

Figure S1. Uncoupled node dynamics for the Wilson & Cowan and Stuart Landau models, at different values of the bifurcation parameter and fundamental frequency of 40Hz. Each dynamical Wilson & Cowan (a) and Stuart Landau (b) oscillatory unit is perturbed with noise, where, depending on the value of the bifurcation parameter (*P* and *a* respectively), the model generates a pure oscillatory signal (top), oscillations with fluctuating amplitude (middle), noisy signal (bottom).

**
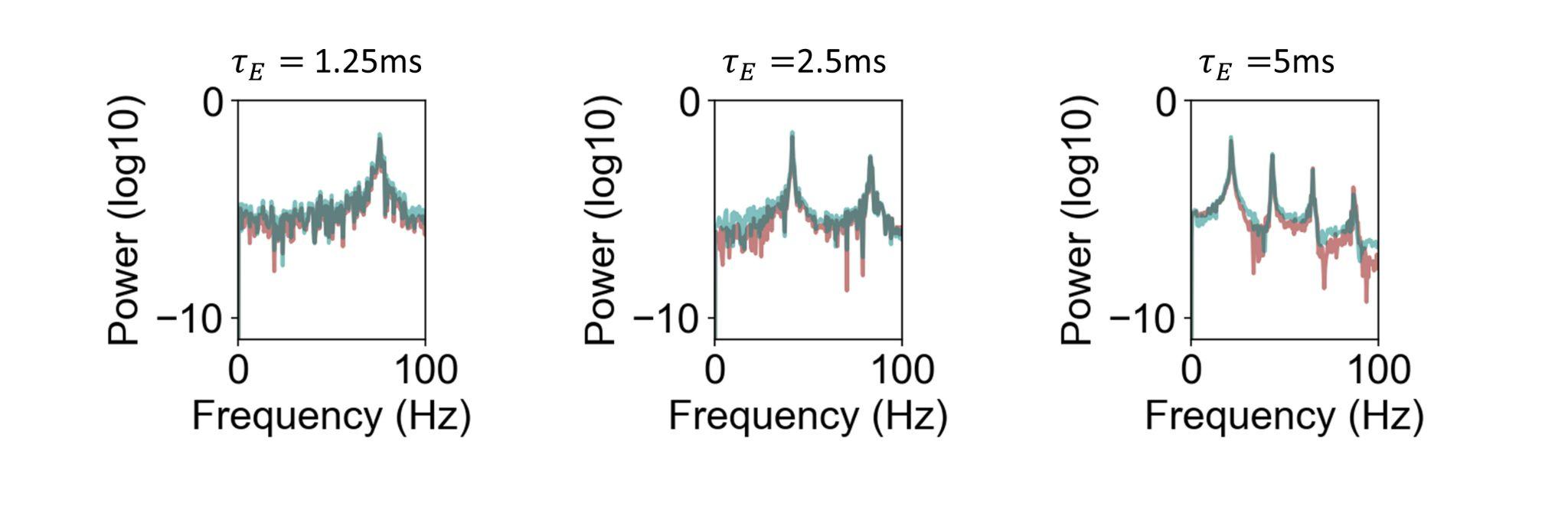
**

Figure S2. Frequency profile for different values of $\boldsymbol{\tau}_{\boldsymbol{E}}$ in the WC model. Effect of the population time constants on the frequency of oscillation, where $\boldsymbol{\tau}_{\boldsymbol{I}}$=$\boldsymbol{2*}\boldsymbol{\tau}_{\boldsymbol{E}}$. In this work we choose $\boldsymbol{\tau}_{\boldsymbol{E}}$ and $\boldsymbol{\tau}_{\boldsymbol{I}}$so that the characteristic frequency of isolated neural masses is within the gamma range (~40 Hz).

## SECTION II

### Homeostatic Plasticity


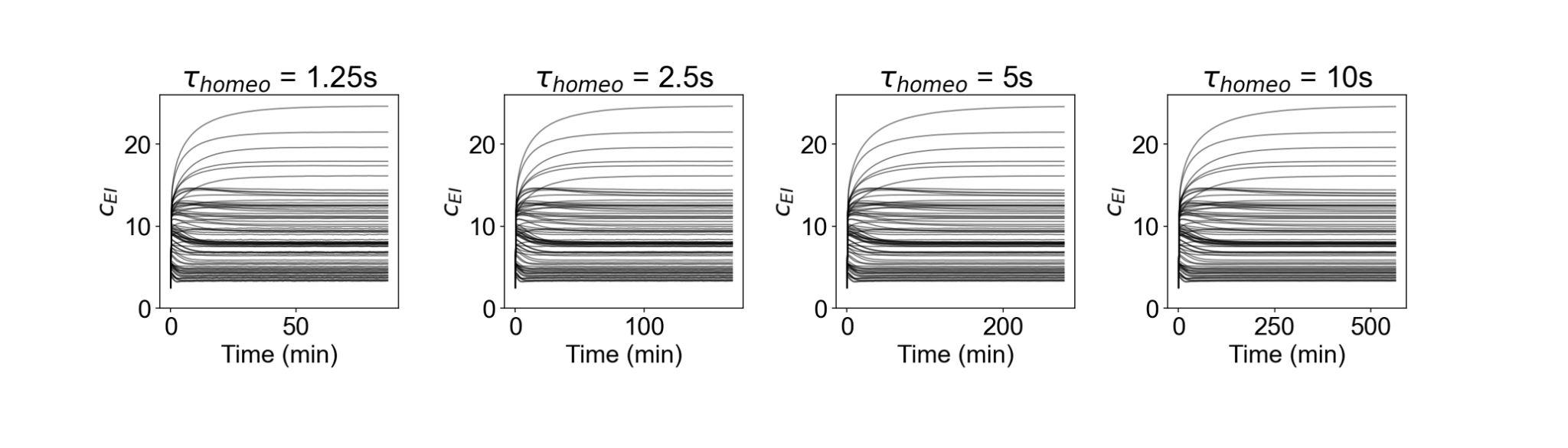


Figure S3. Influence of the homeostatic plasticity on $\boldsymbol{c}_{\boldsymbol{EI}}$. When homeostatic plasticity ($\boldsymbol{\tau}_{\boldsymbol{homeo}}$) is sufficiently slow to be decoupled from fast dynamics of intrinsic oscillations, $\boldsymbol{c}_{\boldsymbol{EI}}$ will reach nearly the same steady state, independently of the time constant.

### Model optimisation: evaluating the stability of the local inhibitory weights.

We record $\boldsymbol{c}_{\boldsymbol{EI}}$ weights every 10s, enough to capture their slow dynamics. We then monitor the evolution of $\boldsymbol{c}_{\boldsymbol{EI}}$and allow simulations to run for either 500 minutes of simulation time or until local weights have converged to a steady state for all network nodes, evaluated through the condition described in Figure S6.

**
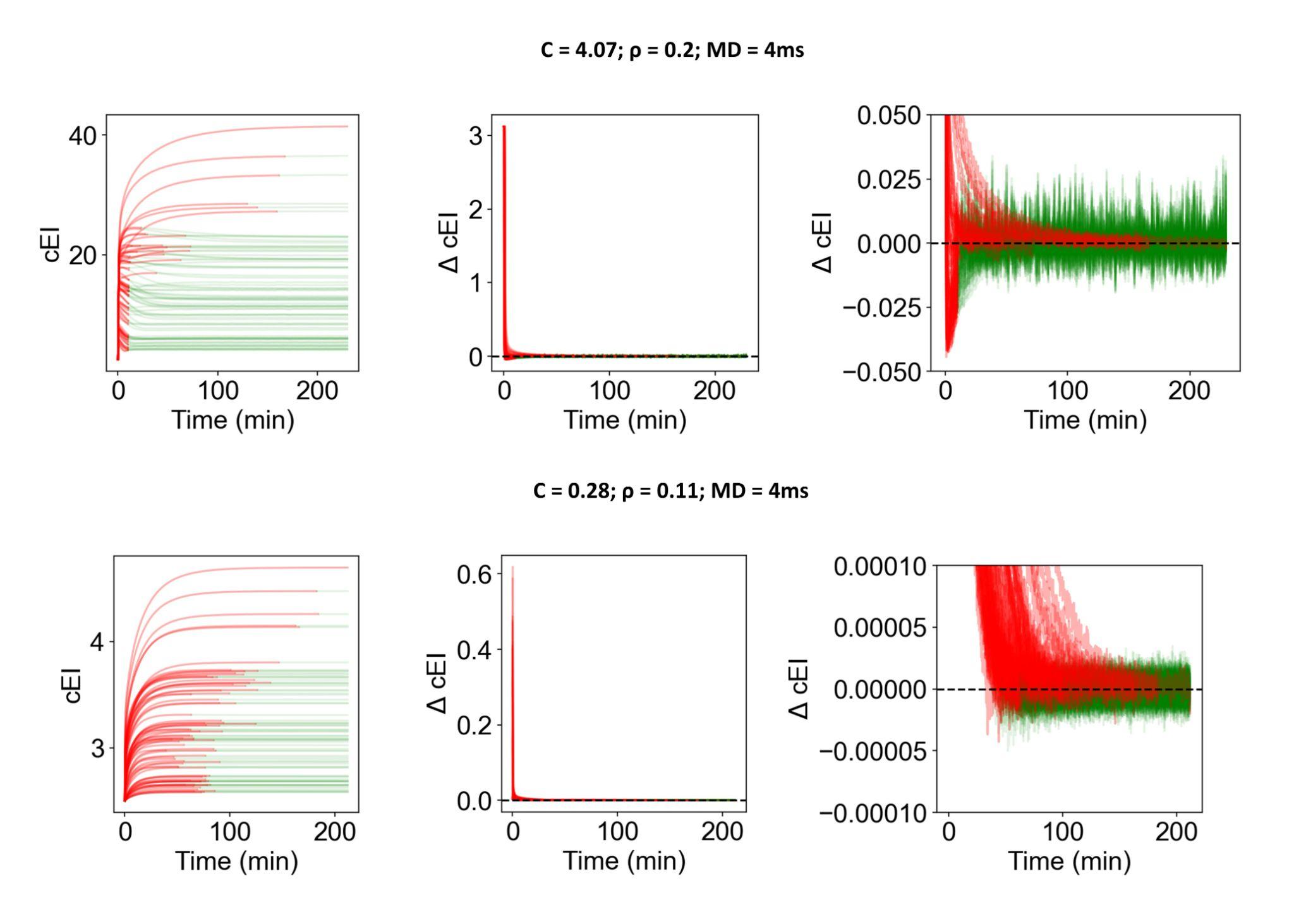
**

Figure S4. Steady state test condition for $\boldsymbol{c}_{\boldsymbol{EI}}$. Every 10 seconds, a vector keeping a down-sampled version of $\boldsymbol{c}_{\boldsymbol{EI}}$ in the last 10 minutes is created for every node as follows: $\boldsymbol{c}_{\boldsymbol{EI,vec}}\boldsymbol{(t)=[}\boldsymbol{c}_{\boldsymbol{EI}}$(t-$\boldsymbol{T}_{\boldsymbol{window}}\boldsymbol{),}\boldsymbol{c}_{\boldsymbol{EI}}\boldsymbol{(t-}\boldsymbol{T}_{\boldsymbol{window}}\boldsymbol{+10}\boldsymbol{s), ... ,}\boldsymbol{c}_{\boldsymbol{EI}}$(t-10s), $\boldsymbol{c}_{\boldsymbol{EI}}$(t)], where $\boldsymbol{T}_{\boldsymbol{window}}\boldsymbol{=600}\boldsymbol{s.}$ Then, The steady-state condition is fulfilled as long as the condition $\boldsymbol{|mean(dc}_{\boldsymbol{EI,vec}}\boldsymbol{)|<}\frac{\boldsymbol{std(}\boldsymbol{dc}_{\boldsymbol{EI,vec}}\boldsymbol{)}}{\sqrt{\boldsymbol{N}}}$ remains true. When this condition is satisfied in a specific node for the first time during a simulation, we consider that node to have reached a steady state in terms of $\boldsymbol{c}_{\boldsymbol{EI}}$ weight. If for a specific node, the absolute mean change of $\boldsymbol{c}_{\boldsymbol{EI}}$ in the last 10 minutes is smaller than the standard error of the mean in the same period, the value is considered stable. Since the rate of variation of $\boldsymbol{c}_{\boldsymbol{EI}}$ decreases until the local firing rate gets close to the target firing rate, $\boldsymbol{|mean(dc}_{\boldsymbol{EI,vec}}\boldsymbol{)|}$ will decrease until it approaches 0. However, to account for the stochasticity of the system, we compare the mean variation with its respective standard error. Therefore, we effectively detect when the tendency of variation caused by homeostatic plasticity trying to restore EI balance is smaller than changes caused by the inherent stochasticity of the model.

### Metastability, Synchrony and Peak Frequency

**
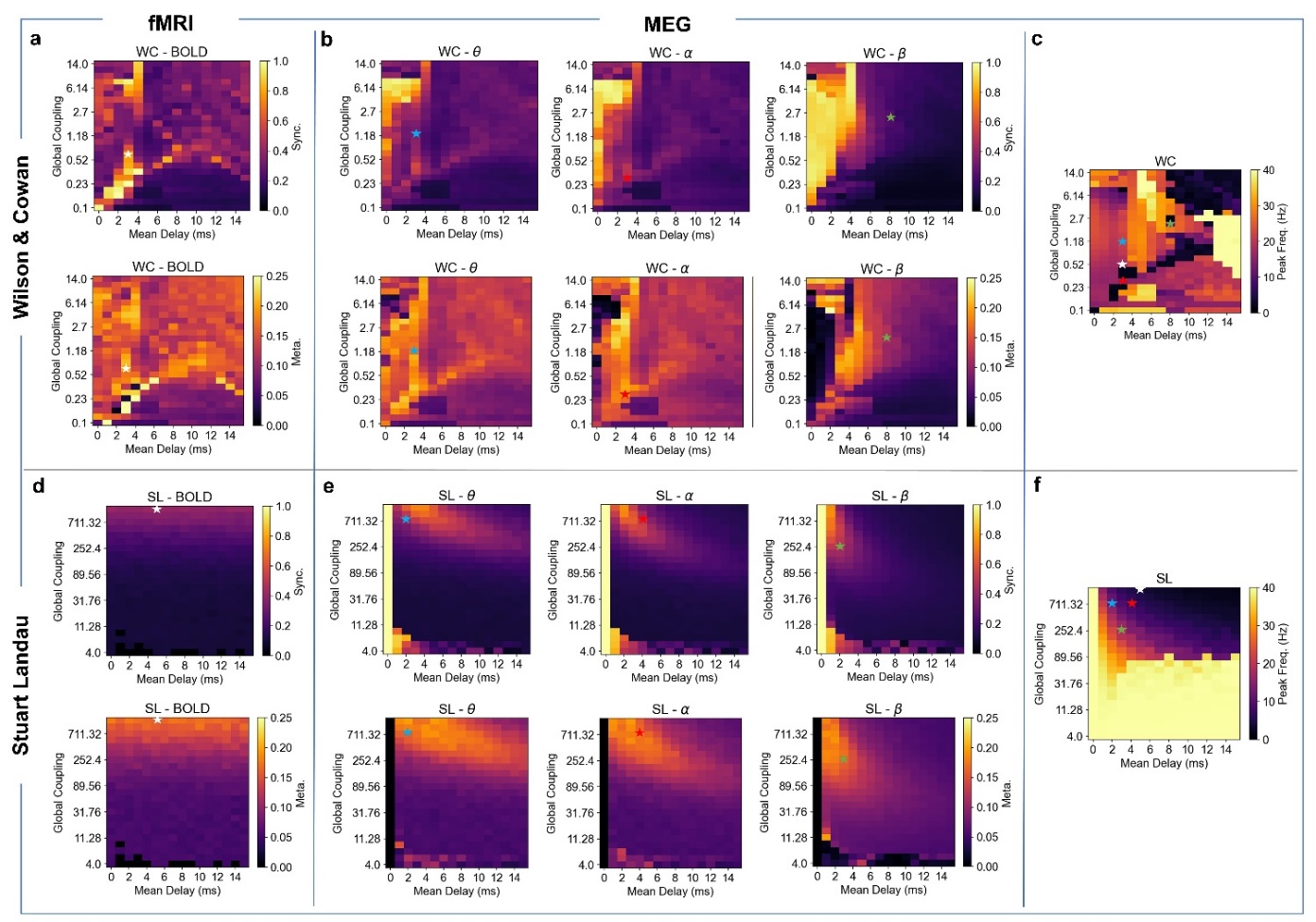
**

Figure S5. Metastability, Synchrony, Peak Frequency for SL and WC.

## SECTION III. Model Performance

### Role of inhibitory synaptic plasticity

**
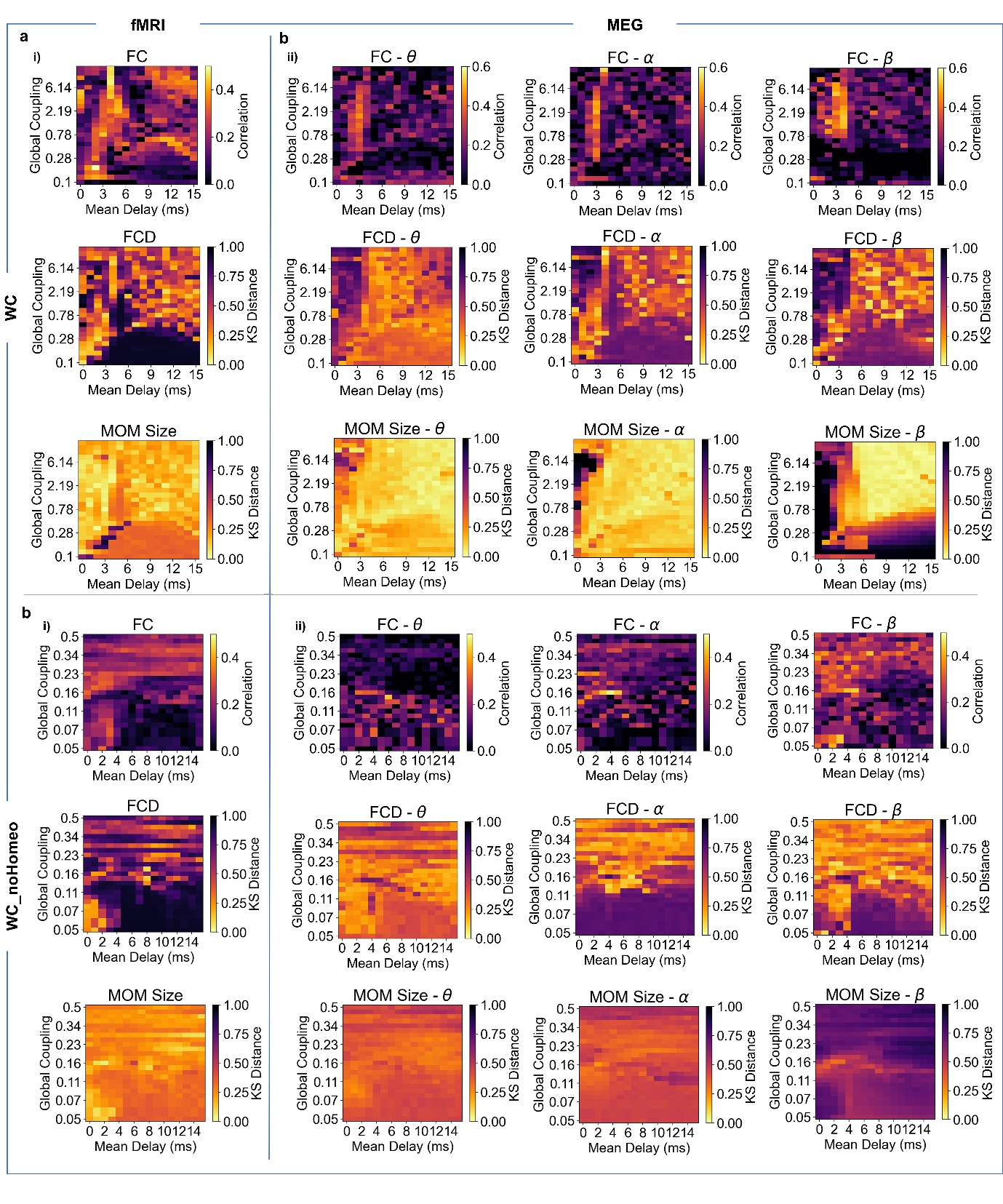
**

Figure S6. WC model performance with and without plasticity. a. i) Model performance in explaining empirical BOLD fMRI static connectivity measures. *Top.* Pearson correlation between BOLD fMRI FC (averaged across 99 HCP participants) and simulated FC for each pair of parameters (Mean Delay and Global Coupling) for WC model. *Middle.* Kolmogorov-Smirnov (KS) distance between empirical and simulated BOLD fMRI MOM Size distributions for each pair of WC model parameters. *Bottom.* Kolmogorov-Smirnov (KS) distance between empirical and simulated BOLD fMRI FCD distributions for each pair of WC model parameters. ii) Model performance in representing empirical MEG connectivity measures. *Top.* Pearson correlation between Hilbert envelope FC of MEG (averaged across 89 HCP participants) and simulated Hilbert envelope FC for each pair of parameters, for theta [4-8 Hz] (left), alpha [8-13Hz] (middle), beta [13-30Hz] (right) for WC model. *Middle.* Kolmogorov-Smirnov (KS) distance between empirical and simulated MEG MOM Size distributions for each pair of WC model parameters, for theta [4-8 Hz] (*left*), alpha [8-13Hz] (*middle*), beta [13-30Hz] (*right*) frequency bands. *Bottom.* Kolmogorov-Smirnov (KS) distance between empirical and simulated Hilbert envelope MEG FCD distributions for each pair of WC model parameters, for theta [4-8 Hz] (*left*), alpha [8-13Hz] (*middle*), beta [13-30Hz] (*right*). b. WC model performance without homeostatic plasticity. Same as above. In this context, the range of global couplings chosen differs because, for values of coupling higher than 0.5, most of the nodes in the network enter a saturated regime where the simulations are not physiologically plausible.

### Target firing rate


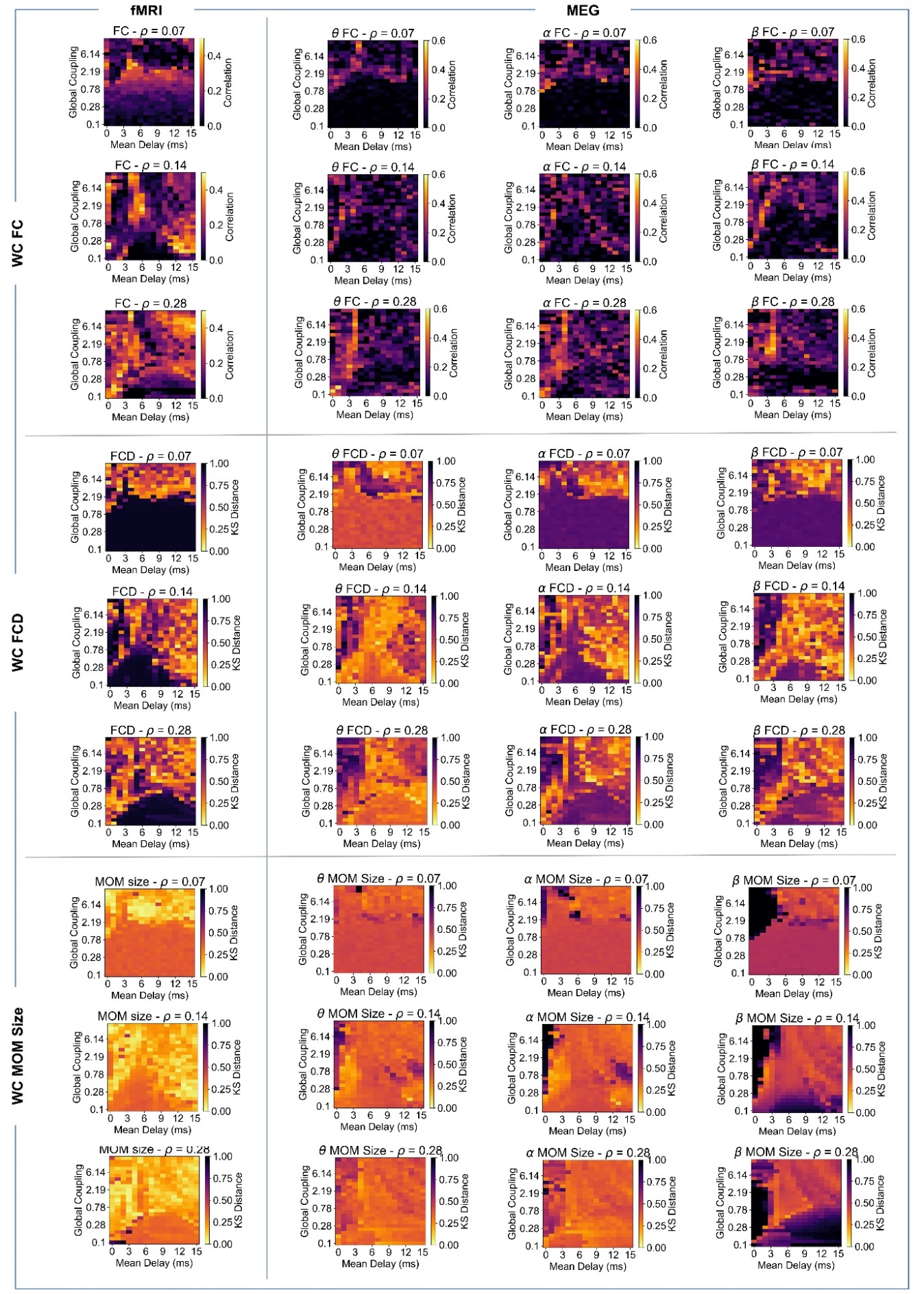


Figure S7. Wilson and Cowan model performance for different values of the target firing rate. Model performance in explaining empirical BOLD fMRI and MEG static and dynamic functional connectivity measures and MOM size for three different values of the target firing rate $\boldsymbol{\varrho}$, that is $\boldsymbol{\varrho=0.07}$, $\boldsymbol{\varrho=0.14}$, $\boldsymbol{\varrho=0.28}$.

### Analysis across features

Ability of the models in explaining three empirical features simultaneously, that is FC, FCD or MOM Size.


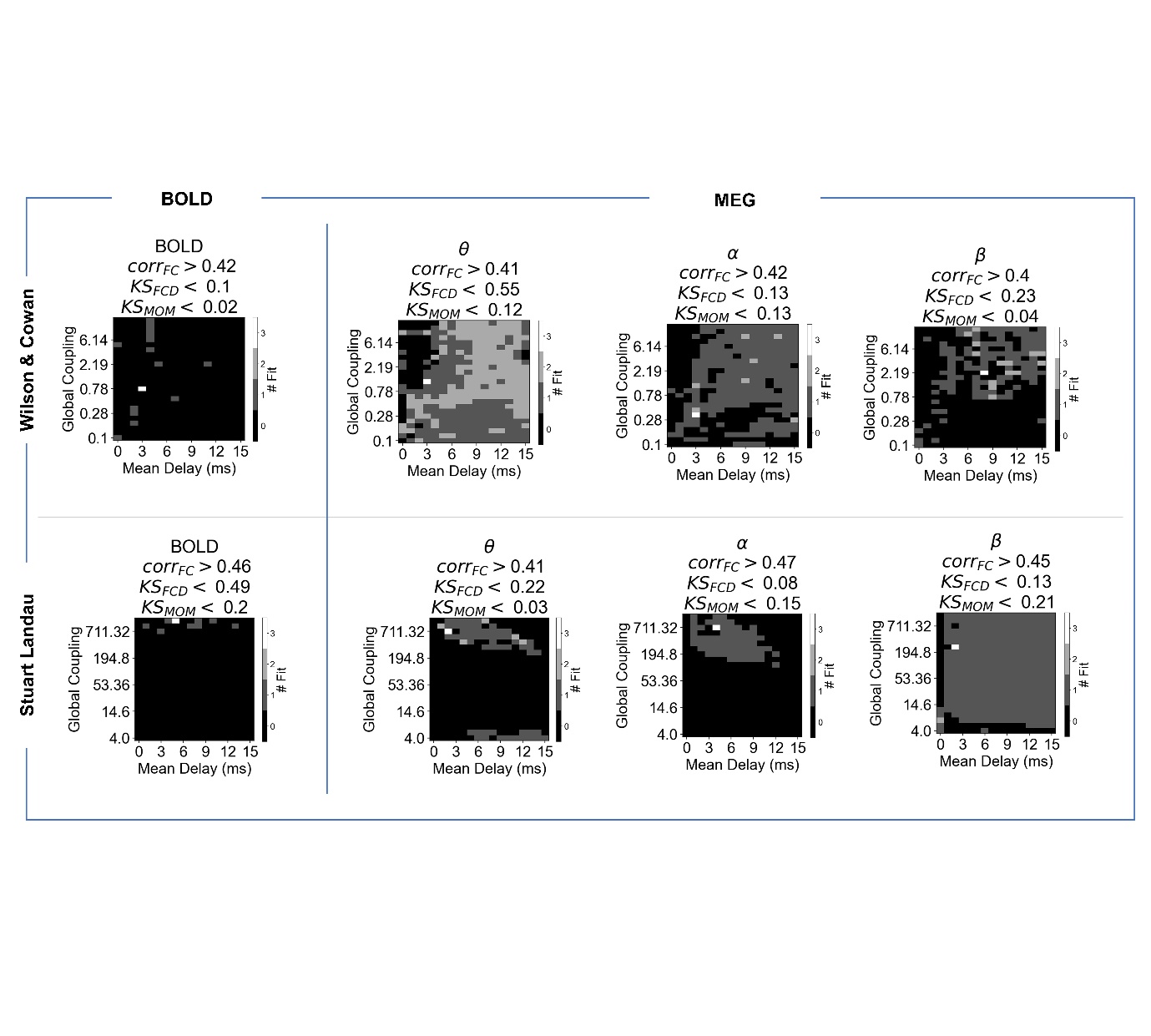


Figure S8. Cross-feature analysis with optimised thresholds.


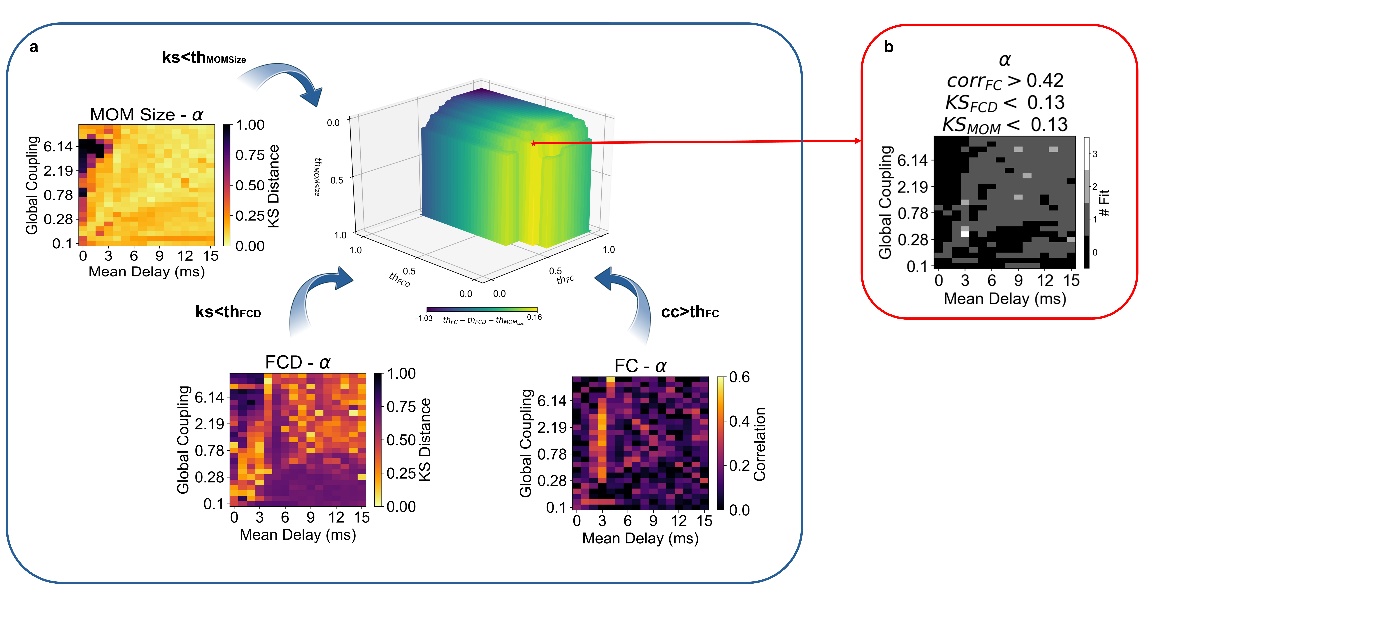


Figure S9. Selection of thresholds for the analysis across features. Example for only one modality (MEG in alpha band) and one model (WC). This method is consistently applied for all the other modalities and models. Optimal points are chosen by iterating over a range of thresholds for FC correlation (cc ≥ $\boldsymbol{th}_{\boldsymbol{FC}}$), FCD KS-distance (KS ≤ $\boldsymbol{th}_{\boldsymbol{FCD}}$), MOM size (KS ≤ $\boldsymbol{th}_{\boldsymbol{MOMSize}}$).

### Performance values optimised to represent FC, FCD or MOMs only.

###

|  | Best fit with delays, optimised for FC only | | | | Best fit with delays, optimised for FCD only | | | | Best fit with delays, optimised for MOMs size only | | | |
| --- | --- | --- | --- | --- | --- | --- | --- | --- | --- | --- | --- | --- |
|  | **fMRI** | **MEG θ** | **MEG** **α** | **MEG** **β** | **fMRI** | **MEG θ** | **MEG** **α** | **MEG** **β** | **fMRI** | **MEG θ** | **MEG** **α** | **MEG** **β** |
| WC | 0.529 | 0.458 | 0.546 | 0.525 | 0.082 | 0.130 | 0.036 | 0.042 | 0.018 | 0.018 | 0.023 | 0.01 |
| SL | 0.484 | 0.512 | 0.483 | 0.581 | 0.489 | 0.077 | 0.078 | 0.046 | 0.193 | 0.016 | 0.023 | 0.015 |

Table S1. Performance values, individually optimised for FC, FCD features and MOMs size, accounting for delays.

|  | Best fit without delays, optimised for FC only | | | | Best fit without delays, optimised for FCD only | | | | Best fit without delays, optimised for MOMs size only | | | | |
| --- | --- | --- | --- | --- | --- | --- | --- | --- | --- | --- | --- | --- | --- |
|  | **fMRI** | **MEG θ** | **MEG** **α** | **MEG** **β** | **fMRI** | **MEG θ** | **MEG** **α** | **MEG** **β** | **fMRI** | **MEG θ** | **MEG** **α** | **MEG** **β** |  |
| WC | 0.436 | 0.265 | 0.290 | 0.423 | 0.082 | 0.216 | 0.104 | 0.148 | 0.053 | 0.069 | 0.093 | 0.462 |  |
| SL | 0.458 | 0.269 | 0.267 | 0.489 | 0.584 | 0.311 | 0.711 | 0.126 | 0.205 | 1.000 | 1.000 | 1.000 |  |

Table S2. Performance values, individually optimised for FC, FCD features and MOMs size, without delays.

### Metastable Oscillatory Modes


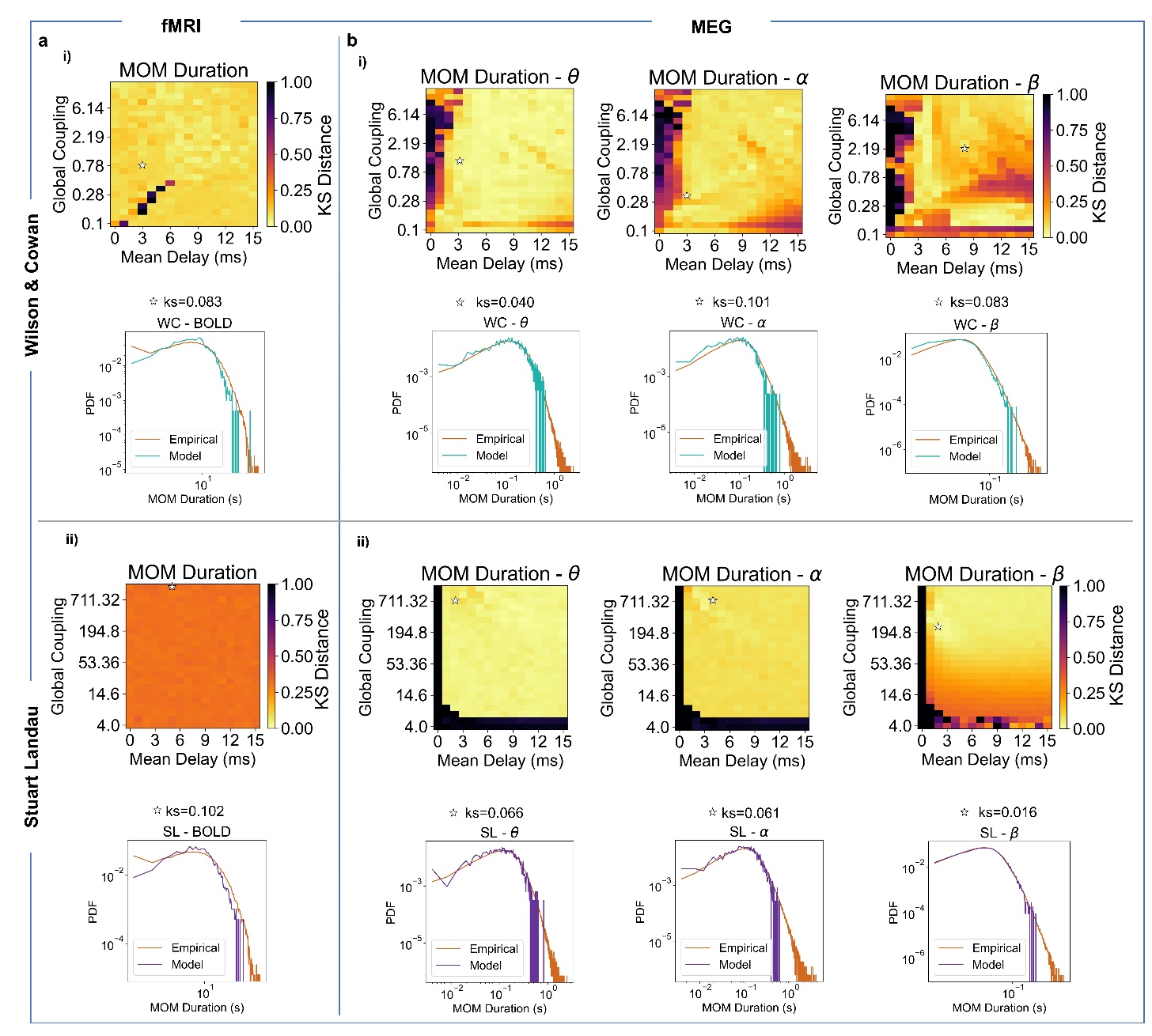


Figure S10. Models’ performance in approximating the duration of Metastable Oscillatory Modes. a. Kolmogorov-Smirnov (KS) distance between empirical BOLD fMRI MOM duration distribution (averaged across 99 HCP participants) and simulated MOM size distribution for each pair of parameters (Mean Delay and Global Coupling) for WC and SL model.  i) *Top -* For the WC model, the optimal parameters (white star) for BOLD fMRI are C=0.780, MD=3ms, with ks-distance value of ks=0.018. *Bottom –* Comparison between empirical and simulated BOLD fMRI MOM size distribution for 78 AAL cortical brain areas in the optimal point. ii) *Top -* For the SL model, the optimal points for BOLD fMRI are C=1194.16, MD=5ms, with ks-distance value of ks=0.193. *Bottom –* Comparison between empirical and simulated BOLD fMRI MOM size distribution for 78 AAL cortical brain areas in the optimal point. b. Model performance in representing empirical MEG MOM size: Kolmogorov-Smirnov (KS) distance between empirical MEG MOM size distribution (averaged across 89 HCP participants) and simulated MOM size distribution for each pair of parameters, for theta [4-8 Hz] (*left*), alpha [8-13Hz] (*middle*), beta [13-30Hz] (*right*) for WC and SL model. i) *Top –* For the WC model, the optimal points for MEG are C=1.183, MD=3ms for theta; C=0.344, MD=3ms for alpha; C=2.19, MD=8ms for beta with ks-distance values of ks_θ_ =0.118, ks_α_ =0.120, ks_β_ =0.032. *Bottom –* Comparison between empirical and simulated frequency-specific MEG MOM size distribution for 78 AAL cortical brain areas in the optimal point. ii) *Top -*For the SL model, the optimal points for MEG are C=711.32, MD=2ms for theta; C=711.32, MD=4ms for alpha; C=252.4, MD=2ms for beta with ks distance values of ks_θ_ =0.026, ks_α_ =0.149, ks_β_ =0.204. *Bottom –* Comparison between empirical and simulated frequency-specific MEG MOM size distribution for 78 AAL cortical brain areas in the optimal point. White stars indicate the model working points, chosen through simultaneous optimization for the reproduction of empirical FC, FCD and MOMs’ size, as described in the Methods section.


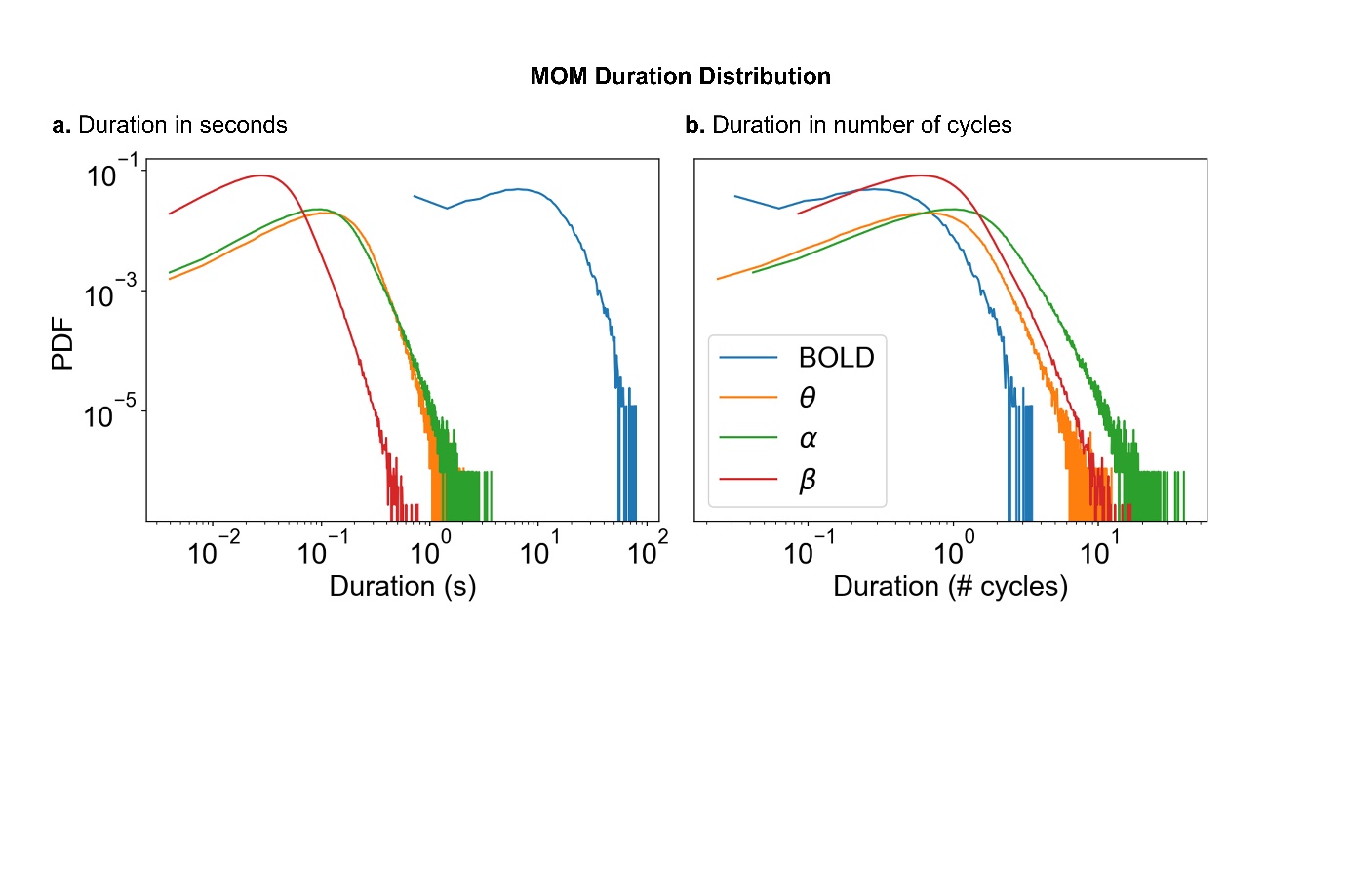


Figure S11. MOM’s duration distribution in senconds and number of cycles, for empirical data.


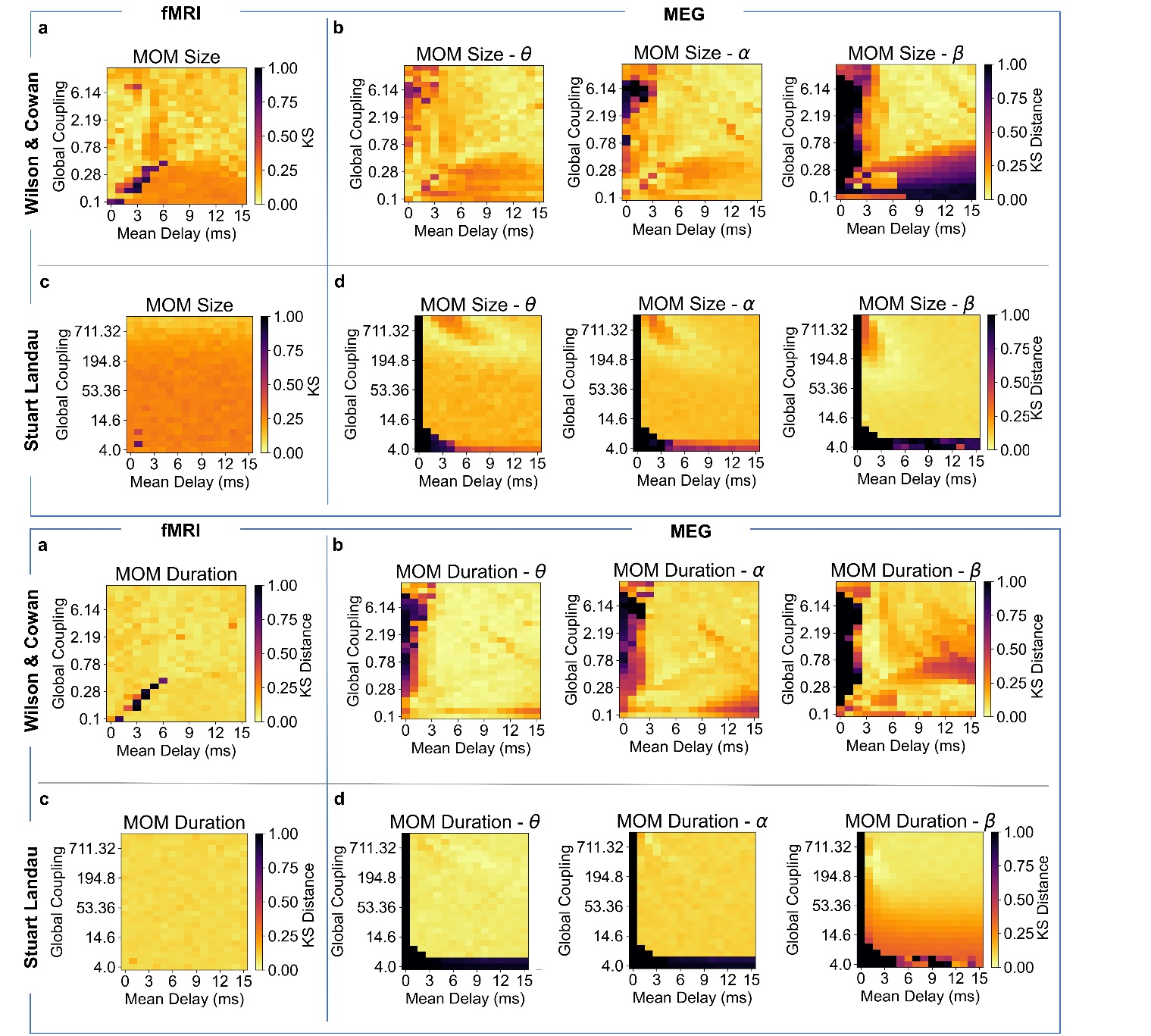


Figure S12. Models’ performance in explaining MOM size detected with a different threshold.


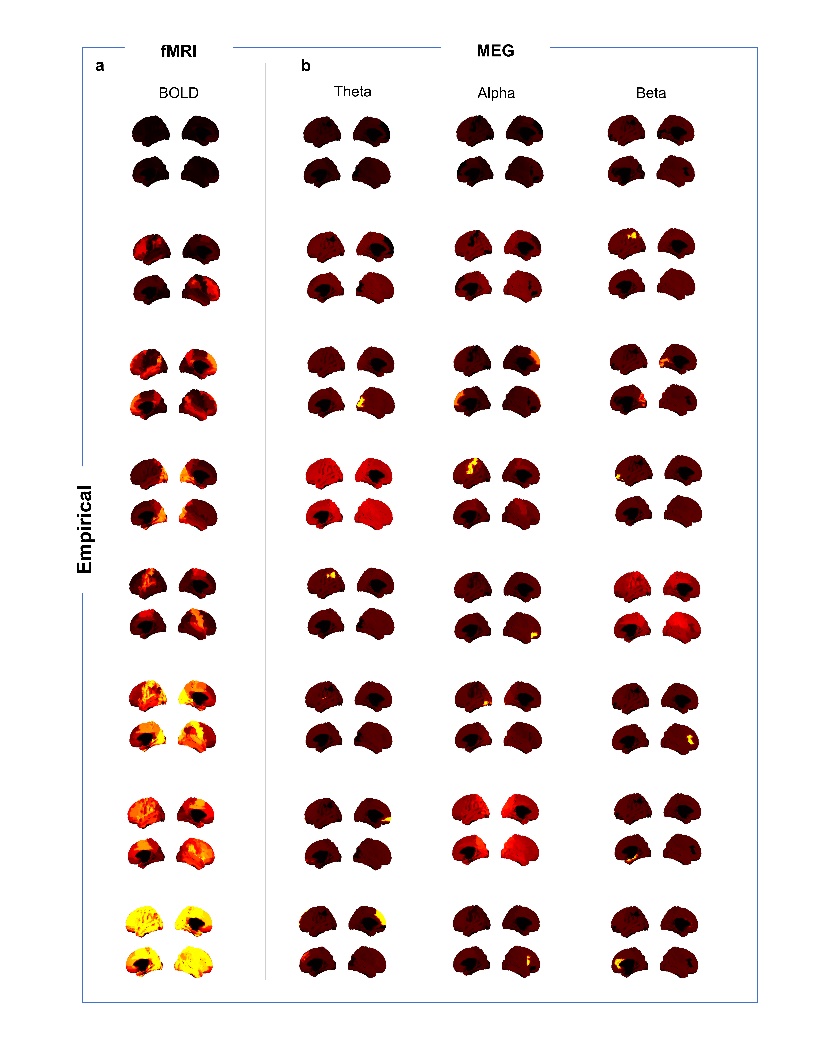

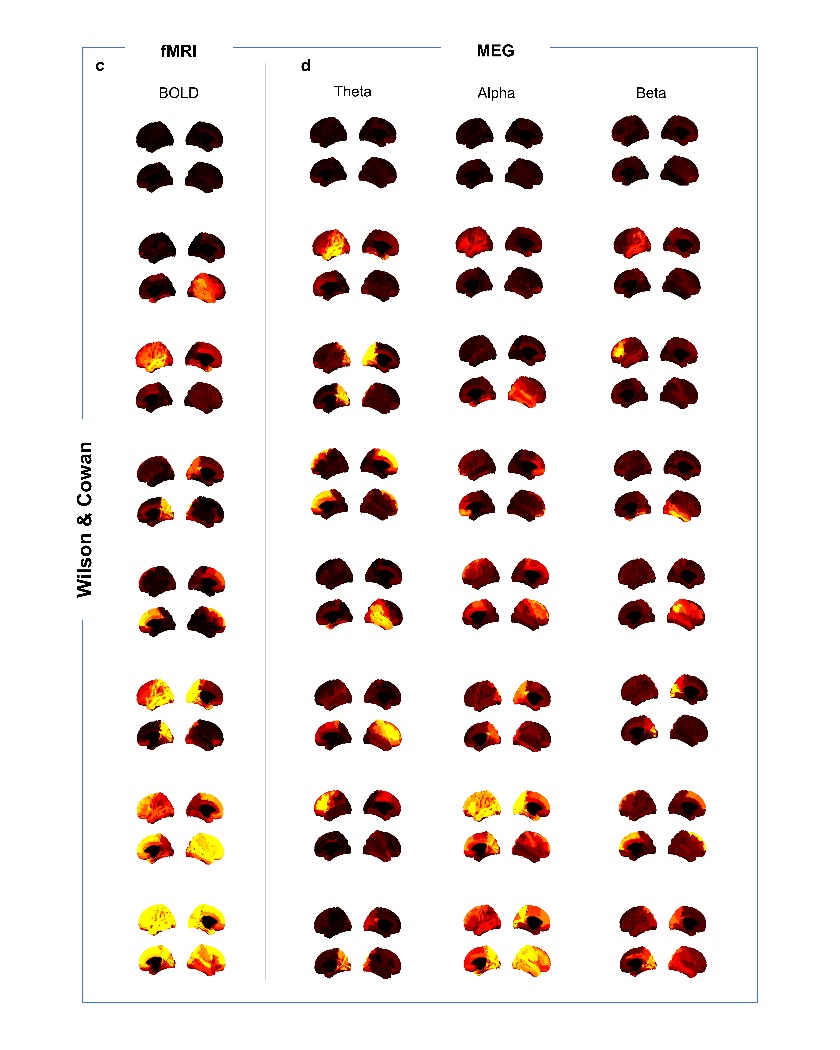

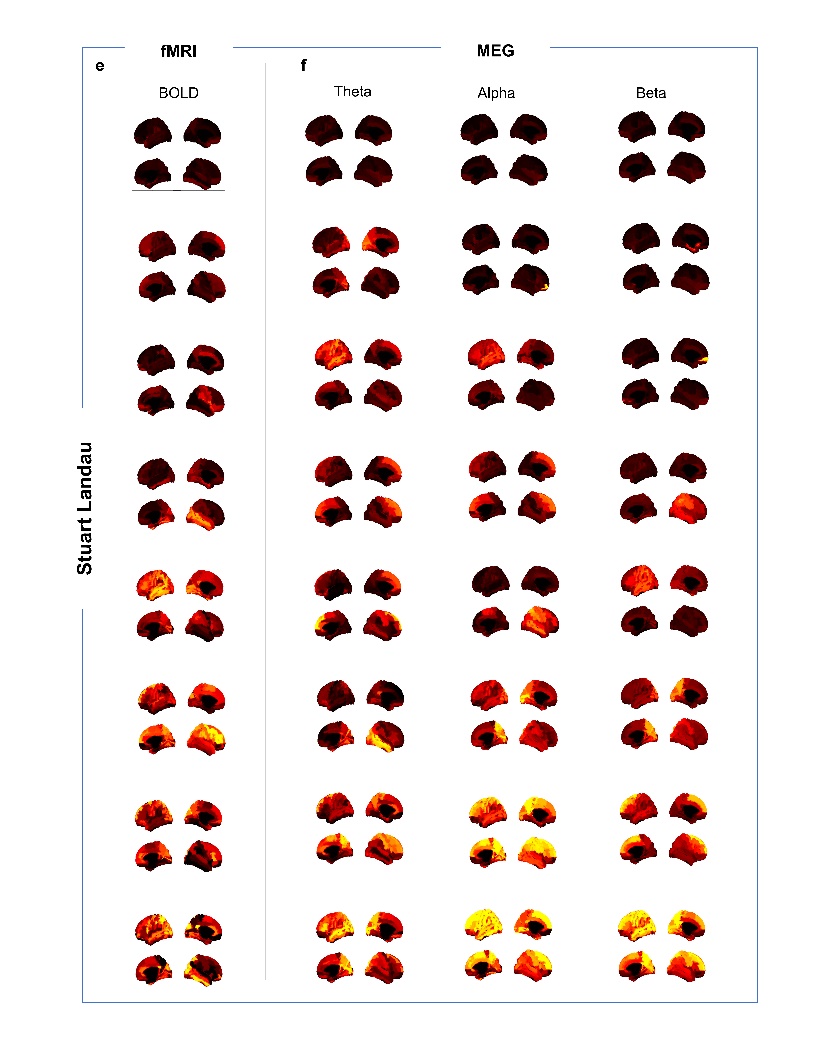


Figure S13. MOMs topographies.

##
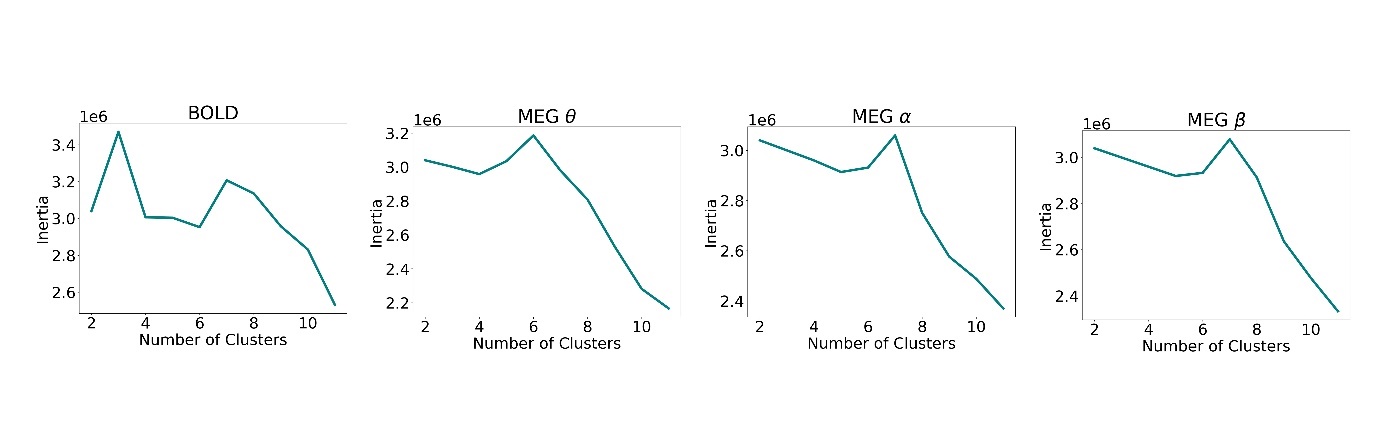


Figure S14. Clustering method to extract the optimal number of resting state networks (RSNs) for both models and modalities.

## SECTION IV

### Structural Connectivity

**
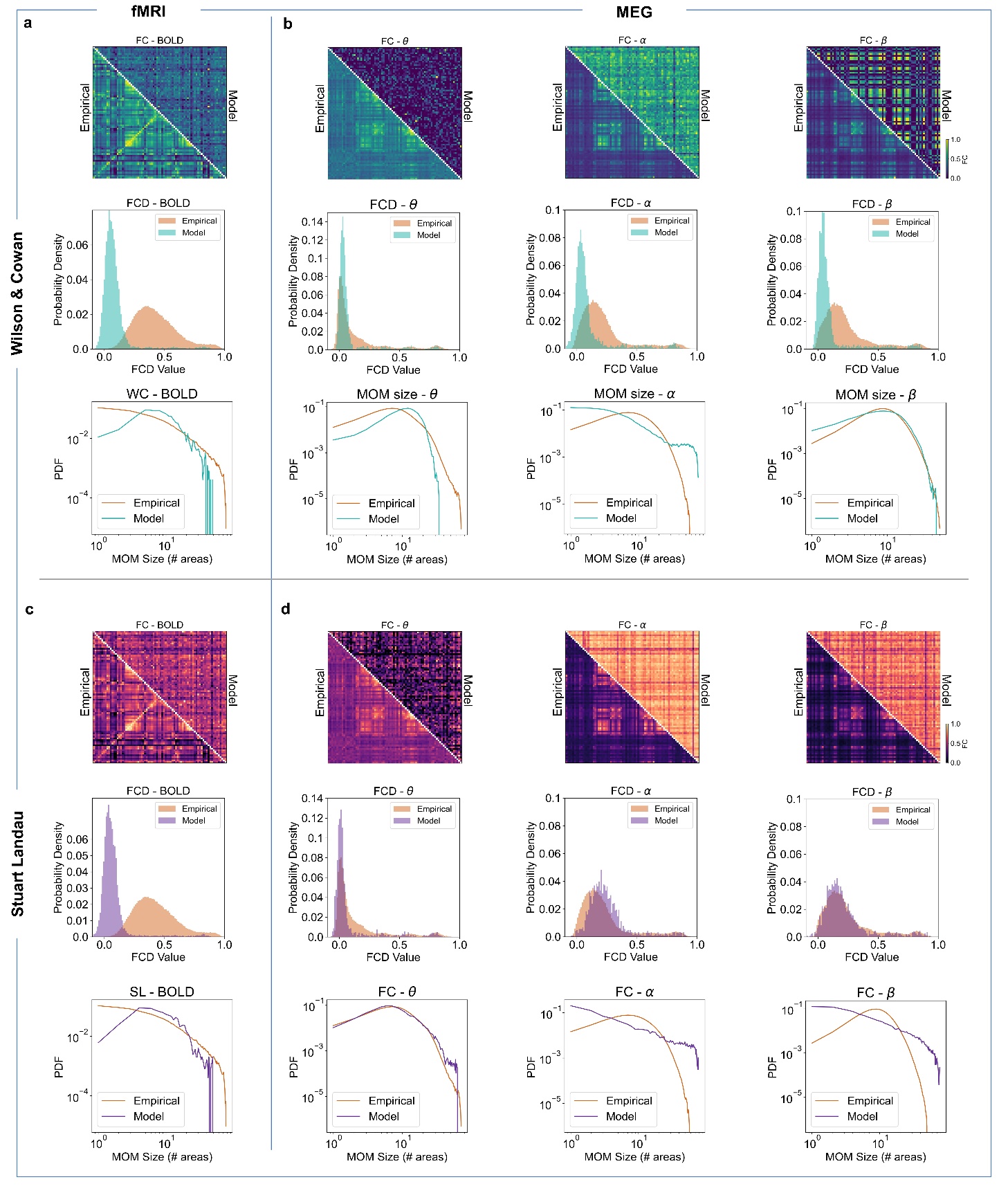
**

**Figure S15. WC and SL model performance giving a shuffled SC as input.** Empirical and simulated fMRI BOLD and MEG FC and FCD distributions. To prove the predictive validity of the connectome (without the right structure there is no emergence of functionally relevant patterns) we shuffled the input structural connectivity and plotted the results only for selected optimal points (white stars in Figures 2 and 3 of the main document).

**
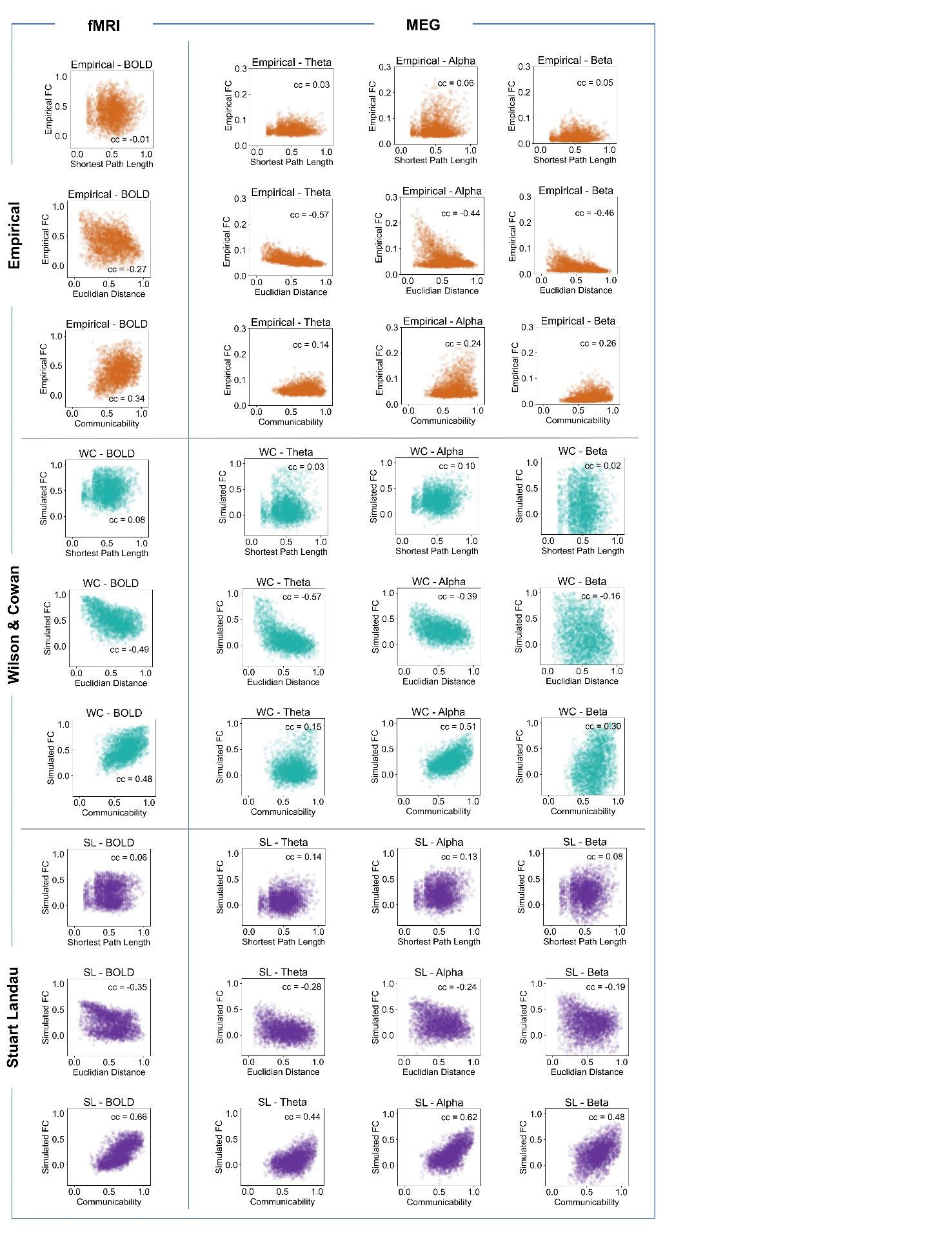
**

Figure S16. Functional Connectivity versus Network measures for different scenarios and modalities. Scatter plots show the relationship between FC and three different network measures, namely shortest path length, Euclidean distance, and communicability, for empirical, WC and SL model. Notably, the FC here is extracted from a point in the parameter space optimised across all the features (FC, FCD and MOMs size).


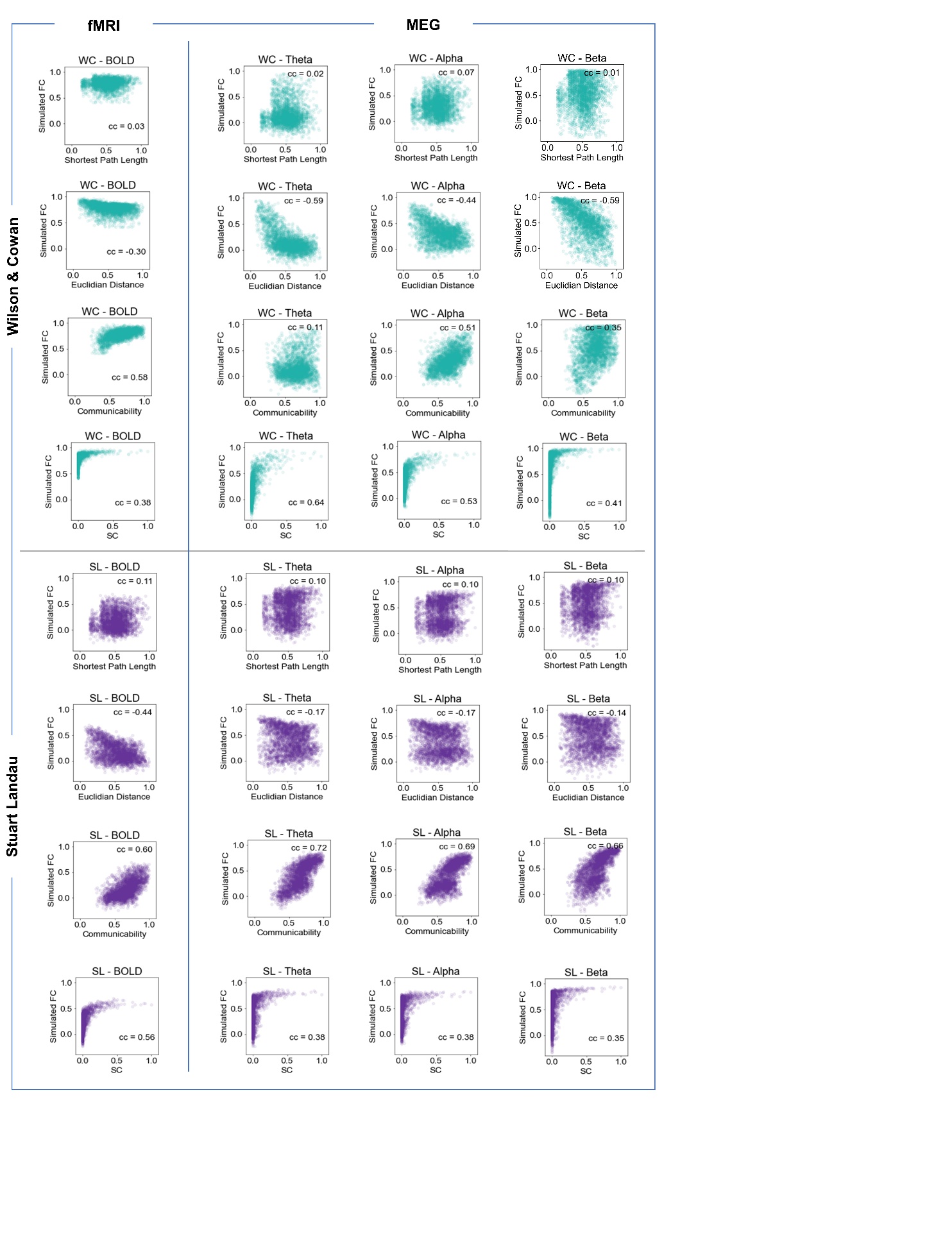


Figure S17. Functional Connectivity versus Network measures for different scenarios and modalities (Optimised for FC only). Scatter plots show the relationship between FC and three different network measures, namely shortest path length, Euclidean distance, communicability, and SC for WC and SL model. Notably, the FC here is extracted from a point in the parameter space optimised for FC only.

| Idx | Area | Idx | Area | Idx | Area | Idx | Area |
| --- | --- | --- | --- | --- | --- | --- | --- |
| 1 | L Precentral | 21 | L Olfactory | 41 | L Cuneus | 61 | L Angular |
| 2 | R Precentral | 22 | R Olfactory | 42 | R Cuneus | 62 | R Angular |
| 3 | L Frontal Sup | 23 | L Front Sup Med | 43 | L Lingual | 63 | L Precuneus |
| 4 | R Frontal Sup | 24 | R Front Sup Med | 44 | R Lingual | 64 | R Precuneus |
| 5 | L Front Sup Orb | 25 | L Front Med Orb | 45 | L Occipital Sup | 65 | L Paracentr Lob |
| 6 | R Front Sup Orb | 26 | R Front Med Orb | 46 | R Occipital Sup | 66 | R Paracentr Lob |
| 7 | L Front Mid | 27 | L Rectus | 47 | L Occipital Mid | 67 | L Heschl |
| 8 | R Front Mid | 28 | R Rectus | 48 | R Occipital Mid | 68 | R Heschl |
| 9 | L Front Mid Orb | 29 | L Insula | 49 | L Occipital Inf | 69 | L Temporal Sup |
| 10 | R Front Mid Orb | 30 | R Insula | 50 | R Occipital Inf | 70 | R Temporal Sup |
| 11 | L Front Inf Ope | 31 | L Cingulum Ant | 51 | L Fusiform | 71 | L Tempr Pol Sup |
| 12 | R Front Inf Ope | 32 | R Cingulum Ant | 52 | R Fusiform | 72 | R Tempr Pol Sup |
| 13 | L Front Inf Tri | 33 | L Cingulum Mid | 53 | L Postcentral | 73 | L Temporal Mid |
| 14 | R Front Inf Tri | 34 | R Cingulum Mid | 54 | R Postcentral | 74 | R Temporal Mid |
| 15 | L Front Inf Orb | 35 | L Cingulum Post | 55 | L Parietal Sup | 75 | L Tempr Pol Mid |
| 16 | R Front Inf Orb | 36 | R Cingulum Post | 56 | R Parietal Sup | 76 | R Tempr Pol Mid |
| 17 | L Rolandic Oper | 37 | L ParaHippocamp | 57 | L Pariental Inf | 77 | L Temporal Inf |
| 18 | R Rolandic Oper | 38 | R ParaHippocamp | 58 | R Parietal Inf | 78 | R Temporal Inf |
| 19 | L Supp Motor Ar | 39 | L Calcarine | 59 | L SupraMarginal |  |  |
| 20 | R Supp Motor Ar | 40 | R Calcarine | 60 | R SupraMarginal |  |  |

Table S3. Cortical brain regions (N=78) and their corresponding numerical designation according to AAL parcellation.

## SECTION V

### MEG Analysis

#### Amplitude Envelope Correlation

The analytical signal vector, $z=[z_{1},z_{2} , ..., z_{T}]^{T}$ , of some temporal signal, $x=[x_{1},x_{2},...,x_{T}]^{T}$ , is defined as

|  | $z=x+iH[x]$ | (S1) |
| --- | --- | --- |

where $i$ is the imaginary number, and $H[x]$ is the *Hilbert* transform of the original signal. In words, the Hilbert transform provides a means of turning some real signal, x, into an analytical signal (comprising of real and imaginary parts), z, where the complex part of the analytical signal is given by the Hilbert transform of the real part of the signal. The “Hilbert Envelope”, $e=[e_{1},e_{2} , ..., e_{T}]^{T}$, of the source time series is then given by taking the absolute value of the complex output of the Hilbert transform.

If the Hilbert envelopes for brain regions A and B both have a mean of zero, the correlation between those regions is given simply by

|  | $r_{A,B}=e_{A}e_{B}=e_{A}{e^{T}}_{B}(\sqrt{e_{A}{e^{T}}_{A}e_{B}{e^{T}}_{B}})^{-1}$, | (S2) |
| --- | --- | --- |

where $e_{A}$ and $e_{B}$ are the envelopes of brain regions A and B, respectively. Repeated application of this formula between envelopes distributed across the entire brain allows us to construct functional connectivity matrices of whole brain activity, $C_{E}\in R^{regions\times regions}$.

#### The M/EEG inverse problem and source leakage

The ill-posed nature of the inverse problem limits the spatial and temporal reconstruction accuracy of M/EEG signals, where extracranial measurements are used to approximate the activity at (typically) thousands of brain locations in a linear fashion.

In order to derive whole brain networks with M/EEG, one must address the ill-posed inverse problem. This is typically done before deriving FC metrics, although some generative FC frameworks effectively solve the inverse problem simultaneously alongside inferring FC, i.e. in Dynamic Causal Modelling (DCM) approaches (Friston et al., 2012).

Moreover, brain activity from one set of voxels can “leak” into a neighbouring set of voxels and results in artefactual zero-lag correlations featuring in reconstructed voxel time series. This leakage represents non-genuine FC between brain areas. This artefact has to be corrected for by orthogonalizing reconstructed time series via a singular value decomposition (Colclough et al., 2015) or through other means (Brookes et al., 2011; Hipp et al., 2012).

#### The role of zero-lag correlations in the models

Figure S18. Zero-lag correlations across the parameter space for SL and WC model.


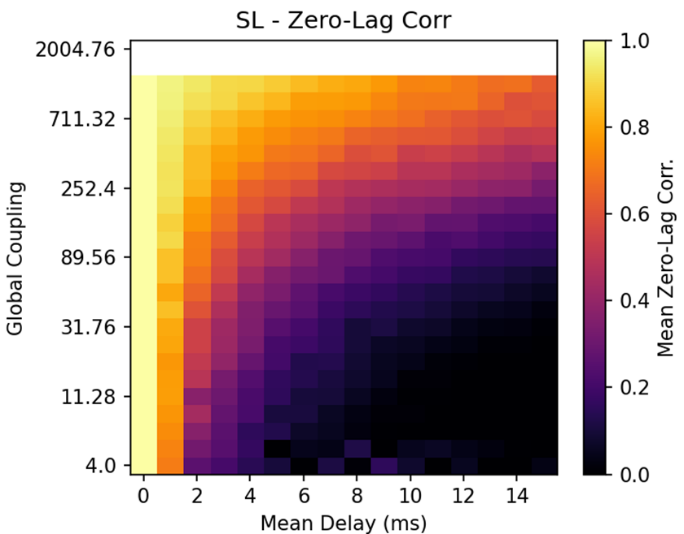

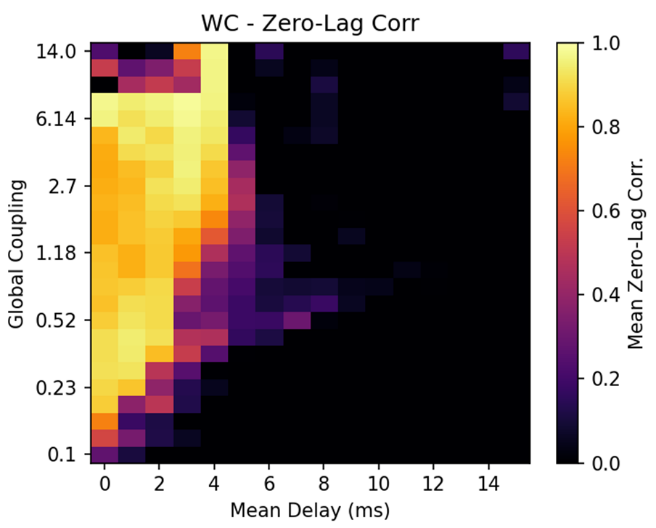


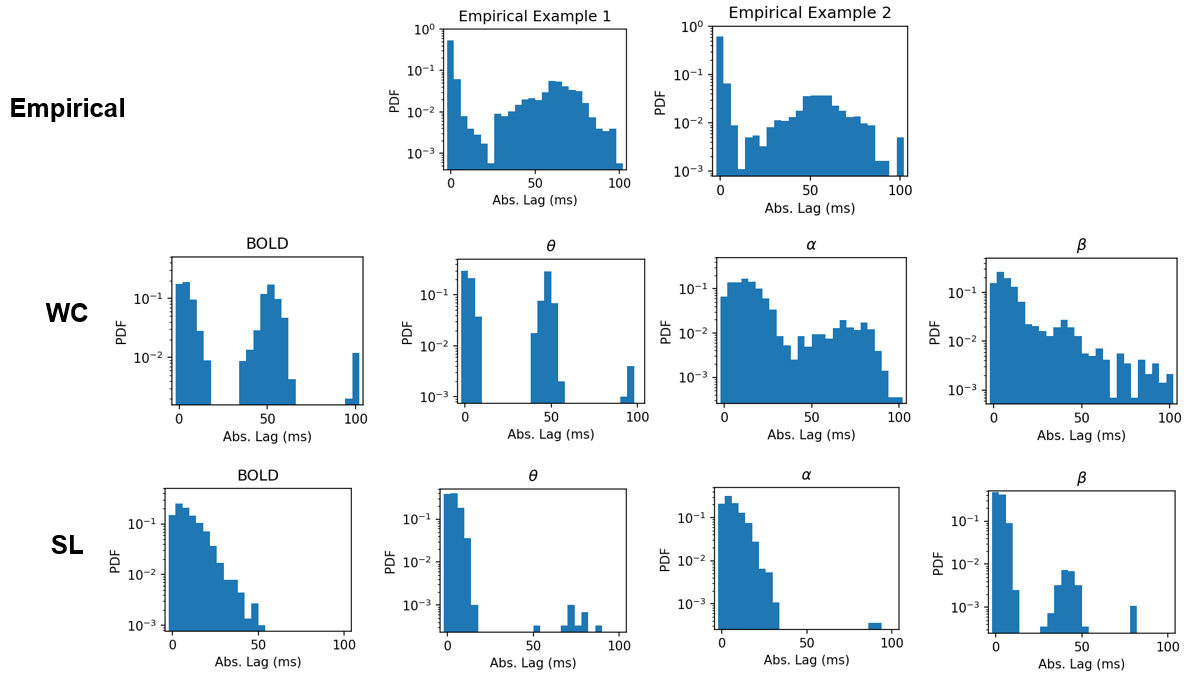


#### **Figure S19. Distribution of absolute lags corresponding to the peak of the correlation function across all pairs of nodes.**

#### MEG Source Reconstruction via Beamforming

One way of tackling the M/EEG inverse problem is via beamforming. Beamformers are a data-driven spatial filters which opt to estimate a particular signal of interest arriving at some sensor array in the presence of noise (Van Veen and Buckley, 1988). A spatial filter exploits the fact that desired and interfering signals tend to originate from different positions in space. With accurate forward modelling, this spatial separation can be exploited to separate signals of interest from noise.

The general beamformer equation assumes that neural signal at voxel $i$ can be reconstructed as a weighted linear sum of sensor level measurements,

|  | $\hat{x_{i}(t)}={w_{i}}^{T}y(t),$ | (S3) |
| --- | --- | --- |

where $w_{i}\in R^{1\times channels}$ are the beamformer weights optimised for voxel $i$.

The linearly constrained minimum variance (LCMV) beamformer opts to minimise the overall power of the source reconstructed timeseries, with the constraint that a signal with unit amplitude originating from that voxel provides a unit response, i.e. ${w^{T}}_{i}h_{i}=1$, where $h_{i}$ is a column of the lead field matrix which describes the magnetic field that we would expect to measure at the sensor level, given a unit source placed at a specified location and orientation. The solution to these constraints is

|  | $w_{i}=\frac{{C^{-1}}_{Y}h_{i}}{{h^{T}}_{i}{C^{-1}}_{Y}h_{i}}$ | (S4) |
| --- | --- | --- |

where $C_{Y}\in R^{channels\times channels}$ is the empirical sensor level covariance matrix.

# References

Brookes, M. J., Hale, J. R., Zumer, J. M., Stevenson, C. M., Francis, S. T., Barnes, G. R., Owen, J. P., Morris, P. G., & Nagarajan, S. S. (2011). Measuring functional connectivity using MEG: methodology and comparison with fcMRI. *NeuroImage*, *56*(3), 1082-1104. <https://doi.org/10.1016/j.neuroimage.2011.02.054>

Colclough, G. L., Brookes, M. J., Smith, S. M., & Woolrich, M. W. (2015). A symmetric multivariate leakage correction for MEG connectomes. *NeuroImage*, *117*, 439-448. <https://doi.org/10.1016/j.neuroimage.2015.03.071>

Friston, K. J., Bastos, A., Litvak, V., Stephan, K. E., Fries, P., & Moran, R. J. (2012). DCM for complex-valued data: cross-spectra, coherence and phase-delays. *NeuroImage*, *59*(1), 439-455. <https://doi.org/10.1016/j.neuroimage.2011.07.048>

Hipp, J. F., Hawellek, D. J., Corbetta, M., Siegel, M., & Engel, A. K. (2012). Large-scale cortical correlation structure of spontaneous oscillatory activity. *Nat Neurosci*, *15*(6), 884-890. <https://doi.org/10.1038/nn.3101>
